# Precision multidimensional neural population code recovered from single intracellular recordings

**DOI:** 10.1038/s41598-020-72936-1

**Published:** 2020-09-29

**Authors:** James K. Johnson, Songyuan Geng, Maximilian W. Hoffman, Hillel Adesnik, Ralf Wessel

**Affiliations:** 1 Washington University in St. Louis, St. Louis, USA; 2 University of California, Berkeley, Berkeley, USA

**Keywords:** Dynamical systems, Neural decoding, Neural encoding, Applied mathematics

## Abstract

Neurons in sensory cortices are more naturally and deeply integrated than any current neural population recording tools (e.g. electrode arrays, fluorescence imaging). Two concepts facilitate efforts to observe population neural code with single-cell recordings. First, even the highest quality single-cell recording studies find a fraction of the stimulus information in high-dimensional population recordings. Finding any of this missing information provides proof of principle. Second, neurons and neural populations are understood as coupled nonlinear differential equations. Therefore, fitted ordinary differential equations provide a basis for single-trial single-cell stimulus decoding. We obtained intracellular recordings of fluctuating transmembrane current and potential in mouse visual cortex during stimulation with drifting gratings. We use mean deflection from baseline when comparing to prior single-cell studies because action potentials are too sparse and the deflection response to drifting grating stimuli (e.g. tuning curves) are well studied. Equation-based decoders allowed more precise single-trial stimulus discrimination than tuning-curve-base decoders. Performance varied across recorded signal types in a manner consistent with population recording studies and both classification bases evinced distinct stimulus-evoked phases of population dynamics, providing further corroboration. Naturally and deeply integrated observations of population dynamics would be invaluable. We offer proof of principle and a versatile framework.

## Introduction

Pyramidal neurons aggregate population synaptic transmissions and neuromodulatory information then pass limited but useful information to downstream neuronal populations via action potentials. Experimentally accessing such upstream information would allow researchers to analyze the behavior of an intrinsically unambiguous neuroanatomical subpopulation: the group of neurons that synapse onto the same neuron (or neurons)^1^. Replicating findings about neural code in presynaptic population dynamics would be valuable because existing population recording methods, (e.g. electrode array or imaging methods) capture an outsider’s perspective of neural populations. They record from groups with distributions of neuron types and different functions (e.g. tunings) that may not be consistent with the distributions within intrinsic neural groups.

Unfortunately, the transformation of presynaptic population dynamics into synaptic input and then to neural output is very messy. Synaptic inputs can interfere with each other and are obscured by single-neuron biophysical effects, such as membrane properties^1–3^. Whole-cell recording methods observe these as fluctuations of current and potential at the neuronal cell body, i.e., the soma. Even the recording method complicates matters. The cell membrane must be held at a fixed voltage to record transmembrane currents, but distal dendrites may be poorly controlled^4^. No existing methods are reliable for even partially reconstructing the rich information in presynaptic population dynamics from transmembrane potential and current fluctuations.

Fortunately, there is an ongoing revolution in data analysis^5^ and neuronal recording methods^6,7^. Even without machine learning, network-state signatures like scale-freeness can be uncovered with long enough recordings^8^. However, it would be more useful to get to detailed information about presynaptic population dynamics from fluctuations on short timescales. To test whether useful population information can be extracted from single-trials of single-cell recordings we use a stimulus discrimination challenge. Stimulus discrimination is a familiar classification-type machine learning problem. The spike trains of individual neurons discriminate stimulus poorly on a single trial basis because spiking is too sparse. Deflection from baseline of membrane potential and transmembrane current has been related to presynaptic populations^9^ and can improve discrimination somewhat while preserving the “tuning curve” stimulus encoding found in single neuron spike trains. We assert that any method which is agnostic to tuning curves and shows any increased discriminability demonstrates that one has “decoded” stimulus information from presynaptic populations, rather than or in addition to decoding single-neuron output. Most machine learning methods are unsuitable because usually a small number of recordings are available. As a rule of thumb, the number of parameters in the model must be less than the number of examples used in training. However, the number of parameters in the model can never be less than the number of data points in each example (e.g. the number of time points in a recording). Therefore, we need a very compact representation of neural time-series before proceeding with classification. In a best-case scenario this representation should be routed in neuroscience, have a clear interpretation, and have intrinsic value for testing hypotheses about neural code. Neuronal population dynamics are often characterized by systems of differential equations^10,11^ which have few terms. Therefore, time series analysis and latent variable discovery may be the missing elements needed for extracting detailed population information within single trials of single-neuron intracellular recordings.

The attractor network paradigm^12–15^, provides a hypothetical connection between ordinary differential equation models (ODEs) and stimulus decoding. This paradigm is novel in primary visual cortex^16,17^. It describes brain activity as trajectories in a high-dimensional state-space and maps trajectory characteristics like shape or location to specific brain functions (e.g. memory, movement, or recognition^18^). Attractor network dynamics exhibit attracting sets representing brain operations, i.e. a stimulus evokes a perturbation causing network dynamics to either explore state space near a different fixed point, or to undergo a bifurcation. A fixed point is a point (or manifold of points) where all derivatives are zero. Near a fixed point most dynamical systems fall into a simple, or at least quasiperiodic, dynamical pattern (e.g. limit cycle oscillations, monotonic convergence)^19^, and similar initial conditions produce similar trajectories over short timescales unless a dynamical bifurcation has occurred. A bifurcation is a sudden change in the character of fixed points, this also causes a sudden change in the repertoire of possible population dynamics. Thus, if a shift in stimulation also shifts dynamics to a different fixed point or causes a bifurcation, then brief snippets from trajectories co-occurring with different stimuli will have different characteristics.

**Figure 1:**
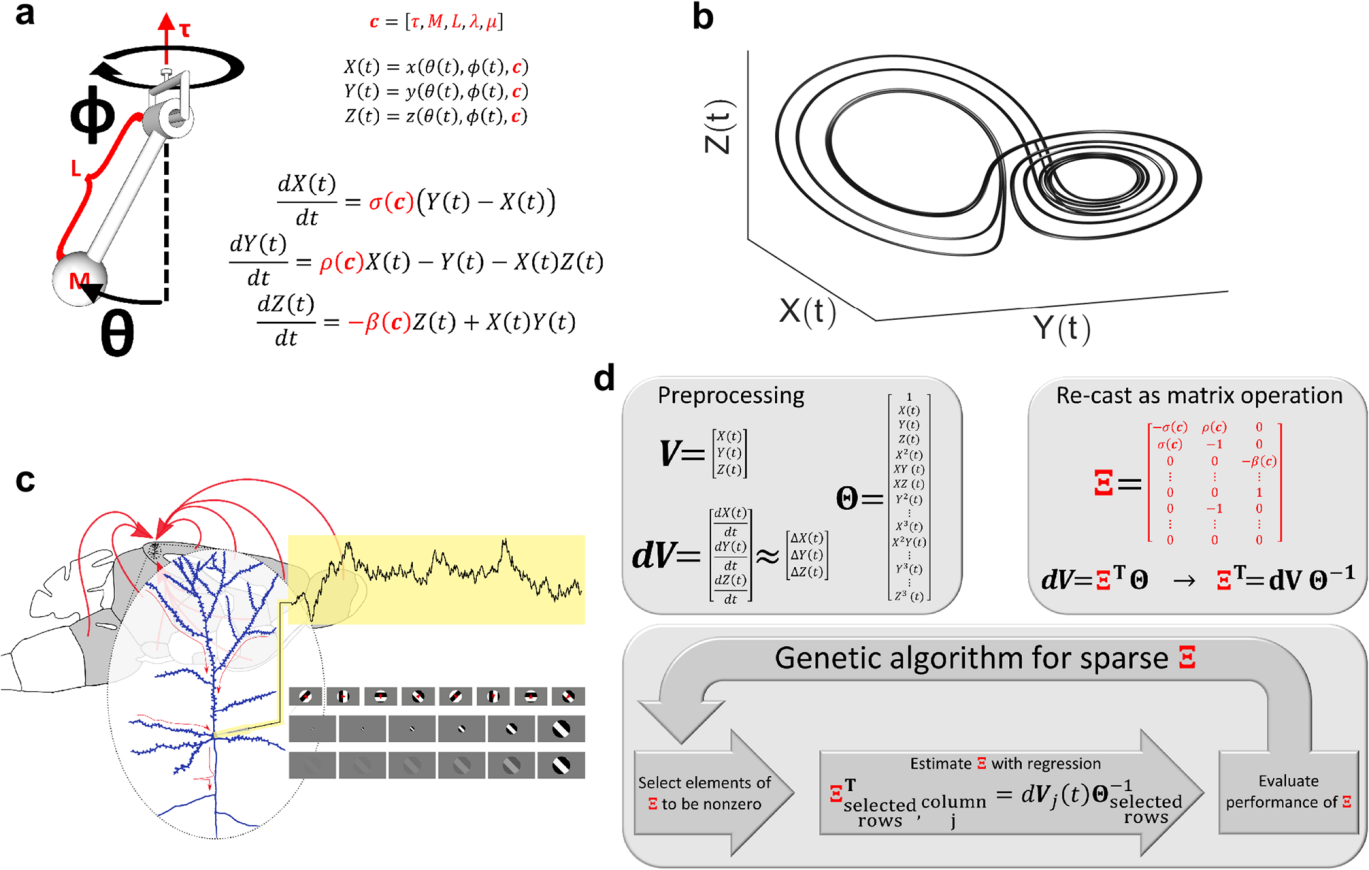
The context of a dynamical trajectory is represented in governing equation parameters. (**a**) A pendulum governed by Lorenz equations after changing variables from spherical coordinates (θ, ϕ, r) to abstract coordinates (X, Y, Z)^20^. Our interpretation of “context” is illustrated by the parameters: constant torque τ, bob mass M, rod length L, Stoke’s Law coefficient λ, and friction μ. (**b**) A chaotic Lorenz attractor (σ = 10, β = 8/3, ρ = 28. (**c**) An illustration of our data. *Source*: Intracellular recordings from single neurons in mouse V1. The context of recorded dynamics is influenced by visual stimulation. We have three stimulus categories: eight drifting grating orientations (top), six logarithmically spaced sizes (center), and six logarithmic contrast levels (bottom). This original composite image includes an adapted brain atlas from The Gene Expression Nervous System Atlas (GENSAT) Project, NINDS Contracts N01NS02331 & HHSN271200723701C to The Rockefeller University (New York, NY). (d) We identify context through modified Sparse Identification of Nonlinear Dynamics (SINDy). We illustrate a context modulated sparse dynamical systems coefficient matrix, Ξ, with the Lorenz system (see a). V is a time-delay embedding of a single dynamical variable (e.g. membrane potential). Θ contains all polynomial combinations of V up to cubic terms. dV is a derivative estimate. Ξ, is learned through linear regression to regress Θ onto dV. Ξ represents our data compactly enough to train classifiers to predict context (e.g. stimulus) with few examples. A genetic algorithm finds Ξ by picking different sets of nonzero elements at each generation. The best set of nonzero elements is chosen based on whether Ξ leads either to the best stimulus classification performance or best trajectory reconstruction while maintaining sparseness.

Although equations completely governing a network have fixed parameters, approximations and reductions of governing equations have different parameters near different fixed points. Thus, stimuli may modulate the parameters of rudimentary equations fitted to brief snippets of evoked activity. This modulation is illustrated in Fig. 1a by pendulum with physical properties which are like stimulus characteristics providing the context for a recording of neural activity. Upon change of coordinates, the pendulum's motion is a simple Lorenz system^20^ (Fig. 1b) with coefficients modulated by physical properties (i.e. context).

To test our dynamical systems insights, we obtained recordings of membrane current and potential from single neurons in primary visual cortex of awake mice concurrent with visual stimulation by drifting gratings of varying orientation, size, and contrast (Fig. 1c). This data was collected to support a prior publication^21^. We applied a Whitney–Takens time-delay based dimensionality expansion^22–24^ to one-dimensional intracellular recordings. This is analogous to dimensionality reduction methods such as PCA and results in moderate dimension neural trajectories that represent a projection of a putative high-dimensional dynamical system (population dynamics) onto a lower dimensional space. We then applied a model discovery algorithm^25^ (Fig. 1d) modified to interpret the coefficients of differential equations as a compact representation of neural time series and thus a basis for stimulus discrimination. Because dynamical systems are used as features in a stimulus decoder, we call the algorithm “dynamical discrimination”.

We found that dimensionality expansion of our intracellular recordings followed by dynamical discrimination permitted better than chance classification of small changes in orientation, size, and contrast of drifting gratings. Overall correct classification rates exceeded tuning-curve classifiers based on deflections of transmembrane current and potential. This is especially significant for grating orientation both because a tuning curve model failed to perform better than chance and because prior knowledge identifies thalamic populations as essential to orientation tuning V1^26–28^, thus evincing population decoding, rather than single-neuron decoding. To understand the importance of an ODE perspective, we also tested a Maximum Likelihood Stimulus decoder on a derivative-free state-space representation of neural time series; it failed to match the performance of dynamical discrimination. Additional scrutiny found evidence for task-positive and task-negative dynamical regimes, both in patterns of reliability for deflection responses and linear-stability analysis of the ODE models, thus demonstrating the value of ODEs as compact representations of neural time-series for testing the attractor network paradigm. We validated dynamical discrimination in increasingly complex neuron models driven by Lorenz dynamics to understand the sources of error and best-possible performance.

Ultimately, dynamical discrimination connects model discovery^25,29^ to latent variable discovery^11,30,31^, and exemplifies machine learning to test scientific hypotheses because its level of performance depends on the extent to which attractor networks underlie responses to drifting grating stimuli. Thus, it enables new methods of quantifying attractor network principles in the primary visual cortex^16,17^ and single-neuron intracellular recordings. More fundamentally, dynamical discrimination establishes proof of principle that intracellular recordings have hitherto unrealized potential as tools to investigate population neural code.

## Main text

### Dimensionality expansion captures dynamically rich neural trajectories from single neurons

Synaptically driven transmembrane electrotonic fluctuations contain rich information about network activity (Fig. 1c) but it is not clear how to get that information. If behavioral responses to stimuli are consistent, then fluctuations of neural activity following stimulus presentation should also be consistent at some level of abstraction. The abstraction proposed by the attractor network computational paradigm^12–15^ are attracting sets. The concept of tuning curves presumes that neural responses are stereotyped according to a single summary value, such as the mean spike rate or mean deflection, thus removing the information contained in fluctuations.

**Figure 2:**
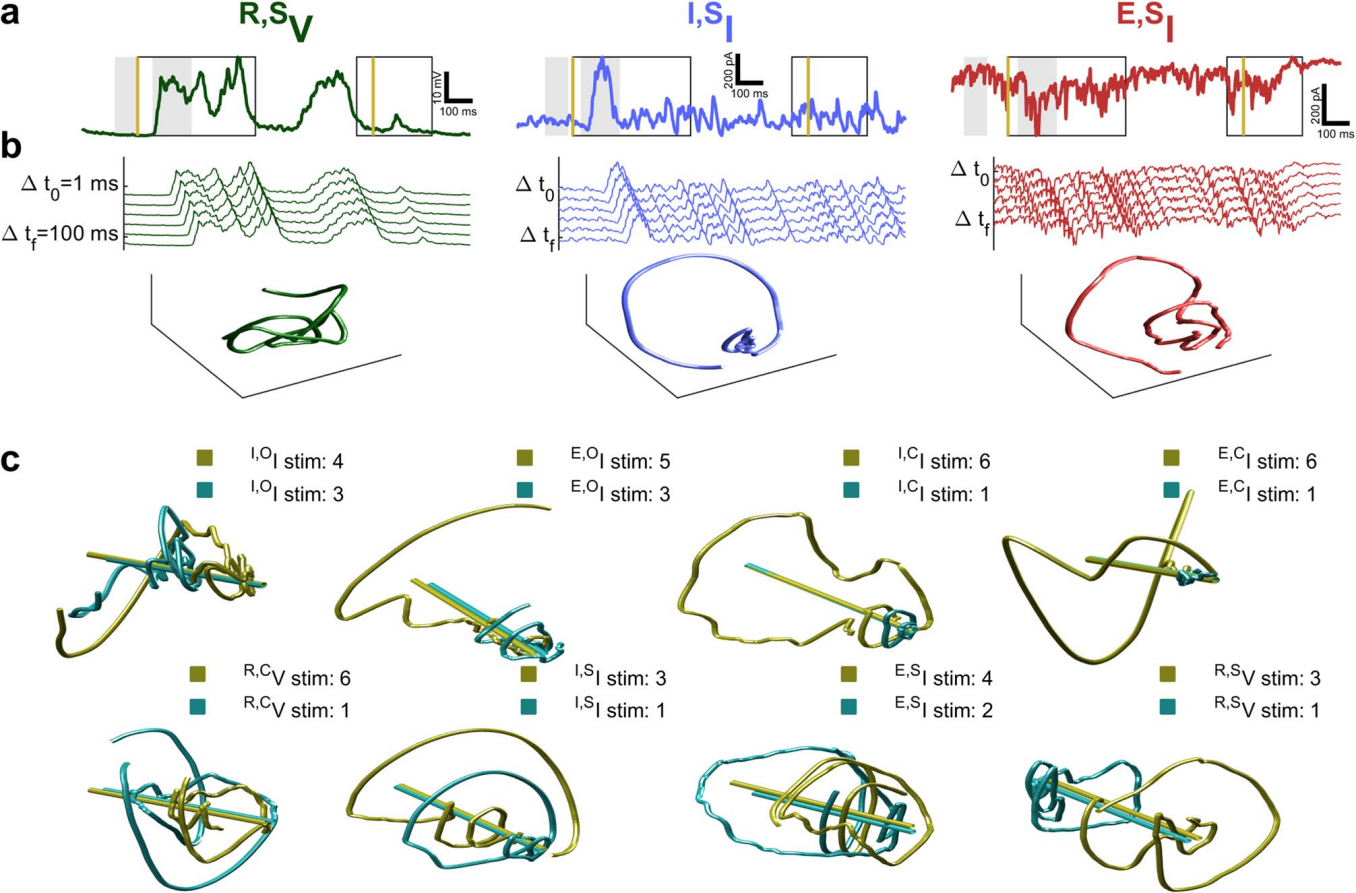
Time-delay embedding of intracellular recordings reveals varied dynamical trajectories and equations are fitted to them. (**a**) Excitatory (red) and inhibitory (blue) transmembrane current, and potential (green). Gold bars: stimulus on/off times. Outlined boxes: periods defining on response (early box) and off response (late box). Stimulus on time through the end of the off response defines the full response. Gray: periods defining deflection as the difference between early and late period means. (**b**) Left column: Time-delay embeddings illustrated with every 20th one-millisecond delay of recordings from panel a. Right column: Neural trajectories visualized after singular value decomposition of 100 one-millisecond delays. (**c**) Two trajectories coinciding with the most (gold) and least (aqua) preferred stimuli (largest/smallest mean deflection respectively) for one cell from eight recording categories. Fig. S10b succinctly characterizes all trajectories. Central axes (gold/aqua bars) are parallel. Trajectory characteristics include: axial displacement of densest regions (see ^***R,S***^***V***, ^***I,S***^***I***, ^***I,O***^***I***), opposed directions of divergence (^***R,S***^***V***), and nesting (^***I,O***^***I***, ^***E,O***^***I***, ^***I,C***^***I***, ^***E,C***^***I***, ^***I,S***^***I***). Most cell’s trajectories occupy conic regions (see Fig. S10), but some (^***R,C***^***V***, ^***E,S***^***I***, ^***R,S***^***V***) are cylindrical or spheroid when plotted together.

Recordings were placed into 10 categories according to signal type and characteristics of drifting grating stimulus (Fig. 1c), because it is important to demonstrate how our analyses perform in various conditions. Elucidating the biological impact of altering drifting grating parameters and contrasting them between signal types is beyond the scope of this paper. This paper is focused on demonstrating that more stimulus information arrives at the soma than tuning curves would suggest, and that this information evinces population dynamics. For details of drifting grating stimulus see the visual stimulation section of experimental methods. Types of recording and stimulus characteristic are denoted with left-superscripts above ***I*** for voltage-clamp recordings and ***V*** for current-clamp recordings (Fig. 2a). The two categories ^***I,O***^***I***, and ^***E,O***^***I*** feature transmembrane current recordings of synaptic inhibition (^***I***^) and excitation(^***E***^) respectively, and drifting grating orientation (^***O***^) was varied. The two categories ^***I,S***^***I***, and ^***E,S***^***I*** are the same, but size (^***S***^) was varied. For ^***I,C***^***I***, and ^***E,C***^***I***, contrast (^***C***^) was varied. For the categories ^***R,S***^***V***, and ^***K,S***^***V*** the recording apparatus was in current clamp mode and size was varied and spikes were removed (^***R***^) or kept (^***K***^), respectively. For two more membrane potential categories contrast was varied ^***R,C***^***V*** and ^***K,C***^***V***. Figures show only data with spikes removed because of minimal difference in outcome. There are 20–121 recordings for each cell (median is 68) with 3–21 examples of each stimulus (median is 11).

To employ attractor networks in intracellular recordings from mouse primary visual cortex responding to visual stimulation, we used time-delay dimensionality expansion^22,23^ of 500 ms recording snippets to project transmembrane current and potential fluctuations onto intermediate dimension neuronal trajectories (Fig. 2b). The observed trajectories form oscillations confined to conical, cylindrical, or spherical regions (Fig. 2c and supplemental Fig. S10). Trajectories appear limited to regions of phase space according to recording context (Fig. 2c and supplemental Fig. S10). Patterns include nesting conic trajectories inside one-another, and displacement of oscillation centers. As evinced by Maximum Likelihood Estimation (MLE) (see analysis methods: maximum likelihood estimation), trajectories were distinctive but the simple difference formula effect size for comparison with deflection-based discrimination according to a one-tailed Wilcoxon sign-rank test was r_SDF_ = 0.1351, which has a *p*-value of *p* = 0.263 (no difference, see supplemental S4). The MLE method we used computes the probability of a stimulus condition given the state-space occupied by a trajectory. Therefore, we eliminated the simpler hypothesis that projecting to a higher dimension state-space alone without dynamical systems characterization is sufficient for stimulus classification.

### Dynamical discrimination allows more precise stimulus discrimination than tuning curve methods

**Figure 3:**
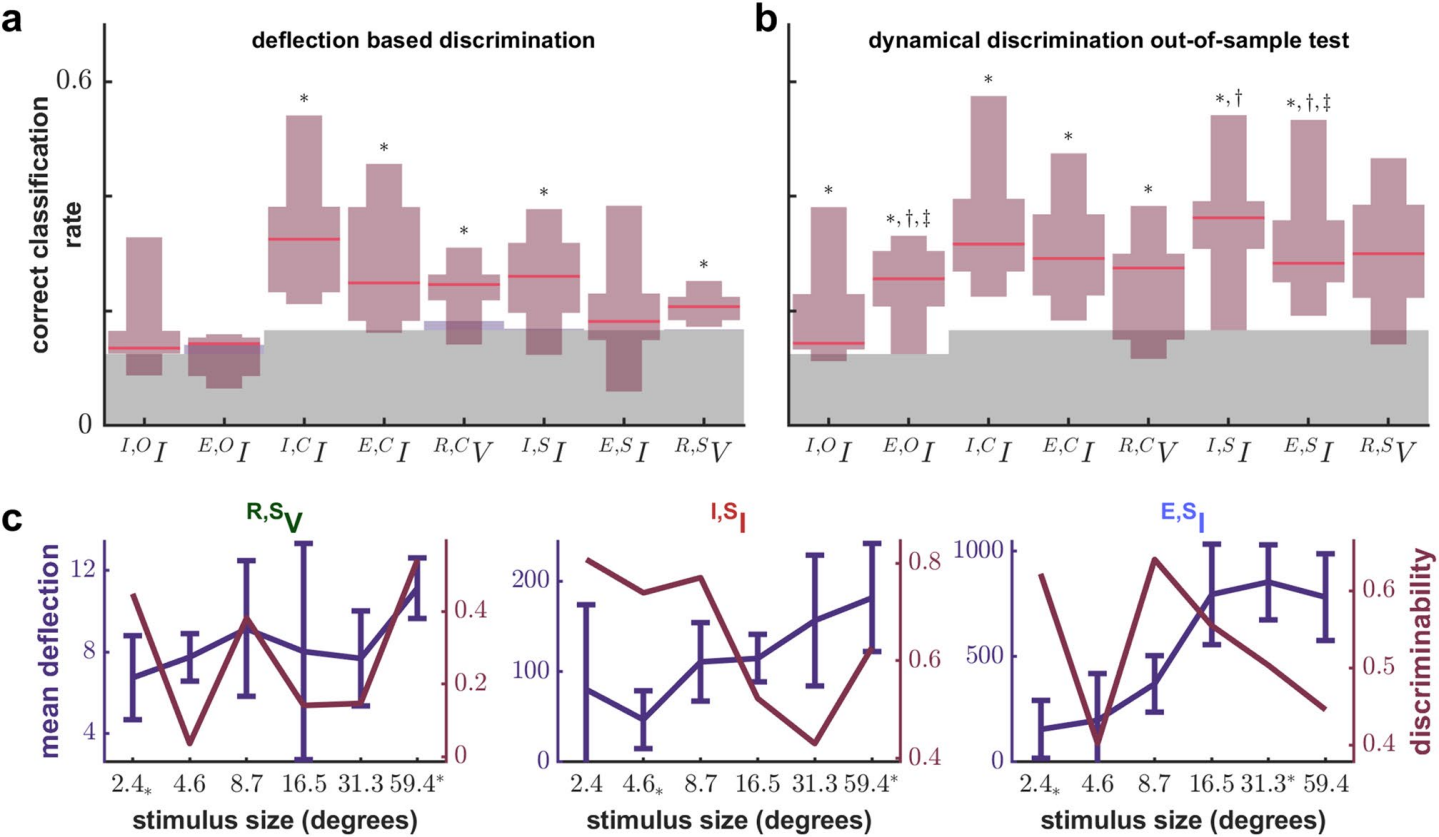
Discrimination performance of multiple methods across categories and compared with deflection for individual cells. (**a**) Red shading shows box and whisker plots of correct classification rates (CCR) based on deflection, with red lines indicating medians. Gray shading shows chance level. Columns separate all data by categories containing the same recording type and stimulus characteristic. * indicates significantly greater than chance performance in that category. Discriminating orientation and size (^***E,S***^***I***) fails with deflection. Barely visible above the gray region in some data groups is a lavender shading that indicates intrinsic overfitting tendency as measured with random surrogate testing. Overfitting is negligible for deflection-based discrimination. (**b**) Dynamical discrimination retains superiority after out-of-sample testing which eliminates the effect of any intrinsic overfitting tendency (which is shown in Fig. S2a). Distinguishability from chance, deflection, and best-fit results are indicated with *, †, and ‡ respectively. Orientation and size (^***I,S***^***I***, ^***E,S***^***I***) can be discriminated better than with deflection. (**c**) Average deflection (purple, error bars indicate standard deviation) and discriminability (dark red, F_1_ score of dynamical discrimination) as a function of drifting grating property. The least and most preferred stimuli are indicated with * and * respectively. Left: Membrane potential with spikes removed (mV), Center: inhibitory transmembrane current (pA). Right excitatory transmembrane current (pA).

We draw direct comparison with tuning curve methods which are a venerable way to characterize the impact of drifting grating stimuli on intracellular recordings. When changing a single parameter of a simple visual stimulus (e.g. changing size, contrast, or orientation of a drifting grating) the deflection from baseline of transmembrane current or potential (see Fig. 2a) or firing rate of a neuron will smoothly increase or decrease, but this is only true for cross-trial averages. The plot of cross-trial average deflection or firing rate for different stimuli is called a tuning curve. We decoded stimulus from deflections by dividing the range of possible deflections into smaller intervals (see analysis methods: deflection-based decoding). Each interval corresponds to one best-guess stimulus feature. The most and least preferred stimuli for a neuron are defined as those which evoke the largest or smallest (respectively) deflections on average for that neuron and are usually very dissimilar. When decoding only the most and least preferred stimuli, tuning curve methods excel^32^. We find correct classification rates (CCR) often exceed 0.9 (see supplemental Fig. S1 and Table S2). However, when decoding small changes in stimuli tuning curve methods are imprecise. Deflection-based discrimination failed to perform better than chance in for ^***I,O***^***I***, ^***E,O***^***I*** and ^***E,S***^***I***. However, it was better than chance overall with a 0.2281 median CCR (CCR_med_) and was distinguishable from chance for the remaining categories. This is summarized in Fig. 3a and Table 1. Sample tuning curves for size are plotted in Fig. 3c along with error bars that demonstrate the challenges for deflection-based discrimination. All tuning curves are provided in the supplemental S7 (Figs. S13–S18).

**Table 1:** Summary of results for discrimination methods presented in main text figures.

Algorithm and data groups	Median CCR	One-tailed Wilcoxon signed rank test that CCR is greater than that of…			
		Deflection-based discrimination		Chance	
		r_sdf_	*p*-value	r_sdf_	*p*-value
**All data groups pooled**					
Dynamical discrimination	0.2875	0.2022	3.60E−08	0.2385	5.49E−27
Deflection-based discrimination	0.2281	–	–	0.2218	5.85E−14
Dynamically stable state discrimination	0.2833	0.1912	2.73E−06	0.2377	8.28E−24
^**I,O**^**I**					
Dynamical discrimination	0.1437	0.1905	0.1875	0.2429	0.03125
Deflection-based discrimination	0.1353	–	–	0.1619	0.1094
Dynamically stable state discrimination	0.1375	0.1333	0.5313	0.2143	0.08594
^**E,O**^**I**					
Dynamical discrimination	0.2562	0.2571	0.01563	0.2	0.01563
Deflection-based discrimination	0.1431	–	–	0.1429	0.4688
Dynamically stable state discrimination	0.2062	0.2571	0.01563	0.1905	0.03125
^**I,C**^**I**					
Dynamical discrimination	0.3167	0.1753	0.1174	0.2581	3.05E−05
Deflection-based discrimination	0.3253	–	–	0.2581	3.05E−05
Dynamically stable state discrimination	0.35	0.1989	0.03296	0.2581	3.05E−05
^**E,C**^**I**					
Dynamical discrimination	0.2917	0.1591	0.2271	0.2581	3.05E−05
Deflection-based discrimination	0.249	–	–	0.2559	6.10E−05
Dynamically stable state discrimination	0.3167	0.1914	0.0535	0.2581	3.05E−05
^**R,C**^**V**					
Dynamical discrimination	0.275	0.1571	0.3125	0.2238	0.02246
Deflection-based discrimination	0.2463	–	–	0.2524	2.93E−03
Dynamically stable state discrimination	0.2042	0.08571	0.8389	0.2143	0.04492
^**I,S**^**I**					
Dynamical discrimination	0.3625	0.2372	3.36E−04	0.2297	7.63E−06
Deflection-based discrimination	0.2605	–	–	0.2387	2.67E−04
Dynamically stable state discrimination	0.3125	0.2252	1.68E−03	0.2568	3.81E−06
^**E,S**^**I**					
Dynamical discrimination	0.2833	0.2417	1.64E−04	0.2568	3.81E−06
Deflection-based discrimination	0.1819	–	–	0.1727	0.1061
Dynamically stable state discrimination	0.2667	0.2222	2.35E−03	0.2282	1.53E−05
^**R,S**^**V**					
Dynamical discrimination	0.3	0.2364	0.09375	0.2545	0.0625
Deflection-based discrimination	0.2077	–	–	0.2727	0.03125
Dynamically stable state discrimination	0.2	0.2182	0.1563	0.2727	0.03125

This table supports the claims and figures about dynamical discrimination (Fig. 3b), deflection-based discrimination (Fig. 3a), and dynamically stable state discrimination (Fig. 5e). Comparative algorithm performance is broken down by data category. Rows are grouped by data category as named in the first column. The next column lists algorithm names. The next column gives the median correct classification rate (CCR). The next four columns are in two groups. The columns within each group show either the effect size (r_sdf_) or the p-value for a one-tailed Wilcoxon signed rank test of the hypothesis that CCR was greater for the algorithm named on the row than either deflection-based discrimination or chance (which was 1/8 for orientation and 1/6 for size or contrast).

**Figure 4:**
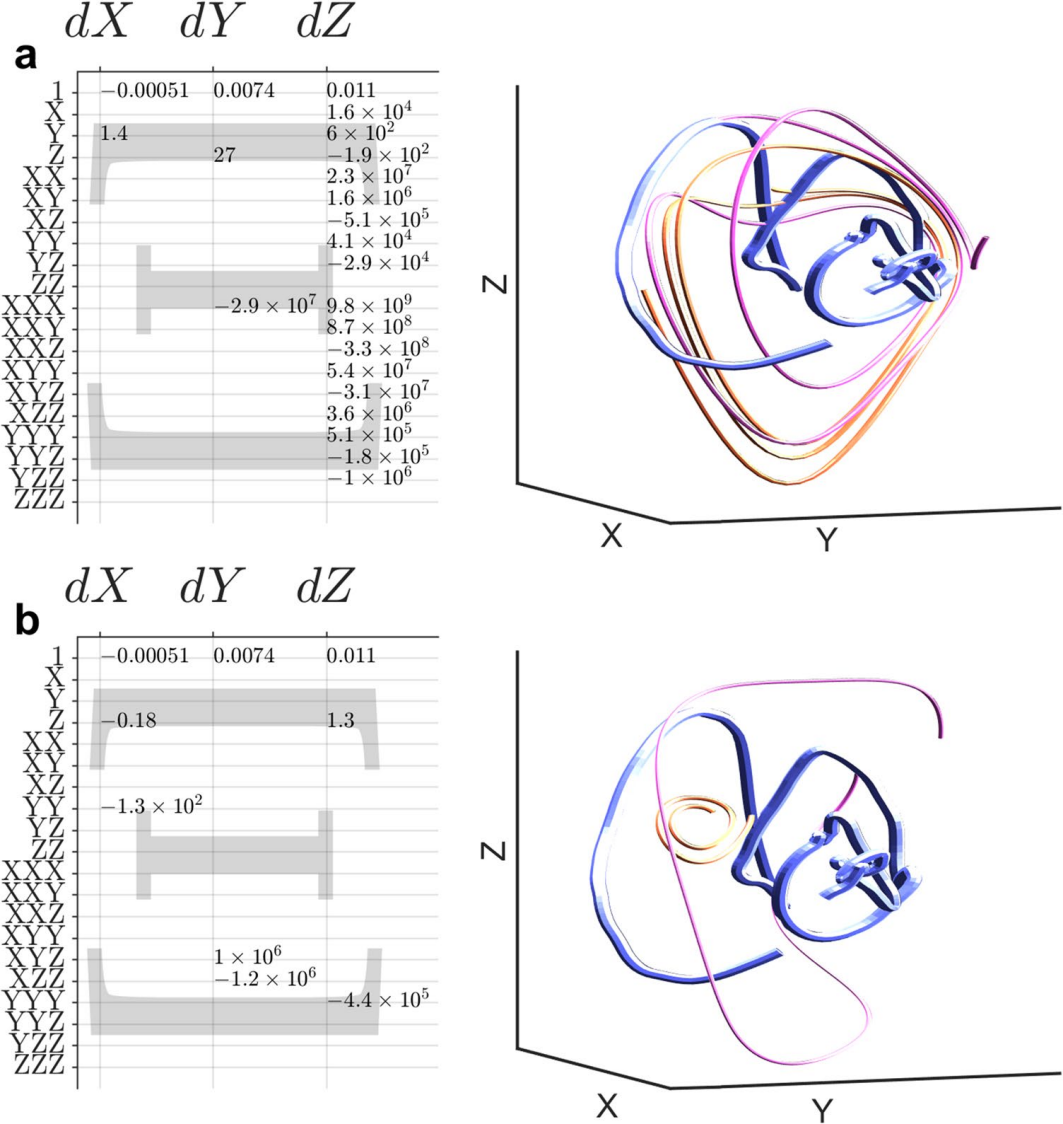
Time-delay embedding of intracellular recordings reveals varied dynamical trajectories and equations are fitted to them. (**a**) A Ξ matrix optimal for trajectory modeling (best-fit Ξ). Left: Ξ coefficients. Right: A neural trajectory (blue) and reconstructions (magenta/yellow). (**b**) This Ξ is optimized for stimulus discrimination (dynamical discrimination Ξ). Same plotting style as a.

The impact of changes in stimulation were more sensitively quantified by examining the coefficient matrices, Ξ, of polynomial Ordinary Differential Equations (ODEs) fitted to single-neuron trajectories (see Fig. 2b,c) by an augmented version of Sparse Identification of Nonlinear Dynamics (SINDy)^25^ (see analysis methods: genetic algorithm modification of SINDy, Fig. 1d). A genetic algorithm selected binary template matrices ^B^Ξ, that marked the location of nonzero coefficients in Ξ, numerosity varied from 7–20 (median is 12). We developed two genetic algorithms, identical except for their utility functions which judge the fitness of each ^B^Ξ. In the genetic algorithm version we called “dynamical discrimination”, stimulus classification (decoding) ability is emphasized (see analysis methods: classification objective function for genetic algorithm). We train MATLAB’s random forest classifiers at each generation and use the F_1_ score as fitness. Dynamical discrimination is more consistent with important prior work^22,33^, but less intuitive and potentially less insightful than the second version. The second version of the genetic algorithm, we called “best-fit Ξ based discrimination”, replaced classification ability with the goodness of fit (see analysis methods: goodness of fit objective function for genetic algorithm). For both dynamical discrimination and best-fit Ξ based discrimination the ultimate classification is made by an ensemble of 45 random forest classifiers, each based on one of the 45 highest utility ^B^Ξ matrices (see analysis methods: ensemble classification and out-of-sample generalization). Overfitting was directly measured and was negligible for all methods except dynamical discrimination (see Fig. 3a, S2) where it is controlled for in final results (Fig. 3b) by using additional 20-fold hold-one-out out-of-sample generalization. Details of our algorithm, stability analysis of ODEs, and hyperparameter optimization, and more is in supplemental S2. The ODEs usually captured the central axis and direction of divergence, though Ξ used for best-fit Ξ based discrimination were less sparse and better than Ξ used for dynamical discrimination at resembling original trajectories (Fig. 4a,b).

Dynamical discrimination and best-fit Ξ based discrimination both outperformed deflection-based discrimination and are reported in Table 1 and Fig. 3b, S2b. No method outperformed dynamical discrimination in any category, and it was distinguishable from chance in all categories except ^***R,S***^***V*** with CCR_med_ = 0.2875 overall, a 26% overall improvement over deflection-based discrimination. Both dynamical discrimination and best-fit Ξ based discrimination were distinguishable from deflection-based discrimination for the categories ^***E,O***^***I***, ^***I,S***^***I*** and ^***E,S***^***I***. Deflection-based discrimination performed poorly in these categories with dynamical discrimination showing an average 58% improvement in CCR_med_ across these categories. To understand the differences between dynamical discrimination and deflection-based discrimination we plot the F_1_ score (discriminability) for each of the six drifting grating sizes shown for ^***R,S***^***V***, ^***I,S***^***I*** and ^***E,S***^***I***. Performance with different dimensions, types of ODEs, and different epochs of visual stimulation are included in supplemental S2. For the membrane potential categories ^***R,C***^***V***, and ^***R,S***^***V***, median performance was better for dynamical discrimination but there were too few data points and too much variability to draw any conclusions.

### Dynamical discrimination performance varies by data source, emphasizing differences between single neuron and population encoding and decoding

One of our key points, that neurons receive more fine-grained information than they are able to pass on through action potentials is evinced by scrutinizing discrimination of orientation. Prior research has found orientation tuning in excitatory thalamic input synapses to mouse V1, layer 4^26^. Observing a large set of elements with unique tuning (such as these synapses) is one basis for understanding population code^34^. However, it is a step removed because we record from neurons in layer 2/3. If dynamical discrimination was limited to the same information neurons use to compute deflection it would have the same limitations. Instead there is a 79% improvement in CCR_med_ for dynamical discrimination compared to deflection-based discrimination for ^***E,O***^***I***. Importantly there is much less improvement for ^***I,O***^***I*** even though ^***E,O***^***I*** and ^***I,O***^***I*** performed similarly for deflection-based discrimination. Only excitatory input has been identified as having orientation tuning. If dynamical discrimination was a needlessly complicated method redundant to deflection then we would expect to preserve the relative performance between ^***E,O***^***I*** and ^***I,O***^***I***. We do not see this for orientation tuning, indicating that dynamical discrimination is sensitive to the differences in upstream populations whereas deflection-based discrimination is not.

Tuning curves are different based on the different parameters being varied when generating them. The shape of the curves themselves may explain why deflection-based discrimination fails^35^ and helps to emphasize that dynamical discrimination and deflection-based discrimination are not based on the same information. When varying orientation, tuning curves are classically modeled as Gaussian. A Gaussian is not an invertible function. There are at least two possible orientations for every cross-trial averaged level of deflection, except for the most preferred orientation (peak of the Gaussian). When varying drifting grating contrast or size, our tuning curves were frequently found to be “ramp functions” (roughly linear) or sigmoidal (a flat-S-shaped curve). These are plotted in supplemental S7 (Figs. S13–S18). Sigmoid functions are roughly flat near both the most and least preferred stimuli with a relatively rapid change for some intermediate stimuli. The flat tails of sigmoid functions are difficult to invert if there is any variability, while ramp functions are the easiest to invert. Thus, we expect less room for improvement over a ramp-function tuning-curve decoding than for a sigmoid-function tuning-curve decoding. For transmembrane current we find little difference in the shape of size and contrast tuning curves. Thus, if dynamical discrimination was based on the same dynamics behind tuning curves, we would expect ^***I,S***^***I***, and ^***E,S***^***I*** to show the same improvement as ^***I,C***^***I***, and ^***E,C***^***I***. Yet dynamical discrimination showed a much larger improvement for ^***I,S***^***I***, and ^***E,S***^***I*** than for ^***I,C***^***I***, and ^***E,C***^***I***. The correct classification rates of dynamical discrimination for groups ^***I,S***^***I***, and ^***E,S***^***I*** showed a 47.5% improvement over deflection-based discrimination. While there were no significant differences for ^***I,C***^***I***, and ^***E,C***^***I***, this is not the trend we would expect if dynamical discrimination was based on the same dynamics behind tuning curves.

### Dynamical discrimination is linked to distinct and reliable presynaptic population dynamics

**Figure 5:**
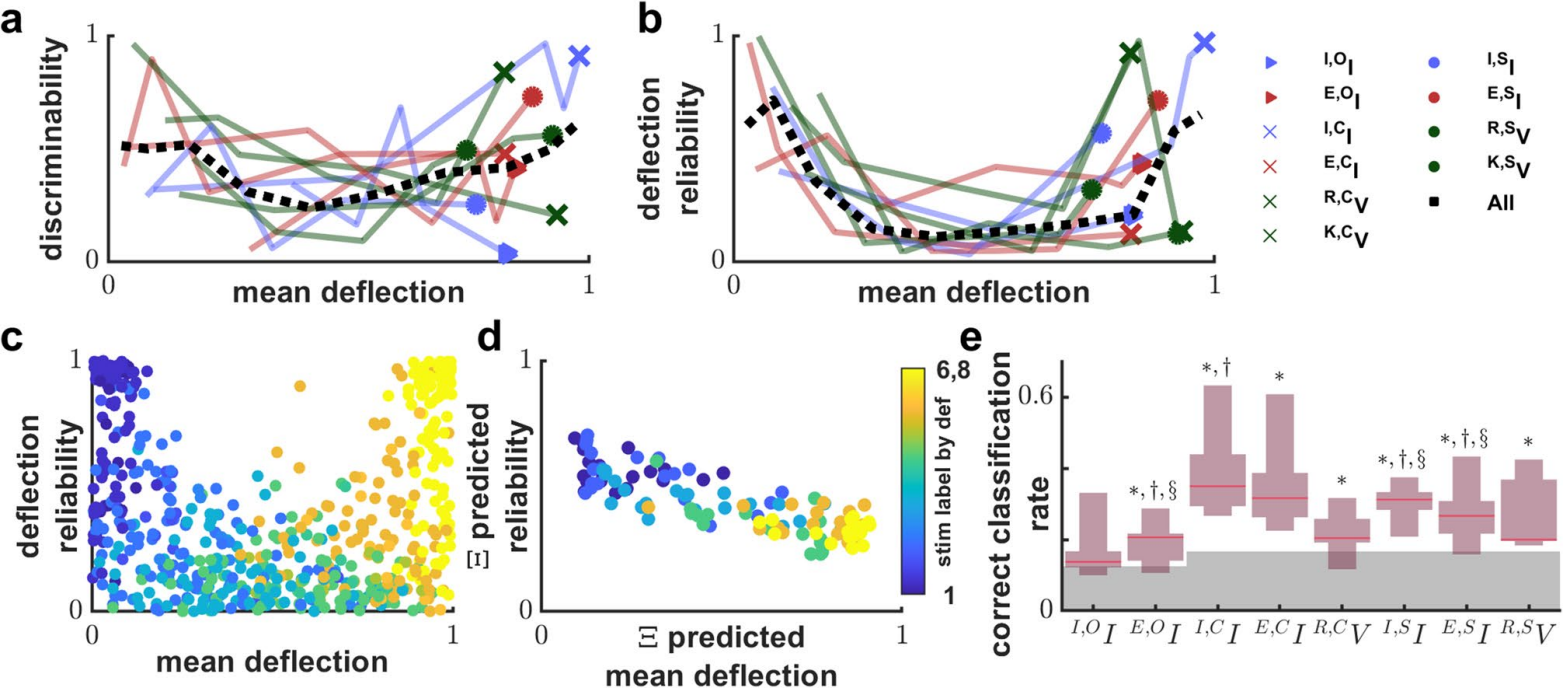
Single trial discriminability depends on dynamical states associated with stimulus selectivity. (**a**) The U shape of normalized discriminability vs normalized mean deflection indicates least and most preferred stimuli are more discriminable. For each decile of normalized mean evoked deflection, its median is plotted against the median normalized discriminability (F_1_ score of dynamical discrimination). The legend right of panel b maps color and end-point marker to data categories. Dashed line indicates pooling of all categories. (**b**) Deflection reliability (inverse coefficient of variation) shows a more prominent U trend (same style as a). (**c**) Stimuli are roughly separable on a scatter plot of reliability and mean deflection across all data points. Stimuli are re-labeled and colored by their rank of average evoked deflection (color bar is right of panel d) (**d**) A plot of trial-by-trial predictions of cross-trial means. Predictions are from random forest regressors trained on Ξ matrices re-appropriated from dynamical discrimination. What separability remains now extends to a trial-by-trial basis. (**e**) Same as in Fig. 3b except now the context (original stimulus label) is inferred using only the predicted state variables (reliability and deflection) from panel d. Distinguishability from chance is denoted with *, from deflection with †, and § indicates significantly worse performance than Fig. 3b (the reverse was never true). The key results from Fig. 3b are reproduced despite stripping information down to just two understandable variables: reliability and deflection. Furthermore ^**I,C**^**I** was distinguishable from deflection which did not occur for Fig. 3b.

Having clarified the extent to which dynamical discrimination and deflection-based discrimination are based on different information, we then identified what they have in common. To do that we compared the discriminability of stimuli by dynamical deflection directly to deflection-based tuning curves and the reliability of evoked deflection responses. We found that the most and least preferred stimulus tends to be the most discriminable for every signal source and stimulus type (see the U-shape of Fig. 5a). We defined reliability as the mean deflection divided by the standard deviation and found that reliability was greater for deflections evoked by the most and least preferred stimuli as seen in the U-shape of Fig. 5b. Because deflections are smallest for the least preferred stimuli (though rarely with a mean near zero), this means that the standard deviation of deflections decreases even faster than the mean deflection for least preferred stimuli, but not intermediate stimuli. We needed to demonstrate that the U-shapes of Figs. 4b and 5b are not apophenia. We define the term “key-distance” as the ordinal-number distance to either the least preferred stimuli or the most (whichever is smallest, see analysis methods: tuning curve reliability). Then, to validate the U-shapes we calculated the correlation between key-distance and the F_1_ score of dynamical discrimination (Fig. 5a) to be 0.26 (Pearson correlation coefficient) with *p* = 5.04 × 10^–12^, and the correlation between key-distance and reliability (Fig. 5b) was 0.60 (Pearson correlation coefficient) with *p* value *p* = 3.45 × 10^–67^. Two factors are important to note. First, we know that population dynamics evoked by the most and least preferred stimulus are distinct from each other because the average deflection is so different (see also Fig. S1b). Second, because reliability is much higher for these most/least preferred stimuli we know they evoke more consistent population dynamics than the intermediate stimuli.

We consider two possible explanations for the U-shaped reliability pattern (Fig. 5b). The simplest explanation, the “random process explanation”, is that transmembrane current or potential has relatively bigger fluctuations or has more noise about the cross-trial average for intermediate stimuli. The other explanation, the “dynamical regimes explanation”, is that the least and most preferred stimuli each consistently evoke a different highly stereotyped response and that intermediate stimuli inconsistently evoke one or the other. To test whether fluctuation size or noise is responsible we defined fluctuation size as the coefficient of variation for single recordings and defined noise as the mean of fractional response residuals (see analysis methods: tuning curve reliability). Fluctuation size and noise both simply decreased with increasing average deflection and did not exhibit a U-shaped trend, suggesting neither are responsible for increased response reliability, alone or in combination (see supplemental S3, Fig. S9).

Having eliminated the random process explanation, we investigated the dynamical regimes explanation. We devised two partial tests. The first test employs linear stability analysis to find evidence for bifurcations in the ODE models directly, however they are crude approximations. The second test assumes that if there are two distinct regimes and reliability and deflection are latent variables identifying the regime then dynamical discrimination Ξ matrices should be able to predict reliability and deflection on a single trial basis, and we should be able to discriminate stimuli using these predictions without a large performance decrease. Linear stability analysis^19^ defines dynamical systems regimes through analytical properties of real-valued zero-gradient solutions to the ODE, called “fixed-points” (see analysis methods: Linear stability analysis). We found that the most preferred stimulus stood out. Stimuli dissimilar to the preferred stimuli produced a dissimilar number of fixed-points (see supplemental S3, Fig. S8). Additionally, the fraction of net-convergent fixed-points inversely correlated with dissimilarity to the preferred stimulus (Fig. S8). The fact that the preferred stimuli tend to have the most or the fewest fixed points provides some confidence for proceeding to the next test. We re-analyzed ensembles of Ξ matrices with random forest regression to estimate reliability and average deflection (Fig. 5d) then fed the estimates to a third random forest classifier to predict the stimulus labels (see Fig. 5e). While there was a small 1.6% decrease in performance overall with CCR_med_ = 0.283. The simple difference formula effect size for comparison with dynamical discrimination was r_SDF_ = 0.141, which has a Wilcoxon sign-rank p-value of *p* = 0.0061. This was the second-best method of classification, retaining most of the key results with better than chance classification of ^***E,O***^***I*** and better than deflection performance in ^***E,O***^***I***, ^***I,S***^***I***, ^***E,S***^***I***, and even ^***I,C***^***I*** (see supplemental Table S1). The failure of the random process explanation and qualified success of a dynamical regimes explanation for the U-shaped trends in Fig. 5a,b reveals some common ground between a dynamical systems approach and a deflection-based approach to analyzing the visual system. Deflections may be more reliable, and dynamical discrimination may perform better for the least/most preferred stimulus because repeated presentations of the least and most preferred stimuli are more likely evoke dynamics along the same attracting set than presentations of intermediate stimuli.

### Dynamical discrimination is corroborated by biologically plausible modeling

**Figure 6:**
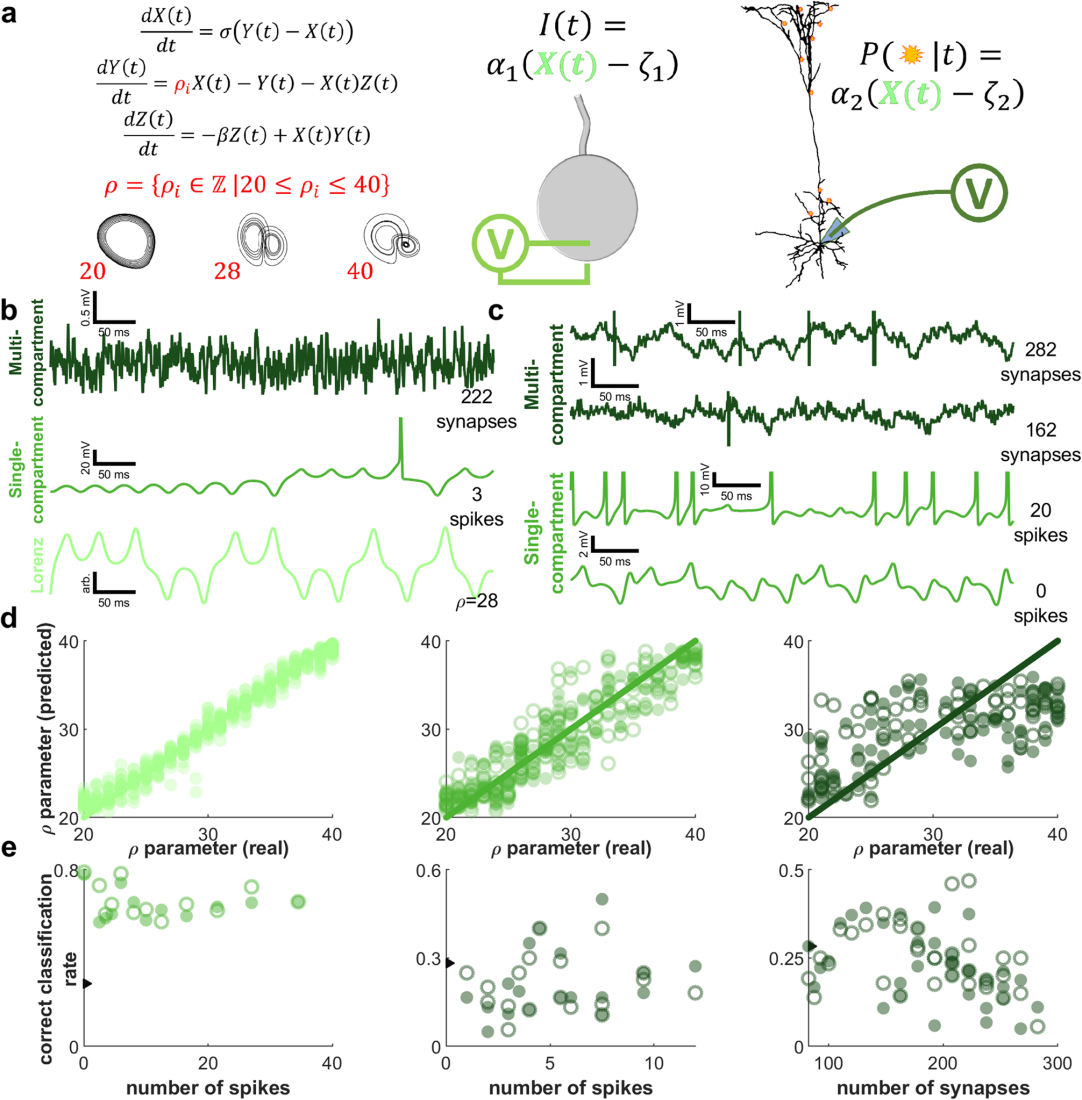
Modeling tests confounding factors for dynamical discrimination and matches experimental results. (**a**) Our modeling paradigm, illustrated. Left: We tested regressing Ξ onto integer values of the Lorenz system parameter ρ from 20 to 40 (spanning a Hopf bifurcation and chaos). Center: a single-compartment neuron (X as current injection). Right: a multi-compartment model with dendritic spines (NEURON shape plot, X governs synapse transmission probability). (**b**) Simulated membrane potential. Top: multi-compartment model (dark green). Middle: single-compartment neuron (medium green). Bottom: X from Lorenz system (light green). (**c**) Examples of possible confounds. Top: two traces showing differences associated with synapse numerosity. Bottom: two traces showing action potentials dominating Lorenz dynamics. (**d**) Regressions of Ξ onto ρ plotted against true ρ values. Left: Fitting Ξ to X performed well (light green). Center: Fitting Ξ to membrane potential of single-compartment neurons moderately reduced accuracy (medium green). Right: Multi-compartment models significantly degraded regressions (dark green). Performance is similar whether spikes are removed (filled) or not (open). (**e**) Correct classification rate (CCR) vs possible confounds. Ξ matrices trained for regression are reappropriated for classification and ρ is limited to^22,25,28,32,35,38^ (chance 1/6$\hspace{0.17em}\approx \hspace{0.17em}$0.167). Left: Dynamical discrimination is robust to spiking for single-compartment models (medium green). Center: Spiking also has limited impact for multi-compartment models (dark green). Right: Synapse numerosity (model complexity) is impactful (dark green). Arrow ticks indicating median CCR for data in Fig. 3b evince modeling and experiment agreement but higher potential for dynamical discrimination with continuous dynamics.

Because dynamical discrimination is based on estimating the coefficients of ODEs, it is incumbent on us to test it with the coefficients of known ODEs. Our overall strategy, illustrated in Fig. 6, is to challenge dynamical discrimination by using resampling and neuron models to degrade data generated from a known system. Instead of using Ξ matrices to predict a label from a small set of possible labels, the genetic algorithm used regression to accurately estimate small changes to a parameter of the Lorenz system, ρ, as we varied it between the integers 20 to 40.

The Lorenz system is:

$$ \begin{aligned} \frac{dX}{{dt}} & = \sigma \left( {Y - X} \right) \\ \frac{dY}{{dt}} & = X\left( {\rho - Z} \right) - Y \\ \frac{dZ}{{dt}} & = XY - \beta Z \\ \end{aligned} $$

We chose ρ to include a Hopf bifurcation at $\rho =\frac{\sigma (\sigma +\beta +3)}{(\sigma -\beta -1)}\hspace{0.17em}\approx \hspace{0.17em}24.7$ and to explore chaotic regimes. We used the X dimension of the Lorenz system but resampled each trial $X^{{\prime}}(t)=X(t/\tau )$ where τ is chosen such that 90% of the signal power was in Fourier modes below 300 Hz for each trial (see analysis methods: single neuron modeling, Fig. 6b). As seen in Fig. 6d our approach excels at predicting the value of ρ from a sample of time series data. Median absolute percent error between the predicted value of ρ and the real value has a median of 1.79% with a 25th percentile, P_25_ = 0.87% and a 75th percentile P_75_ = 3.46%, verifying the identification of ρ for nearly ideal conditions. Integration of the model ODEs reproduces the Lorenz trajectories much better than experimental neural trajectories, as shown in supplemental S6 (Fig. S12).

We followed up by distorting $X^{{\prime}}(t)$ with successively more complex neuron-like transformation. We fed the resampled X dimension from the Lorenz equation into a single-compartment neuron model by shifting and rescaling to be consistent with injected current (no synapses) on the order of nano amps $I(t)={\alpha }_{1}(X^{{\prime}}(t)-{\zeta }_{1})$ where ζ_1_ and α_1_ are chosen to produce the desired amount of spiking (see analysis methods: single neuron modeling, Figs. 5c, 6b). Median error was 5.45% with P_25_ = 2.52% and P_75_ = 9.18% (Fig. 6d). All values reported in the text come from data where spikes were removed (median filter with a five millisecond window) which made little difference for any model. The still good performance shows that transformation by membrane dynamics does little to interfere with our methods.

In a biologically plausible attractor network, the dynamics govern a point process with inputs to a neuron arriving as discrete events. Therefore we fed the single resampled dimension, X from the Lorenz equation into a morphologically complex multi-compartment neuron model^36^ by shifting and rescaling it to be consistent with the instantaneous event probability of an inhomogeneous Poisson process $P(t)={\alpha }_{2}(X^{{\prime}}(t)-{\zeta }_{2})$ where ζ_2_ and α_2_ are chosen to produce the desired amount of spiking (see analysis methods: single neuron modeling, Fig. 6b). Models with fewer (excitatory only) synapses results in a membrane potential time series that has large and sporadic synaptic potentials (impulses). Initially adding more synapses produces a signal that better visually approximates the input time series, but eventually increases dynamical complexity and spiking unpredictability (Fig. 6c). Integration of the model ODEs varied greatly in how well they reproduce trajectories from neurons driven by Lorenz-governed synaptic input as shown in supplemental S6 (Fig. S12). This more complex type of model yielded a median error of 12.02% with P_25_ = 8.11% and P_75_ = 19.99% (Fig. 6d). Thus, synaptic type distortions have degraded ODE fitting approaches but not left them unworkable.

To directly compare with experimental dynamical discrimination results we treated the ρ values^22,25,28,31,34,37^ as if they were distinct stimuli labeled one through six and re-analyzed the corresponding Ξ matrices by training classifiers to predict the labels. Chance level is 1/6 ≈ 0.167. We also categorize model trials according to the number of spikes and synapses because they may be confounding factors. Single-compartment neuron data formed 12 categories according to similar spiking levels (see analysis methods: single neuron modeling). Multi-compartment models yielded 18 spiking categories (limited to models with 245–290 synapses for Fig. 6e) and 35 synapse numerosity categories (limited to trials with four or fewer spikes for Fig. 6e). The impact of spike rate and synapse numerosity is shown in Fig. 6e where an arrow indicates overall CCR_med_ = 0.2875 for dynamical discrimination in-vivo (for comparison). For single-compartment data (spikes removed) CCR_med_ ranged from 0.563 to 0.778 and was indifferent to the level of spiking. For the multi-compartment spiking categories, CCR_med_ was low and inconsistent ranging from 0.05 to 0.5, but also showed no association with spiking. However, synapse numerosity categories did show a trend; CCR_med_ values ranged between 0.05 and 0.393 peaking at around 162 synapses. For in-vivo dynamical discrimination, CCR_med_ ranged from 0.144 to 0.363. Thus we see our results for in-vivo data are near the ceiling for this implementation of dynamical discrimination and in line with expectations for synaptic impulses that are completely governed by a dynamical system being manipulated through a bifurcation and chaos.

## Discussion

This work presented compelling observations of the ability to access population dynamics through single intracellular recordings. First, membrane potential and transmembrane current recorded from neurons in mouse primary visual cortex underwent dimensionality expansion. This yielded neural activity trajectories which appear to be stimulus modulated. Second, motivated by attractor network principles^12–17^, ordinary differential equation models (Ξ matrices) were fitted to individual trajectories. Ξ matrices described each trajectory compactly enough to enable classifiers to decode the stimuli from limited neural data. Called dynamical discrimination, this algorithm more accurately predicted fine changes in orientation, contrast, and size of drifting gratings than predictions made from firing rate substitutes (deflection) and alternatives. Furthermore, only dynamical discrimination had the fidelity to confirm findings about orientation selectivity differences between excitatory and inhibitory synaptic mechanisms. None of these findings would be possible if the attractor network theory motivating dynamical discrimination was not applicable to V1 dynamics. Third, stimuli evoking extremes of average deflection also evoked the most reliable deflections but not the smallest fluctuations, or least noise. An explanation came by way of re-analysis of Ξ matrices which provided evidence for coherent and distinct dynamical regimes like task-positive and task-negative dynamical regimes^31,37^, which intermediate stimuli may alternately evoke. Lastly, modeling validated the level of accuracy. Dynamical discrimination excelled with continuous nonlinear transformations of underlying dynamics but transforming dynamics into a point process (like synaptic transmission) degraded performance to the experimentally observed levels. These four results show that: (i) Dynamical discrimination is a powerful method for general time series and trajectory classification with limited data. (ii) Attractor network principles can be applied to primary visual cortex and to single neuron recordings, and (iii) dimensionality expansion and dynamical discrimination lets researchers patch into upstream network by intracellularly recording from single neurons.

The general principle of dimensionality expansion is familiar to neuroscience (e.g. separating a signal into time-varying oscillatory modes) though attractor reconstruction is still emerging^23,33^. Time-delay embeddings (Fig. 2) may be rare in neuroscience but are trusted for time-series analysis^24^ and counterpoints the commonly used dimensionality reduction of population activity^38^. Dimensionality reduction to abstract spaces is used even with advanced population recording methods^39–41^, and methods for isolating and grouping single units by functional and anatomical relevance^36,42,43^. Thus dimensionality expansion on single neurons can offer comparable insights about attraction to neuronal manifolds^31^ but the grouping is naturally defined to be all presynaptic neurons^1,44,45^.

Researchers have tried many ways to map synaptic activity recorded at the soma to population events and dynamics^1,2,46–49^. Machine learning algorithms are sensible options but often don’t permit scientific inference beyond their predictions themselves^5,30^,. Hypothesis-dependent methods leverage computational assets to fit a model to data and exploit the model for a new purpose^25,29,50,51^. If the model is fitted poorly or not applicable the algorithm performs poorly, thereby testing the hypothesis motivating the choice of model. This is why ground breaking earlier works^22,23,25,52^ inspired us to develop dynamical discrimination.

For experimental data, dynamical discrimination had limited classification accuracy. However, this is expected and exceeds all compared methods. Modelling showed that synaptic transmissions completely governed by a simple dynamical system produced the same levels of classification accuracy, while displaying high accuracy in more ideal scenarios. Dynamical discrimination was further corroborated by recapitulating historical findings such as differential tuning among excitatory and inhibitory populations^21,53^. Furthermore, we gained new information regarding patterns of response reliability^37^. Performance may improve since this was a simplistic version of the algorithm. Classification algorithms work best (avoid overfitting) with both fewer original features and fewer model parameters than the number of examples used for training. Single cells yielded too few trials (median is 68) for deep learning sequence classification. Fitting ODE models reduced trajectories with thousands of time points to tens of coefficients (median is 12). Existing deep-learning approaches like LFADs^11^ are neither human readable nor sufficiently parsimonious for low-data multi-label classification, but are a promising route to great improvement. Thus, we conclude that limited accuracy is expected for this first version of dynamical discrimination and belies its other virtues as an analytical tool (e.g. it provides an ODE model).

This work advances physics and neuroscience in several ways. We must specify and isolate relevant parts of the brain before we can elucidate their interactions. The brain has natural partitions such as layers, nuclei, and cortical columns, but imaging fields and electrode arrays often overlap or partially cover them. We offer proof of concept for exploiting single neurons as deeply and naturally integrated sensors of population dynamics within an elementary natural brain partition and bottleneck: the neurons synapsing onto a neuron of interest. The fundamental concept is to apply dimensionality expansion to intracellular recordings, yielding trajectories akin to those from dimensionality reduction on hypothetical high-dimensional recordings of these populations. Excitatory thalamic projections bring orientation information to V1^26^, but tuning-curve based methods (deflection) missed fine distinctions while dynamical discrimination more fully utilized the afferent projecting population dynamics. Because this works on a trial-by-trial basis^48^ not an average over trials, we have a versatile tool for investigating neural representation. Dynamical discrimination’s power to identify latent factors^11,30,31^ (i.e. context) may advance traditional single-neuron topics like orientation tuning^27,28^ and interplay between excitatory and inhibitory populations^21,44^. The relation to population dynamics and to functional specificity and connectivity^43,51^ may be further clarified by combining our (or similar) methods with multiple simultaneous intracellular recording and/or concurrent electrical stimulation, or cell staining and tracing. An advance for machine learning is our incorporation of a hypothetical paradigm (attractor networks) into the core apparatus of a classifier. Consequently, dynamical discrimination connected stimulus tuning to attractor network principles. Because of these demonstrations, machine learning for science can escape the black-box, decades of single-neuron intracellular recordings can be re-analyzed for population insights, and the attractor network paradigm has come to primary visual cortex.

## Methods and materials

### Experimental methods

The experimental data was originally gathered to support another publication^21^. The methods are covered there and relevant details are repeated here^54^.

#### Statement of ethical approval

All procedures were approved by the University of California, Berkeley ACUC and were performed in accordance with relevant guidelines and regulations. Wild-type (C57;B6 × ICR white), emx1-IRES-Cre, and SOM-IRES-Cre mice were used. Mice of both sexes were used equally, and no differences were observed between sexes. For in vivo recordings mice were 5–14 weeks old.

#### Animals: surgery and electrophysiological recording

Mice were headplated under isoflurane (1.5–2%) anesthesia with a small stainless-steel plate, attached to the skull with Metabond. The skull was protected with cyanoacrylate glue and dental cement (Orthojet). 1–7 days post-surgery, Mice were habituated to run freely on a small, 6″ diameter rotating disc during head fixation. On the day of surgery mice were anesthetized with 1.5%–2% iso-flurane and a small craniotomy was made over V1 by removing the dental cement and slowly thinning the skull until it was trans­parent with a 0.25 mm carbide burr. A small stainless-steel needle (27G) was used to open a hole 150–500 um in diameter over V1 with no or minimal bleeding. The dura was always left intact. The craniotomy was covered with sterile saline and the animal was allowed to recover under fixation for 15–30 min prior to whole-cell recording. Animals typically began running on the treadmill immediately upon arousal, and either continuously or intermittently thereafter. Under these experimental conditions mouse move their eyes only infrequently, and most ocular deviations are too small to significantly impact neuronal responses^55^, and the pupil was not tracked.

#### Electrophysiology

Prior to intracellular experiments, a patch pipette filled with ACSF (in mM: NaCl 119, KCl 2.5, MgSO_4_ 1.3, NaH2PO_4_ 1.3, glucose 20, NaHCO_3_ 26, CaCl_2_ 2.5) was lowered slowly into the L2/3 under visual guidance (Leica MZ6 stereomicroscope). Using multiunit ac­tivity and the LFP as a guide, the visual receptive field of the corresponding location for subsequent whole cell recording was mapped via a hand-controlled small circle (5 degrees) of changing contrast on the visual stimulus monitor (more details below). This elec­trode was then removed, and patch pipettes were then inserted in same manner for intracellular recording containing: CsMeSO_4_ (for voltage clamp) or KGluconate (for current clamp) 135 mM, NaCl 8 mM, HEPES 10 mM, Na_3_GTP 0.3 mM, MgATP 4 mM, EGTA 0.3 mM, QX-314-Cl 5 mM (voltage clamp only), TEA-Cl 5 mM (voltage clamp only). Although the cells were patched with the blind approach, the conditions used have been reported to strongly bias recording to regular-spiking putative pyramidal cells^56^. Nevertheless, the data reported here is likely to come from a mix of cell types, dominated nevertheless by excitatory neurons, which make up the majority of L2/3 cells.

Under these conditions, in voltage clamp, the mean series resistance, prior to any compensation, was 18 ± 1 MΩ across the recording sessions, and fairly stable^21^. It is now well established that locomotion and/or brain state influence spontaneous activity and sensory responses in V1^57–60^, although the exact mechanisms underlying these changes remain a matter of debate^61–63^. Consistent with prior findings, during locomotion^64^, visually evoked E and I were significantly increased (E: not running: 70 ± 6 pC/s, running: 81 ± 8 pC/s, n = 39 cells, p < 0.005; I: not running: 114 ± 12 pC/s, running: 159 ± 20 pC/s, n = 39 cells, p < 0 0.005, Wilcoxon sign rank test^21^). Conversely, spontaneous excitation and inhibition, as well as the mean input conductance in the absence of a stimulus showed no significant change (E: *p* = 0.9; I: *p* = 0.4, input resistance: *p* = 0.93, n = 39 cells, Wilcoxon sign rank test^21^).

Both extracellular and intracellular experiments employed an Axopatch 200B amplifier. All data were acquired with custom software written in MATLAB using a National Instruments PCIe-6353 card. Glass pipettes (Sutter instruments) containing either a potassium based internal (for measurements of membrane potential and spiking) or cesium (with added QX-314-Cl, and tetraethy-lammoniaum-Cl) for voltage clamp recording, were used. Pipettes were pulled on a Sutter P1000 puller in a two stage pull to a long taper pipette of a resistance between 3 and 5 MOhm. To insert the electrode into the small craniotomy, the ACSF on the skull was removed and the craniotomy briefly dried with compressed air. The electrode was mounted on a Sutter MP285 manipulator, lowered until it nearly reached the brain surface, then the chamber formed by the headplate and cement was re-filled with ACSF, all under visual guidance. The pipette resistance was checked via an oscilloscope and a constant 5 mV voltage step in voltage clamp. High positive pressure (150 mbar) was applied to the pipette, and it was lowered until a brief and rapid increase in pipette resistance was observed, indicating contact with the dura. The pipette was zeroed to obtain an accurate measurement of recording depth, and then the pipette was advanced quickly through dura, and only pipettes that quickly returned to their baseline resistance were advanced further, otherwise they were exchanged for a fresh pipette and the process was repeated. Once inside the brain the pres­sure was quickly lowered to 10–30 mBar to search for L2/3 neurons via abrupt, ‘bounce’ like changes in pipette resistance indicating contact with a plasma membrane, using pulsatile steps of the manipulator (1–2 microns). Upon apparent contact, pipette pressure was released, and slight positive pressure was used to obtain a gigaohm seal. Pipette capacitance was then neutralized, and the membrane ruptured by brief suction pulses. Upon rupture the whole cell access was optimized by either slow negative or (more typi­cally) positive pressure and locked off. In the first 2–4 min the receptive field of the cell (either via membrane potential, spiking, or excitatory current, command potential = − 70 mV) was remapped in the same manner as above, to center the stimulus on the re­corded cell’s receptive field (almost always aligned with the previous measurement from extracellular recording). The orientation of the stimulus was also optimized for each cell. After spontaneous and evoked responses stabilized (typically 2–4 min) experiments were commenced. Membrane potential was obtained in voltage following mode (current clamp) with no current injection. For voltage clamped cells, cells were clamped either at − 70 mV to measure synaptic excitation (approximate reversal potential for inhibition), or at + 10 mV to measure synaptic inhibition (approximate reversal potential for excitation), uncorrected for the junction potential. Series resistance was monitored on every trial with a negative voltage step. Cells were only included if their series resistance stayed within 20% of their initial value, passively or by adjusting pipette pressure.

#### Visual stimulation

Visual stimuli were generated with Psychophysics toolbox^65^ using custom software in MATLAB (MathWorks) and pre­sented on a gamma corrected 23-inch Eizo FORIS FS2333 LCD display with a 60-Hz refresh rate. Stimuli consisted of drifting square wave gratings with contrast, size, or orientation varied, while all other parameters remained fixed, at 0.04 cycles per degree and 2–2.5 cycles per second. In experiments with varying contrast, size was fixed at 12 degrees, and the orientation fixed at the preferred orien­tation of the cell (measured via spike rate, V_m_ depolarization, or mean synaptic excitation). In 7/15 cells contrast was varied in six log increments from 1%–100%, and in 8/15 cells from 10–100%. In experiments with where size varied in six log increments from 2.4 to 59.4 degrees, contrast was set at 100% at the orientation set as above. In experiments with varying orientation, size was fixed at 12 degrees, contrast was set at 100% and orientation was varied in eight different directions spaced by 45 degrees from 0 to 315 degrees. The grating drifted immediately upon display, and lasted 0.6–1.5 s. Inter-trial-intervals (gray screen) lasted from 1.5–3 s.

### Analysis methods

#### Tests and measures

For making claims about whether one algorithm performed significantly better than another set we used the Wilcoxon signed-rank^66^ method implemented with MATLAB. This tests whether the median difference between two matched sets is significant. When comparing whether synaptic excitation or inhibition performed better for the same algorithm while limiting the comparison to just one stimulus variable (orientation, size, contrast) we used the Wilcoxon signed-rank test because each cell was recorded from in both modes. If we were making the same comparison across multiple stimulus variables, or comparing membrane potential to other recording modes we used the Wilcoxon rank-sum^66^ test because the comparison includes different sets of cells. We used exclusively one-tailed tests chosen based on the difference of medians. The significance level was kept at 0.05 for figure annotations. There were 10 data categories often including data from the same cell but with a different recording mode (voltage clamp at either the excitatory or inhibitory reversal potential). We reinforced comparisons across experiments (algorithm versions) by pooling data and assessing the discriminating statistic overall. Thus we were interested in the comparison wise error rate and did not need to adjust for multiple comparisons because we did not claim that one algorithm was better than another on the basis of an individual category^67^. We highlighted individual comparisons on categories across and within algorithm versions to make specific claims about those categories. The notion of significance being open to interpretation^67^, we elected to simply publish the P-value as well as the number of categories and let the reader make the final judgment. The effect size is the simple difference formula^68^, chosen because it is a normalized measure weighting the median difference between two matched sets against the size and frequency of cases that contradict the median difference and helps evaluate the meaningfulness of a judgment about significance.

We used two measures of performance, the fraction of trials for which the co-occurring stimulus was correctly predicted, the correct classification rate “CCR” and also the F_1_ score^69^. The correct classification rate is the fraction of data points that were assigned the correct label. In our case labels were the ordinal rank, or index, of a drifting grating property as the stimulus label. The F_1_ score is widely used throughout machine learning. It applies to binary classification and is useful in cases with skewed class sizes. This makes it a useful measure of discriminability for individual stimuli. The F_1_ score is defined with respect to a specific stimulus label. F_1_ = 2⋅T_P_/(2⋅T_P_ + F_P_ + F_N_) where T_p_ are the number of true positives regarding that stimulus label, F_P_ are the false positives, and F_N_ are the false negatives. This value ranges between zero and one. We used the average F_1_ score across all stimulus labels as the objective function error measure because it penalizes cases where the classifier learns to predict one or two stimuli correctly at the expense of predicting others. The CCR is not resilient to this error but is familiar to a broader audience.

#### Characterizing recorded responses with deflection and epoch

All experimental signals were recorded at 20 kHz and downsampled to one kilohertz by means of 20 ms averaging. When removing spikes from membrane potential recordings the downsampled signals were further processed with a median filter with a five millisecond window. Simulated data was produced at 10 kHz and downsampled to one kilohertz by 10 ms averaging.

We calculated deflection by obtaining a baseline and subtracting that from the average signal during an epoch of choice (see Fig. 2a). The baseline was found for individual trials by identifying a period of minimum variance that was 100 ms long and had a start time between 200 and about 100 ms before the onset of stimulus response. No baseline ever included a portion of a stimulus response in its estimation. The exact timing that the monitor displayed the stimulus was the “on” timing in Fig. 2a. It was not recorded, instead we made a conservative estimate to mark a time before any stimulus response was likely to reach V1 given retina to V1 latencies (see below). For the purpose of stimulus discrimination, we tested different lengths and positions for interval used to define deflection. We used a 166 ms window which contained the peak in average response during the epoch of choice (see below) and began either 66 ms before the peak, or halfway between the peak and the epoch start time if the peak was within 66 ms of the epoch start time. The deflection for each trial was the average value of the baseline subtracted from the average value within this window. This formulation gave the highest ability to classify stimulus by using the deflection. Therefore, this was the fairest definition of deflection to use for comparison to dynamical discrimination. In previous work with this same data, the term “response” was used^21^. The key difference being the length and location of the second window. The researchers integrated the difference from baseline without dividing by the length (i.e. it was not a mathematical average but proportional). This was tested and compared against our use of deflection. The absolute value of deflection was used in analysis.

We identified the timing of stimulus onset as follows. For each cell and type of recording (excitatory current, inhibitory current, and membrane potential) we averaged all recordings and applied a 50 ms running average to the mean recording. We then identified the largest extrema in the first half of the mean recording. The mean recording was then binned by 50 ms intervals and a first order derivative estimate computed. The largest derivative immediately prior to the extrema denoted the bin in which the response began on average. Because it takes 70 -150 ms for activity to propagate from the retina to V1 L2/3 neurons^70^ we subtracted 100 ms and rounded to the nearest half bin-width, 25 ms. This was finally defined as the “on” timing and occurs well before stimulus response. Therefore, we captured the full response even allowing for variability in time of onset. The length of the stimulus presentation also varied, from 500 to 1000 ms. So, the “off” timing also varied.

The response to stimuli is commonly categorized into three distinct epochs with much study and debate about their role in sensory processing^34,71–81^: the “on response” coinciding with the activation of a stimulus, the “steady-state” response which captures what follows the on response while the stimulus is still active, and the “off response” which is a widely observed perturbation or lingering effect after the stimulus has ceased. We did not know which epoch would allow the best discrimination. Some evidence pointed toward fast attractor dynamics in the on response^16,34^, but we treated stimulus response epoch as a hyperparameter and captured differences in the performance of our dynamical discrimination algorithm (see supplemental S2). Referencing both the on and off timings as t_0_ and t_f_ respectively we defined the “on response” as [t_0_, t_0_ + 250 ms], the “full response” as [t_0_, t_f_ + 250 ms], and the “off response” as [t_f _− 70 ms, t_f_ + 250 ms]. The discriminability of trajectories from these three epochs have some scientific merit in their own right and are reported in the supplemental S2. These choices allow us to examine performance under different circumstances and thereby optimize classification performance by using domain specific insights.

#### Deflection-based decoding

To create a decoder using deflection from baseline we used a random forest classifier (MATLAB’s default parameters). Each decision tree within a random forest classifier divides the range of possible deflections into smaller intervals. Each interval corresponds to a best-guess stimulus. Each decision tree uses a slightly different portion of data to select intervals. Therefore, each decision tree is affected by outliers differently and has a slightly different set of intervals. They then vote on a classification. This was done individually for each cell in each data group. For membrane potential data groups, spikes were always removed prior to calculating deflection as described above.

#### Single neuron modeling

We used models of a single-compartment Hodgkin-Huxley type neuron and a morphologically complex pyramidal neuron implemented with the NEURON environment. The morphologically complex pyramidal neuron was developed for other research^36^ and extensively explained. It is available for general use from model dB^82^. It was modified to allow experimental manipulations of the number of synaptic spines distributed across the various compartments and having one synapses for each spine and to allow each synapse to be driven with independent signals. Consult our model data generation scripts for model form and parameter details.

For both neuron types we rescaled and resampled the X dimension of the Lorenz system.

The Lorenz system^83^:

$$ \begin{aligned} \frac{dX}{{dt}} & = \sigma \left( {Y - X} \right) \\ \frac{dY}{{dt}} & = X\left( {\rho - Z} \right) - Y \\ \frac{dZ}{{dt}} & = XY - \beta Z \\ \end{aligned} $$

was integrated using MATLAB’s ode45 for 10 K time steps with a nominal step size of dt = 10^–4^, initial conditions were randomly chosen and uniformly distributed between [− 16, 16] for the X and Y dimensions, and [− 56, 56] for Z. After generating data, the X dimension was kept and the other dimensions were not. The Hodgkin Huxley equations act as a low-pass filter and our single-compartment model distorted input current oscillations above 333 Hz. Therefore if the integral of the squared absolute value of a fast-Fourier transform of the signal from 0 to 300 Hz accounted for less than 90% of the total integral then the trial was resampled $X^{{\prime}}(t)=X(t/\tau )$. Resampling was done by regenerating new Lorenz data with a false time step dt′ = dt/τ, where τ (in units of 10^–4^ s) was chosen such that 90% of the signal power was in Fourier modes below 300 Hz. The process was repeated until a set of initial conditions and τ were found that satisfy our acceptance criteria. Let *χ* be the Fourier transform of *X* in the frequency domain, then:

$$\tau =\frac{(0.9{\int }_{0}^{\infty }|\mathcal{X}(f){|}^{2 }df) }{({\int }_{0}^{300}{\left|\mathcal{X}(f)\right|}^{2}df)}$$

We treat the ρ parameter as a latent variable we were attempting to identify and vary it between the integers^20,40^. This lets us test whether we can predict fine changes in a dynamical parameter (ρ) even after transforming the dynamics into the membrane potential of two classes of model neuron. Our method of attractor reconstruction, delay embedding, is robust to arbitrary projections of a dynamical system onto one dimension. A weighted adjacency matrix typical of network model is an example of such a projection, except for the additional transformation from a continuous time series to a discrete point process which is often used. Nonetheless it has been shown that a spiking network can encode Lorenz attractor dynamics which can then be viewed by projecting high-dimensional population spiking onto a lower dimensional state-space^14^. Therefore, it was sufficient to project the Lorenz dynamics onto a single dimension and stochastically encode that dimension with discrete events if we want to study the representation of network attractor dynamics by single neuron inputs. We did this while taking the ρ parameter through values on either side of Hopf bifurcation at $\rho =\frac{\sigma (\sigma +\beta +3)}{(\sigma -\beta -1)}\hspace{0.17em}\approx \hspace{0.17em}24.7$ and into regimes where initial conditions often result in chaotic dynamics.

For the single-compartment neuron we rescaled *X’(t)* to be within a realistic range for current units [− 0.15 nA, 0.15 nA], $I(t)={\alpha }_{1}(X^{{\prime}}(t)-{\zeta }_{1})$. The scaling factors ζ_1_ and α_1_ were fine-tuned by a loop which adjusts them to produce a desired mean spike rate for a group of 210 trials (21 values of ρ each with 10 initial conditions unique to each value of ρ). This produced a range of spiking values and was repeated until there were at least three trials for each ρ value for each desired number of spikes [0, 20] per one second trial. This gave us multiple trials for each value of ρ at each level of spiking we were interested in testing. This method for generating the desired amount of spiking was inexact but preferable to search algorithms that precisely controlled the spiking in every trial. These algorithms took a long time to find solutions and often found undesirable solutions, such as scaling the inputted current to have a standard deviation near zero. Consult our model initialization scripts for model details.

For the complex morphological neuron we rescaled *X’(t)* to be consistent with an instantaneous event probability for an inhomogeneous Poisson process:$P(t)={\alpha }_{2}(X^{{\prime}}(t)-{\zeta }_{2})$. The factors ζ_2_ and α_2_ were further fine-tuned the minimum and maximum value to get a desired mean spike rate for a group of 210 trials, as with the single-compartment model. Initially we set the range and maximum value to be 3 × 10^–3^. We vary the number of synapses between 80 and 300. For each synapse, the probability of firing at any time was $P(t)$. Thus larger $P(t)$ values resulted in greater synchronicity among synaptic events. Synaptic transmission was modeled with NEURON’s Exp2Syn function which is a two-state synapse with a rise time of 0.2 ms and a fall time of one-millisecond. The peak synaptic conductance, g_max_ (units of μS) was a function of the number of synapses N_syn_ such that if K% of synapses were active the total peak conductance was independent of N_syn_: g_max_(N_syn_) = g_0_(80/N_syn_) where g_0_ = 5 × 10^–4^ μS.

We employ modeling to test the effect that realistic sources of error may have on the ability to infer the dynamics which underlie neural inputs. The two key variables that we controlled for were spiking and synapse numerosity in an extended multi-compartment model. To that end we defined 15 logarithmically increasing bins to contain model trials with similar levels of spiking. These bins started with [0,1), ended with the 14th bin [39,50) and the 15th bin [50, ∞). Since the single compartment neuron did not feature synapses these bins specified all control categories. Not all bins were populated. Each bin contained multiple trials for each value of ρ. If there were fewer than three trials for a value of ρ then those trials were ignored and not analyzed. If there were fewer than 45 total trials in a spiking rate bin after removal of underrepresented ρ values, that bin was ignored and none of the trials in that bin were analyzed. As a result, there were 12 spiking categories that were analyzed for single-compartment neuron data. The three unfilled bins were for trials with more than 20 spikes, which was our minimum standard when generating data. The morphologically complex multi-compartment model neuron has two variables to control for: spiking and synapse numerosity. For each spiking bin we defined several synapse bins to categorize trials in two dimensions (spiking and synapse numerosity). There were more model trials with low numbers of spiking due to the inexact way we generated the desired amount of spiking (by the average spiking rate of 210 trials). Because trials with a small amount of spiking were overrepresented it was advantageous to define more synapse bins for the spike bins containing fewer than five spikes per trial. Thus, there were 19 synapse bins for each of the first five spike bins going from 80 synapses to less than 125 in steps of 10, then 125–305 in steps of 15. There were 16 synapse bins for the remaining spike bins, going from 80 to 305 in steps of 15. Thus, there were 255 possible bins and 83 were accepted for analysis. Again, each bin had to contain more than 45 total trials and each ρ value had to have more than two trials to be included. In the main text we plot a sampling of these categories, attempting to show how spiking impacts performance while keeping synapse numerosity approximately constant (Fig. 6e). All the resampled Lorenz dynamics inputted to each cell were preserved without rescaling and analyzed together as one group.

#### Dimensionality expansion

The version of time-delay embedding we perform is described in^25,84^, a short summary follows. First, we concatenated the epochs of interest from all recordings from the same cell to make a time series with length T. Next, we chose a delay time below or at the approximate smallest relevant time-scale, *dt*, (one-millisecond in our case) and a number of times to repeat the delay N_d_ = 100 such that the N_d_th delay was longer than or at the largest relevant timescale (100 ms). We then created a data matrix with N_d_ + 1 rows, each of which were time shifted copies of the data with length T-dt⋅N_d_ (called a Hankel matrix). Then we performed singular value decomposition on this Hankel matrix (we tested other dimensionality reduction algorithms). The principal components were now the dimensionality expanded version of the data. Thus, we simply over-expanded with time-delays then used dimensionality reduction to go back down to moderate dimension. There was no clear cut off in the eigenspectra, so we tested keeping between three and seven components by running each choice through the analysis program. We did not Z-score the rows of the Hankel matrix but did shift the trajectories resulting from dimensionality reduction such that the mean point of the entire set of trajectories was at the origin. A delay embedding is guaranteed by the Whitney-Takens delay embedding theorem to be able to reconstruct a D dimensional state space from a one-dimensional recording by taking no more than 2D + 1 delays of the recording and plotting them against each other^85^. We call this process “dimensionality expansion” to provide the intuition that if an analyst can do something after dimensionality reduction of high-dimensional data they can at least attempt it on one-dimensional data too. The analyst would simply use SVD on any over-embedding such as a spectrogram or such as a Hankel matrix like we have demonstrated.

#### Maximum likelihood estimation

It is possible that dimensionality expansion alone can improve discriminability between stimulus responses without appeal to governing dynamics. This would work because different population responses were projected onto somatic responses in a way that overlaps. Hence deflection in the original time series confuses these factors but dimensionality expansion may re-separate them. We tested measures of deflection in each dimension of the expanded trajectories, but this did not significantly improve classification. An alternative is to examine whether different trajectories prefer to spend time in different regions of phase space. We used the performance of a Maximum-Likelihood-Estimation-based classifier to quantify this separation (see supplemental S4).

Since trajectories in three dimensions tended to form oscillations around a long axis, cylindrical coordinates (axial *z*, radial *r*, and angular *θ*) were a natural way to describe them. We partitioned axial and radial coordinates into discrete bins and ignored the angular coordinate then counted the number of time points that coincided with each bin. Thus, each trajectory was described by a histogram of its axial cross-section. We collected the cross-section histograms associated with each stimulus, using 75% of the examples for each stimulus. Then we employed a two dimensional kernel smoothing density to get a two dimensional probability density map for each trajectory *M*_*t*_*(z,r;i)* where *i* is the trajectory index (see supplemental Fig. S10a) and for each stimulus *M*_*s*_*(z,r;j)* where *j* is the stimulus index (see supplemental Fig. S10b). Each point (*z*, *r*) identifies an axial-radial bin and was assigned the probability that a trajectory time point selecting at random will be in its bin. This gives a set of probability maps that show the probability for a trajectory to occupy a region of phase space (a cross-section bin) dependent on each stimulus. Thus to test whether one of the 25% of trajectories we held out coincided with the presentation of a particular stimulus we used the probability maps to calculate the joint probability of observing all the time points given that stimulus (treating each point as independent), $P(i;j)={\prod }_{z}{\prod }_{r}{M}_{s}(z,r;j)\cdot {M}_{t}(z,r;i)$, for practical application we used the log likelihood $L(i;j)={\sum }_{z}{\sum }_{r}-(log({M}_{s}(z,r;j))+log({M}_{t}(z,r;i)))$ The stimulus whose probability map yields the highest joint probability was the stimulus with the highest likelihood of co-occurring with the trajectory and thus was the classification, ${C}_{j}={argmax}_{i}L (i;j)$. By repeating this process 510 times with a different hold-out set each time we can gather sufficient statistics to gauge whether this prediction method was effective.

A detail essential for reproducibility is that the trajectories co-occurring with each stimulus show some displacement between their central axes and central points. Thus, to get joint probabilities, one must subtract the central point of each stimulus-associated trajectory set before computing *M*_*s*_*(z,r;j)*.

#### Tuning curve reliability

A tuning curve is defined as mean deflection in response to stimulus. Therefore, we recorded the average evoked deflection for each stimulus, *D*_*i*_, where *i* is the stimulus index out of *N* stimuli. For a single cell and intracellular recording method, the stimulus that evokes the largest deflections on average is the most preferred stimulus, and the one that evokes the smallest deflection is the least preferred stimulus. The least preferred stimulus is not necessarily the stimulus that is least similar to the preferred stimulus (i.e. not the anti-preferred stimuli). Since stimuli were continuously and monotonically varied along one parameter (orientation, contrast, size) we used the ordinal number difference between two stimuli as a similarity metric. Stimuli were indexed by an ordered ranking. Thus, stimuli numbers one and six would be the smallest and largest drifting grating if size were varied and they would also be maximally dissimilar, while five and six would be the largest and second largest and maximally similar. We defined reliability as the mean deflection in response to the same stimulus divided by the standard deviation of deflections in response to that same stimulus, *R*_*i*_ where *i* is the stimulus index out of *N* stimuli. In order to measure the correlation between reliability and similarity to either the most or least preferred stimulus we defined a least/most similarity function, *S*_*i*_ as follows. The ordinal values of the most and least preferred stimuli were recorded. For all stimuli, the absolute value of the ordinal difference between themselves and the least preferred stimulus was noted, then the same was noted for the most preferred stimulus. The smallest of these two absolute ordinal differences was kept. Thus, for each stimulus we have recorded the absolute ordinal difference between itself and either the most or least preferred stimulus (depending on which difference was smaller). We then divided by the number of distinct stimuli (either six for size or contrast, or eight for orientation). The least/most similarity function was then: ${S}_{i}={\operatorname{min}}(\{|i-{\underset{0<j\le N}{\operatorname{argmin}}}({D}_{j})|,|i-{\underset{0<j\le N}{\operatorname{argmax}}}({D}_{j})|\})/N$. We also have the reliability measure as defined above. We measured the Pearson Correlation between reliability, *R*_*i*_, and this least/most similarity score *S*_*i*_. Because this measure was applied individually to each cell it quantifies whether the U-shaped trend in Fig. 5b was a property of cells individually. If *D*_*i*_ tended to be proportional to *R*_*i*_ for some cells and inversely proportional for other cells, then Fig. 5b may still appear U-shaped but there would be no correlation between *R*_*i*_ and *S*_*i*_.

We also explored possible causes for changes in reliability. These include the coefficient of variation of a response and the mean of normalized residuals. The mean of normalized residual is a measure of noise. To calculate it, we first calculated the average response of a stimulus to all repetitions of a stimulus. We subtracted this mean response from a single trial, the result was a residual time series. To normalize the residual time series, we divided by the mean response time series from the first step. Therefore, each point in the new time series was the signed fractional error between the single trial and the mean response. The average value of this time series was defined as the mean normalized residual. We also examined a variant where we computed the absolute value of the normalized residual time series before computing the average. For any given recording we calculated its normalized residual by comparing to the other responses to the same stimuli. We compared noise with fluctuation size. We defined fluctuation size as the coefficient of variation, which is the standard deviation of a timeseries divided by the mean value of the same time series. Thus, both measures control for average deflection. These measures let us test whether decreased noise or decreased fluctuation size was responsible for increased reliability.

#### Genetic algorithm modification of SINDy

We used the SINDy algorithm because it is well supported and amenable to modification^25^. In short, we pre-computed the derivatives, dV, of a singular value decomposition base time-delay embedding^25,84^ (dimensionality expansion),V, of a single time-series. We then pre-computed many polynomial combinations of the original data (including a constant term) Θ. A dynamical system was therefore captured by a matrix Ξ projecting the polynomial combinations onto the derivatives dV = Ξ^T^Θ. The critical insight is to set most of the elements of Ξ to zero so that the dynamical system is readable, tractable, and generalizes to the rest of state-space. Originally^25^, elements of Ξ were chosen to be non-zero by identifying thresholds through hand tweaking such that only Ξ elements exceeding the thresholds were included in the fit. Instead we used a genetic algorithm to automatically decide which elements to set to zero without a threshold. For simplicity we call this “genetic SINDy”. Note that if one specifies the locations but not the values of nonzero elements with a binary-valued bitmask Ξ matrix, ^B^Ξ, then a ^B^Ξ is a template which can generate diverse kinds of Ξ matrices because fitting the coefficients specified by ^B^Ξ to two different trajectories would produce two different Ξ. The original work showed that using time-delay reconstructions of undersampled systems yields Ξ matrices that are characteristically non-sparse in the last dimension^25^. The original work also noted that it requires long periods of time in diverse situations to capture the best invariant models of the system. This is a characteristic we exploited to get local approximations instead of the invariant models the original work sought to obtain.

In order to avoid numerical error, one must normalize Θ. A key difference is that the original paper^25^ divided by the L^1^ norm but we had better results by Z-scoring^86^ each variable (row of Θ) and always including a constant term in Ξ. This forced information about the average variable value into the constant term, making it available for the classifier in later stages. We tested the inclusion of second order derivatives, d^2^V/dt^2^, and inclusion of more dimensions, as well as numerous other variations but settled on three dimensions and first order derivatives, dV/dt as giving the highest utility with the least complexity and compute time (see supplemental S2). Another departure from the original implementation of SINDy^25^ was the addition of a single three time-step smoothing window after estimating derivatives using the fourth order method. We did not rigorously compare the inclusion of non-polynomial forms as performance was good enough that we could test our hypothesis without the additional complexity.

A genetic algorithm must be initialized with a diverse population of individual “guesses” at a solution to the problem. In our case an individual solution was a bitmask matrix, ^B^Ξ, the same size as Ξ, but consisting only of zeros and ones. Ones marked the location of Ξ elements to keep as non-zero when creating an ODE model in later steps. To get an initial population we used an unsupervised threshold method to decide which elements of ^B^Ξ were one and which were zero (included with our software). We treated each trajectory individually and used bisection search to find the largest threshold (for each column of ^B^Ξ) that resulted in at least one non-zero element. This gave a maximally sparse representation and largely reproduced the findings reported elsewhere^25^ when used on a fully sampled Lorenz system. The result was a set of unique ^B^Ξ that was no larger than the number of trajectories.

We used a “mating” (crossover) process to create 300 unique individuals. For each ^B^Ξ in this set we obtained Ξ matrices for each trajectory. Twenty five percent of the trajectories were held out for testing, the remainder were used to find the coefficients of the system of equations. After testing fitness, the best 45 ^B^Ξ were “mated” and “mutated” to generate 300 new forms of the equation. A “sexual genetic algorithm” requires a method for combining possible solutions, a “mating” process. This involves three steps, selecting individuals to combine, deciding which attributes to keep in the “offspring”, and a way to mutate the offspring. We kept the best 45 unique ^B^Ξ and ranked their performance (worst is 1 best is 45). We then used 255 tournaments to select parents, mate them, and produce 255 new ^B^Ξ matrices. One parent was selected by cyclically stepping by one through the best 45, the second parent was selected at random with a probability in proportion to its rank. A parent was not allowed to mate with itself. The nonzero elements of children were selected by keeping elements which were nonzero in both parents and with probability one-half if it was nonzero in only one parent. Finally, mutation was implemented by flipping one or more randomly selected elements to its opposite value. Each element of the child matrix was subject to mutation with a specific probability called the “mutation rate”. The mutation rate was 0.15 to produce the initial population. It was set to 0.05 for the first generation and was periodically halved until it was set to zero for the last ten percent of generations. The number of halvings depends on the initial mutation rate, ${r}_{mut}$,and the number of elements in the Ξ matrix, ${N}_{\Xi }$, according to ${N}_{mut}=\lceil {log}_{2}({r}_{mut}\cdot {N}_{\Xi })+1\rceil$ and was not allowed to be smaller than 2. We tested other methods for mating on small fractions of original and simulated data, including: transferring columns or rows to the children intact, selecting half of the elements from each parent (either at random or in a structured way), or simply keeping all elements which occur in any parent. The choice of mutation rate and halving periods, as well as mate selection, mutation methods, the population size, and fraction to keep were selected by hand tweaking on fractions of data and simulated data.

The number of generations to run the algorithm increased by 100 for every three columns of the Ξ matrix (which may include second derivatives as well as higher dimensions). If the errors of the 45 ^B^Ξ matrices were identical or within one one-thousandth of the range of errors in the initial population then the algorithm was terminated early. This never happened when the objective function for the algorithm was classification ability and happened only rarely when the objective function was goodness of fit. The number of generations was tested by hand to be long enough to ensure convergence but not long enough to produce over fitting.

In our case different ^B^Ξ represented different possible solutions and we had three objectives to consider. We desired a Ξ matrix which can be fed into a classifier and perform well, we desired that this Ξ matrix be sparse (to avoid overfitting and improve interpretability) and lastly we desired that the Ξ matrix describes a good model of the dynamics. The objective function for classification ability was noisy because the hold-out set was small for the generation updates, (see below), therefore we retested the best 45 at each generation.

#### Classification objective function for genetic algorithm

In order to for the genetic algorithm to find the best binarized template to fit Ξ matrices, a ^B^Ξ matrix (see Genetic algorithm modification of SINDy above), it must have a standard to measure against. We compare performance when using two standards: 1) finding the ODE model which leads to the best classification performance and 2) finding the ODE model that simply fits the data the best without regard to classification. No matter what standard is used to create the ^B^Ξ matrices, the ^B^Ξ matrices are used for stimulus decoding in a later step (see “Ensemble classification and out-of-sample generalization” section). For genetic algorithms, the standard used to measure individuals is implemented through an objective function. An objective function accepts a ^B^Ξ matrix and outputs a scalar value which is lower for ^B^Ξ that are better at satisfying some objective. For predicting the stimulus based on the coefficients of a fitted ODE the genetic algorithm objective function started by fitting Ξ matrices to each trajectory using only the coefficients specified in the ^B^Ξ matrix it accepted as input. Next, for each stimulus, 25% of fitted Ξ matrices were held out for cross validation. MATLAB’s default implementation of random forest^69,87^ was then trained on the remainder (the 75%) and tested on the cross validation set (the 25%). This was repeated 10 times, selecting a different twenty five percent each time (this is sometimes called hold-k cross validation with bootstrapping^88^). The classification performance was the average F_1_-score for all stimulus labels^69^. We subtracted this value from one such that good performance was a low number that still ranges between zero and one and constituted 80% of the objective function value. The other 20% of the fitness value was a regularization term: the fraction of possible terms which were nonzero (i.e. sparseness). It should be noted that the random forest used is disposable, being recreated for each hold out set and uses MATLAB’s default objective function for growing decision trees.

#### Goodness of fit objective function for genetic algorithm

For finding a set of coefficients (specified with ^B^Ξ) that allowed the highest quality ODE model, the objective function started by fitting Ξ matrices to each trajectory using only the coefficients specified in the ^B^Ξ matrix it accepted as input. Next it used all the original points on the trajectory as initial conditions to integrate the fitted ODE four timesteps. Goodness of fit was 1-R^2^ (the coefficient of determination) between the derivatives predicted by integrating the ODE and the derivatives of the data shifted by five timesteps. A sparseness regularizer was used such that goodness of fit was 80% of the objective function value and sparseness was 20%. As a final step we evaluate the usefulness of these models for performing classification if fed into MATLAB’s default random forest classifier. Performance was evaluated using the average F_1_-score for all stimulus labels^69^.

#### Ensemble classification and out-of-sample generalization

The genetic algorithm tested one ^B^Ξ matrix at a time, however each matrix was pulling out a different set of coefficients and therefore might have been highlighting different dynamical attributes. It is often found that an ensemble of independently trained classifiers can cooperatively vote on a classification and that doing so often cancels out bias that cropped up during the training of any individual^89^. We found that ensemble methods decreased overfitting tendencies for dynamical discrimination. Therefore, the 45 ^B^Ξ unique matrices which were best in the final generation of genetic SINDy voted on making a final classification to complete our process called “dynamical discrimination”. When we were training random forest^87^ regression trees instead of classifiers we used the median of the ensemble. For classifiers we used the mode with ties broken by choice with the highest ranked ^B^Ξ according to final generation fitness.

Because the cross-validation process was repeated once every generation there was information leakage and over-fitting effects were possible for the algorithm as described. To measure overfitting, we re-ran the entire genetic algorithm and ensemble process on data where the stimulus labels were scrambled. Performance on random surrogates was stable, despite high variability in the performance on the original data (Fig. S2b). Random surrogate performance was indistinguishable from chance for deflection and classification based on Ξ that were fitted without regard to classification performance (Fig. 3a, S2b).

In order to report values without confusing overfitting for reproducible (out-of-sample) performance we encapsulated the entire genetic algorithm and ensemble process in another layer of holdout testing. We held out one example of presentations for each of the 6–8 stimuli and ran the entire algorithm, then predicted the stimuli for the examples which were never used for training. This was repeated twenty times and the final values reported in the main text (Table 1) and in Fig. 3b, are the results of this 20-fold hold-one-out test. Using the data from the cell with the most trials, we verified that the final holdout did not perform better than chance when trained on random surrogates. Therefore, the final holdout performance is a valid out-of-sample generalization. Performance of the ensemble was evaluated using both classification success rate (Table 1, Fig. 3b) and the F_1_-score for individual stimulus labels^69^ (Fig. 3c, S13–S18).

#### Hyperparameter optimization

Augmenting Sparse Identification of Nonlinear Dynamics (SINDy) with a genetic algorithm to provide a representation of time-series suitable for a random forest classifier requires many choices which can affect outcomes. Research that makes scientific comparisons but does include an account of hyperparameter optimization cannot be adequately reproduced or checked for bias. With respect to SINDy performed on delay-embedded (dimensionally expanded) data one must decide what size of delays, how many delays to include prior to dimensionality reduction, and what dimensionality reduction algorithm to include as well as a slew of ODE related choices such as what polynomial order to include, whether to include other non-polynomial forms such as sinusoids or sigmoids, whether to include a quenched-noise driving term (treating extra dimensions as a time-varying input)^25^, how many dimensions to include, how to get stable numerical estimates of derivatives, whether to use higher-order derivatives, how to normalize the data, and preprocessing steps such as detrending, and filtering and what period of the timeseries to fit. A genetic algorithm requires even more choices such as methods for mate selection, crossover, mutation, as well as what terms to include in the objective function, how to weight those terms, and stopping conditions. Even the selection of a classifier algorithm to perform the last step presents a set of choices that can alter a scientific comparison.

Few of these choices can be made a-priori, and there were too many to test completely. Choices such as these are known as “hyperparameters”. They must be reported on to demonstrate that all effort was made to maximize the performance of machine learning algorithms before making claims about comparisons. Most choices were made by hand testing on small fractions of data or on simulated data. Some choices made little difference, some made the algorithm worse, others could not be justified due to the exorbitant computational time required. These choices are important to be aware of for replicating results but are not extensively reported on here, except to state them: we chose 100, one millisecond delays and chose Singular Value Decomposition (SVD) to reduce from 100 to between three and seven dimensions (and extensively tested this narrowed range). We included only polynomial terms up to the third order (because it is the same order as a FitzHugh-Nagumo ODE), and classification did not benefit from including an additional dimension as a quenched-noise driving term. Derivatives were estimated by using a custom algorithm based on standard fourth order derivative methods^25^ but added smoothing (window size three timesteps) as a last step. Derivative estimation methods were chosen to ensure the derivatives matched the trajectory when accumulated. Data were not detrended but were downsampled to one Kilohertz. After dimensionality expansion the entire set of embedded trajectories was centered at the origin. The choice of epoch, number of dimensions and derivative order were tested with a more exhaustive optimization approach. For the genetic algorithm, mate selection, crossover method, and mutation method, as well as the size of the population and the fraction to keep at each generation were all tested on small fractions of the data (and choices stated above). The choice of regularization factors and terms to include in the objective function were narrowed by hand on a small fraction of data and then a few remaining options were exhaustively tested. There were 51 trees in the random forest algorithm for final results and five trees for evaluating the classifier objective function in intermediate generations of the genetic algorithm. Alternative methods of classification, such as fitting ODEs to the trajectories co-occurring with a specific stimulus and then assessing which ODE best-fit a test trajectory, yielded such poor performance or additional complexity that they did not justify inclusion in this paper. The inclusion of additional constraints such as a goodness-of-fit constraint in a classifier objective function did not perform well enough to justify the testing needed to find the optimum choice. It was found that an early termination stopping condition based on convergence to a narrow range of error values achieved the same results as a stopping condition based on a lack of diversity among possible solutions. The choice of 100 generations for every three columns of ^B^Ξ was made by hand testing.

Some choices were selected for exhaustive testing because they either had scientific value: period of stimulus presentation to train on, inclusion of second order derivatives, and how many dimensions to include. Others were chosen for exhaustive testing because the effect was complexly related to other factors being tested: regularization factor, choice of dimensionality reduction (SVD versus independent component analysis), and whether to use an ensemble method. These were tested by running genetic SINDy on either all of the data or just on ^***I,O***^***I***, or ^***I,S***^***I ***and comparing the cross-validation of the final generation with the same for random surrogate data. The parameter set with the best classification ability with the least variability and least overfitting was selected. This was three dimensions with a regularization weighting factor of 0.2 and only first order derivatives, using an ensemble classification method with SVD for dimensionality reduction and the period coinciding with the onset of stimulus response was the most informative. These testing results are reported in supplemental S2.

#### Integration of Ξ matrices

Because we normalized Θ components by Z-scoring^86^ them we had to carry out a change of coordinates at each step when integrating the ODE models to assess how they captured trajectory details. Let ℰ be the function that creates polynomial combinations of a trajectory, V, such that Θ = ℰ(V). When integrating the ODE to create a simulated trajectory, V′(t) = dV′(t−1) + V′(t−1), the derivative term at each timestep becomes dV′(t) = Ξ^T^[ℰ(V′(t−1))−μ_Θ_]/σ_Θ_, where μ_Θ_ and σ_Θ_ are the mean and standard deviations of each column of the Θ used to estimate Ξ from the experimental data.

We used points from the initial trajectory and found that our models tend to be difficult to integrate. Even with a stiff ODE solver initial conditions frequently “blew up” wherein the derivatives became exceptionally large, or the derivatives rapidly extinguished. Therefore, we tested multiple initial conditions and plotted the ones that produce trajectories which remain in the neighborhood of the original trajectory for as long as the original trajectory was, and that explore a volume similar to the original trajectory. This was done by rejecting initial conditions that produce trajectories whose standard deviations (along each dimension) were all less than five times the same standard deviations of the original trajectory and exceeded one twentieth of the original trajectory.

#### Linear stability analysis

For non-linear dynamical systems such as those approximated with SINDy the behavior in the vicinity of a fixed point is often analyzed through the eigenvalues of a Jacobian matrix evaluated at the fixed point. A Jacobian matrix is the matrix of all partial derivatives with respect to the main variables. We can evaluate this using the chain rule and our normalization factors. Normalization factors included a translation as well as a rescaling. Using MATLAB’s symbolic toolbox, we solved the ODEs for coordinates where the derivatives became zero. The translation did not affect the Jacobian but did need to be accounted for when solving for fixed point coordinates the same way it was accounted for when integrating (see above). We report on all real-valued fixed-points. We evaluated the Jacobian for the best one of the 45 Ξ matrices according to their fitness values on the final generation of the genetic algorithm. We evaluated all fixed points for each trajectory individually. If the eigenvalues of a fixed point have an imaginary component the dynamics are locally oscillatory, if the real component is positive they diverge away from the fixed point, if the real component is negative they converge towards it, if the real component is zero the dynamics form a cycle. If the number of fixed points changes when a system parameter (such as stimulus label) is changed then that parameter is said to take the system through a bifurcation, likewise if the sign of the real component of the maximum eigenvalue of the Jacobian changes. We report on the number of fixed points and the eigenvalues as a function of what stimulus was presented in supplemental S3.

## Supplementary information

Supplementary file1

## Data Availability

The data, data analysis software, and modeling software used in this study are freely available on the Open Science Framework at https://osf.io/9xke5/. The project title is “Precision multidimensional neural population code recovered from single intracellular recordings” and readers should begin with the file “Discriminate_visual_stimulus.m”. It should run on all MATLAB installations that have all the toolboxes installed. All functions and scripts necessary to reproduce results are there but the code is organized for a didactic illustration of the methods by running through a small amount of data with simple options selected, not a complete run through of the pipeline (which would take up to several months on some home computers). The file “Generate_model_data.m” contains our code used to generate model data and integrates with python. It is included for the purposes of completeness and transparency. Readers may refer to it to clarify methods as they work to replicate our findings. Unfortunately, it was set up for a specific software environment that we could not include along with it, it will take some work to make run on computers other than the computer it was developed on. A virtual machine clone of that computer is available upon request.
