# Supplementary material for: Precision multidimensional neural population code recovered from single intracellular recordings
Source: Sci Rep. 2020 Sep 29;10:15997. doi: 10.1038/s41598-020-72936-1 (PMC7524839; doi:10.1038/s41598-020-72936-1)
Supplement: Supplementary file 1 — Supplementary file1 [file 41598_2020_72936_MOESM1_ESM.docx]

Precision multidimensional neural population code recovered from single intracellular recordings

# Supplementary information

Author list:
Johnson, J.K.­­^1^; Geng, S^1^; Hoffman, M.W.^1^; Adesnik, H^2^; Wessel, R^1^;

Correspondence to:
 James K Johnson at jkjohnson@wustl.edu

Author affiliations:
^1^ Washington University in St. Louis
^2^ University of California, Berkeley

## Supplementary information table of contents

**Supplementary information 1**

**Supplementary information table of contents 2**

**S.1 Dynamical Discrimination as timeseries classifier: performance and additional insights 3**

**S.2 Hyperparameter optimization reveals epoch dependence of dynamical discrimination 12**

**S.3 Analysis of dynamical stability underscores an attractor dynamics interpretation 19**

**S.4 Maximum Likelihood Estimation of stimulus reveals dimensionality expansion is not sufficient 24**

**S.5 Closer look at dimensionality expansion of transmembrane current recordings and dependence on changes to orientation at the excitatory reverse potential 26**

**S.6 SINDy captures dynamics well if dimensionality is retained 28**

**S.7 Survey of tuning curves and dynamical discriminability 30**

**References to supplemental 37**

# S.1 Dynamical Discrimination as timeseries classifier: performance and additional insights

We offer a new method for analyzing electrophysiological data that brings neural population analysis to single whole-cell recordings. However, the same methods can be applied to any single-unit recording and the method can be easily adapted to multi-unit recordings. Applying discrete labels to time series of any dimensionality is a form of trajectory classification^1^. Examples include predicting the final destination of a vehicle based on a small sample of its path^2^, or identifying a fundamental particle from its path in a bubble chamber^3^. There are as many methods as applications. For neural data the goal is often to predict what stimulus^4–6^ or behavior^7–9^ co-occurred with the neural recording, but it could also be used to examine the impact of other experimental manipulations or observations. In brief all methods of trajectory classification seek to reduce trajectories to small sets of numbers which either correspond to a physical property (e.g. mass) or which is still abstract but small enough to train a standard classifier on. Since trajectories contain very large amounts of data (one data point for each dimension for each point in time) one would require very many examples if they used the whole trajectories as training data for a classifier such as a support vector machine, random forest, or neural network. These steps can be avoided by using recurrent neural networks such as LSTM, where it is known as “sequence classification”^10^. However, this usually requires large amounts of data to train on and is not model-dependent, therefore not easily interpreted or manipulated to gain additional insights. Attempts to use them for our whole cell recordings were not satisfactory. The trajectory classification approach we developed is an exciting new process because an experimentalist can test a hypothesized relationship by testing whether expected information is “encoded” in the dynamical rules governing the trajectory.

The method we use is an adaptation of the Sparse Identification of Nonlinear Dynamics (SINDy) algorithm^10,11^. This is rooted in more than convenience but in the attractor network theory of brain function. This framework seeks to model the brain as a network of dynamical nodes (a very high dimensional nonlinear dynamical system). With this perspective any neural response to stimuli with any degree of stereotypy is considered an attractor. The state space actually explored by the attractor is usually found to be a small fraction of the possible state-space, if possible state space is defined as an N-dimensional space where each dimension corresponds to the firing rate of each of the N-neurons. The much smaller subspace which is actually explored is often referred to as a neuronal manifold^8,10,11^. Currently the most common way to try to visualize and quantify this low-dimensional manifold is through dimensionality reduction on high-dimensional recordings. The use of nonlinear methods of dimensionality reduction have been able to reduce the dimensionality of neural data much further than linear methods such as PCA or SVD, and arguments have been made that nonlinear dimensionality reduction is a more faithful representation of the functionally relevant mechanisms^8^. An alternative is to expand one-dimensional recordings to a moderate dimensional space consistent with neuronal manifolds. The theory of non-linear dynamics offers some guarantees that this can be done, through the Whitney and Takens delay embedding theorems^12^. Delay embedding theorem guarantees that we can capture the high dimensional dynamics by observing any single dimension. Nonetheless it is unclear what information is lost as the neuron samples upstream populations, thus the relationship between dimensionality expansion on whole-cell recordings and the dynamics obtained through dimensionality reduction is unclear. Fortunately, our core inference mechanism is estimation of ordinary differential equations (ODEs) and ODEs can be fit to either dimensionally reduced or dimensionally expanded data. Therefore, if an analyst is using dimensionality reduction on multi-unit data and already has trajectories in a putative state space then the methods that follow apply without modification.

In the study of nonlinear dynamics, it is usually the case that the dynamics near an attractor are simpler than the dynamics describing the rest of state space. For example, in a system with many dimensions choices of model parameters and/or initial conditions near a limit cycle may be well approximated with simple harmonic motion whereas parameters at a bifurcation, or initial conditions far from a limit cycle may exhibit complex orbits^13^. This last simplification is what we depend on most for our method to work. We estimate the derivative of each dimension of our trajectories and we test numerous forms of 3rd order polynomial differential equations until we find a system of equations for each cell that has few terms but gives us the best ability to predict what stimulus co-occurred with each recording of a trial. We also explore models that are focused purely on goodness of fit and not classification. The fitting procedure is a least squares regression between selected polynomial combinations of our trajectory dimensions and the estimated derivatives. However, the selection of terms is carried out with a genetic algorithm (see methods). The result is that by taking the attractor computation perspective and fitting crude ODEs to short-duration dynamics we are able to produce a representation (the coefficients of ODEs) of single whole cell recordings compact enough with 12±1.5 real numbers (median ± half interquartile range) that we can train a classifier with 68 ± 18.75 total trials per cell and only 11± 2.5 samples of each type of stimulus for each cell with either 6 or 8 unique stimuli per cell. This representation is small enough that we can now perform classification despite having few trials.

Because we are performing trajectory classification to identify stimulus by finding systems of differential equations, we call our process “dynamical discrimination”. However, in order to perform the final classification step, we need a classifier. Sometimes, (e.g. particle detectors) the compact representation are basic physical properties like mass and charge^3,13^, and we need only to look it up in a table. In our case we have an abstract compact representation and have to create that look up table ourselves by training a random forest classification algorithm^3,13,14^. A random forest is an ensemble of decision trees. Each tree is a straight-forward conditional look-up table. The trees in the forest “vote” on the correct label to apply. This is among the simplest types of classifiers to use and can handle nonlinear problems. The trees are trained by taking a subset of the data that has already been labeled and finding patterns between the labels and the compact representations. The random forest is tested by seeing if those patterns allow a flow chart of true/false tests that ends with the assignment of correct labels for data that was not used for training.

Traditionally, neuroscientists have sought scalar summaries of intracellular recordings with the purpose of predicting a single neuron’s firing rate under the assumption that this is the most useful goal of single neuron observation^15^. The membrane potential of our neurons fluctuated between -72 mV and -62 mV in the absence of visual stimulation. Within 100 ms of activating a stimulus (an image presented on a screen) the cell membrane depolarizes by an amount which depends on many factors. The primary factor is whether the stimulus is a “preferred stimulus” for a neuron^16^. A preferred stimulus is one which is more likely than others to induce action potentials from that neuron. So, if a neuron happens not to fire an action potential upon presentation of a preferred stimulus the amount of depolarization of membrane potential should still be greater than for less preferred stimuli. It is well established that the amount of depolarization correlates strongly with firing rate and depolarization can be used as a proxy for firing rate when estimating feature selectivity^16,17^. This interpretation is frequently carried over into voltage-clamp recordings of inhibitory or excitatory transmembrane current^18^. In these cases, the term “deflection” rather than depolarization is more general so for simplicity we use deflection when discussing all signals. Deflection captures the difference in mean signal value during and not-during stimulus presentation. By using deflection we can compare with the previous accomplishments and understanding of whole-cell recording analysis which used the same or similar measures^19^.

The following table breaks results down the results of our dynamical discrimination algorithm by each kind of recording and stimulus. Given the cell -to-cell variability that exists it is necessary to judge whether the median value is indicative of the category behavior and one can expect to get the same results with other data sets. To that end we subtract the chance level of performance and use the Wilcoxon signed rank test^20^ to judge whether the median is better than chance and report on the effect size r_sdf_ (simple difference formula^21^ and its p-value. This table verifies the annotations of significance reported in figures 3, 4, and S10.

| \|  \|  \|  \| **One-tailed Wilcoxon signed rank test that CCR is greater than that of…** \| \| \| \| \| \| \| \| \| \| \| \| --- \| --- \| --- \| --- \| --- \| --- \| --- \| --- \| --- \| --- \| --- \| --- \| --- \| --- \| \|  \|  \|  \| **dynamical discrimination** \| \|  \| **best-fit Ξ based discrimination** \| \|  \| **deflection based discrimination** \| \|  \| **chance** \| \| \| **Algorithm and data groups** \| **median CCR** \| **N** \| **r_sdf_** \| **p-value** \|  \| **r_sdf_** \| **p-value** \|  \| **r_sdf_** \| **p-value** \|  \| **r_sdf_** \| **p-value** \| \| **All data groups pooled** \|  \|  \|  \|  \|  \|  \|  \|  \|  \|  \|  \|  \|  \| \| dynamical discrimination \| 0.2875 \| 110 \| - \| - \|  \| 0.1542 \| 0.01893 \|  \| 0.2022 \| 3.60E-08 \|  \| 0.2385 \| 5.49E-27 \| \| best-fit Ξ based discrimination \| 0.2797 \| 110 \| 0.09696 \| 0.9812 \|  \| - \| - \|  \| 0.1861 \| 1.54E-05 \|  \| 0.2427 \| 8.92E-24 \| \| deflection based discrimination \| 0.2281 \| 95 \| 0.04908 \| 1 \|  \| 0.0652 \| 1 \|  \| - \| - \|  \| 0.2218 \| 5.85E-14 \| \| MLE \| 0.2481 \| 110 \| 0.05633 \| 1 \|  \| 0.07046 \| 1 \|  \| 0.1351 \| 0.263 \|  \| 0.2246 \| 2.04E-15 \| \| dynamically stable state discrimination \| 0.2833 \| 95 \| 0.07487 \| 0.994 \|  \| 0.1254 \| 0.5063 \|  \| 0.1912 \| 2.73E-06 \|  \| 0.2377 \| 8.28E-24 \| \| **^I,O^I** \|  \|  \|  \|  \|  \|  \|  \|  \|  \|  \|  \|  \|  \| \| dynamical discrimination \| 0.1437 \| 7 \| - \| - \|  \| 0.219 \| 0.07813 \|  \| 0.1905 \| 0.1875 \|  \| 0.2429 \| 0.03125 \| \| best-fit Ξ based discrimination \| 0.1256 \| 7 \| 0.04762 \| 0.9453 \|  \| - \| - \|  \| 0.1429 \| 0.4688 \|  \| 0.1619 \| 0.3438 \| \| deflection based discrimination \| 0.1353 \| 7 \| 0.07619 \| 0.8516 \|  \| 0.1238 \| 0.5938 \|  \| - \| - \|  \| 0.1619 \| 0.1094 \| \| MLE \| 0.08612 \| 7 \| 9.52E-03 \| 0.9922 \|  \| 0.04762 \| 0.9453 \|  \| 0.07619 \| 0.8516 \|  \| 0.1333 \| 0.5313 \| \| dynamically stable state discrimination \| 0.1375 \| 7 \| 0.05714 \| 0.9297 \|  \| 0.1714 \| 0.2891 \|  \| 0.1333 \| 0.5313 \|  \| 0.2143 \| 0.08594 \| \| **^E,O^I** \|  \|  \|  \|  \|  \|  \|  \|  \|  \|  \|  \|  \|  \| \| dynamical discrimination \| 0.2562 \| 7 \| - \| - \|  \| 0.2476 \| 0.02344 \|  \| 0.2571 \| 0.01563 \|  \| 0.2 \| 0.01563 \| \| best-fit Ξ based discrimination \| 0.1908 \| 7 \| 0.01905 \| 0.9844 \|  \| - \| - \|  \| 0.2381 \| 0.03906 \|  \| 0.2381 \| 0.03906 \| \| deflection based discrimination \| 0.1431 \| 7 \| 9.52E-03 \| 0.9922 \|  \| 0.02857 \| 0.9766 \|  \| - \| - \|  \| 0.1429 \| 0.4688 \| \| MLE \| 0.1503 \| 7 \| 0.01905 \| 0.9844 \|  \| 0.09524 \| 0.7656 \|  \| 0.2286 \| 0.05469 \|  \| 0.2095 \| 0.1094 \| \| dynamically stable state discrimination \| 0.2062 \| 7 \| 0.01905 \| 0.9688 \|  \| 0.181 \| 0.2344 \|  \| 0.2571 \| 0.01563 \|  \| 0.1905 \| 0.03125 \| \| **^I,C^I** \|  \|  \|  \|  \|  \|  \|  \|  \|  \|  \|  \|  \|  \| \| dynamical discrimination \| 0.3167 \| 15 \| - \| - \|  \| 0.1398 \| 0.402 \|  \| 0.1753 \| 0.1174 \|  \| 0.2581 \| 3.05E-05 \| \| best-fit Ξ based discrimination \| 0.3255 \| 15 \| 0.1183 \| 0.6192 \|  \| - \| - \|  \| 0.1763 \| 0.1147 \|  \| 0.2581 \| 3.05E-05 \| \| deflection based discrimination \| 0.3253 \| 15 \| 0.0828 \| 0.8881 \|  \| 0.08172 \| 0.8961 \|  \| - \| - \|  \| 0.2581 \| 3.05E-05 \| \| MLE \| 0.2954 \| 15 \| 0.07527 \| 0.9243 \|  \| 0.06667 \| 0.9527 \|  \| 0.09032 \| 0.8486 \|  \| 0.2581 \| 3.05E-05 \| \| dynamically stable state discrimination \| 0.35 \| 15 \| 0.1409 \| 0.08435 \|  \| 0.1914 \| 0.0535 \|  \| 0.1989 \| 0.03296 \|  \| 0.2581 \| 3.05E-05 \| \| **^E,C^I** \|  \|  \|  \|  \|  \|  \|  \|  \|  \|  \|  \|  \|  \| \| dynamical discrimination \| 0.2917 \| 15 \| - \| - \|  \| 0.1226 \| 0.5765 \|  \| 0.1591 \| 0.2271 \|  \| 0.2581 \| 3.05E-05 \| \| best-fit Ξ based discrimination \| 0.2917 \| 15 \| 0.1355 \| 0.4452 \|  \| - \| - \|  \| 0.1656 \| 0.1796 \|  \| 0.2581 \| 3.05E-05 \| \| deflection based discrimination \| 0.249 \| 15 \| 0.09892 \| 0.7894 \|  \| 0.09247 \| 0.8349 \|  \| - \| - \|  \| 0.2559 \| 6.10E-05 \| \| MLE \| 0.2281 \| 15 \| 0.08387 \| 0.8853 \|  \| 0.08172 \| 0.8961 \|  \| 0.1247 \| 0.5548 \|  \| 0.2065 \| 0.02063 \| \| dynamically stable state discrimination \| 0.3167 \| 15 \| 0.1527 \| 0.1301 \|  \| 0.1677 \| 0.1651 \|  \| 0.1914 \| 0.0535 \|  \| 0.2581 \| 3.05E-05 \| \| **^R,C^V** \|  \|  \|  \|  \|  \|  \|  \|  \|  \|  \|  \|  \|  \| \| dynamical discrimination \| 0.275 \| 10 \| - \| - \|  \| 0.1714 \| 0.2158 \|  \| 0.1571 \| 0.3125 \|  \| 0.2238 \| 0.02246 \| \| best-fit Ξ based discrimination \| 0.2372 \| 10 \| 0.09048 \| 0.8125 \|  \| - \| - \|  \| 0.1095 \| 0.6875 \|  \| 0.2476 \| 4.88E-03 \| \| deflection based discrimination \| 0.2463 \| 10 \| 0.1048 \| 0.7217 \|  \| 0.1524 \| 0.3477 \|  \| - \| - \|  \| 0.2524 \| 2.93E-03 \| \| MLE \| 0.2455 \| 10 \| 0.1286 \| 0.5391 \|  \| 0.1429 \| 0.4229 \|  \| 0.1286 \| 0.5391 \|  \| 0.2476 \| 4.88E-03 \| \| dynamically stable state discrimination \| 0.2042 \| 10 \| 0.0381 \| 0.9297 \|  \| 0.09524 \| 0.7842 \|  \| 0.08571 \| 0.8389 \|  \| 0.2143 \| 0.04492 \| \| **^I,S^I** \|  \|  \|  \|  \|  \|  \|  \|  \|  \|  \|  \|  \|  \| \| dynamical discrimination \| 0.3625 \| 18 \| - \| - \|  \| 0.1321 \| 0.4661 \|  \| 0.2372 \| 3.36E-04 \|  \| 0.2297 \| 7.63E-06 \| \| best-fit Ξ based discrimination \| 0.3482 \| 18 \| 0.1246 \| 0.5507 \|  \| - \| - \|  \| 0.2267 \| 1.40E-03 \|  \| 0.2538 \| 1.14E-05 \| \| deflection based discrimination \| 0.2605 \| 18 \| 0.01952 \| 0.9997 \|  \| 0.03003 \| 0.9988 \|  \| - \| - \|  \| 0.2387 \| 2.67E-04 \| \| MLE \| 0.2741 \| 18 \| 0.03604 \| 0.9976 \|  \| 0.04655 \| 0.9931 \|  \| 0.1486 \| 0.2899 \|  \| 0.2538 \| 1.14E-05 \| \| dynamically stable state discrimination \| 0.3125 \| 18 \| 0.03979 \| 0.9929 \|  \| 0.05706 \| 0.9829 \|  \| 0.2252 \| 1.68E-03 \|  \| 0.2568 \| 3.81E-06 \| \| **^E,S^I** \|  \|  \|  \|  \|  \|  \|  \|  \|  \|  \|  \|  \|  \| \| dynamical discrimination \| 0.2833 \| 18 \| - \| - \|  \| 0.1922 \| 0.03327 \|  \| 0.2417 \| 1.64E-04 \|  \| 0.2568 \| 3.81E-06 \| \| best-fit Ξ based discrimination \| 0.2709 \| 18 \| 0.06456 \| 0.97 \|  \| - \| - \|  \| 0.2102 \| 7.97E-03 \|  \| 0.2492 \| 3.81E-05 \| \| deflection based discrimination \| 0.1819 \| 18 \| 0.01502 \| 0.9999 \|  \| 0.04655 \| 0.9931 \|  \| - \| - \|  \| 0.1727 \| 0.1061 \| \| MLE \| 0.1927 \| 18 \| 0.01502 \| 0.9999 \|  \| 0.03303 \| 0.9983 \|  \| 0.1547 \| 0.2341 \|  \| 0.1922 \| 0.03327 \| \| dynamically stable state discrimination \| 0.2667 \| 18 \| 0.05631 \| 0.9836 \|  \| 0.1344 \| 0.4367 \|  \| 0.2222 \| 2.35E-03 \|  \| 0.2282 \| 1.53E-05 \| \| **^R,S^V** \|  \|  \|  \|  \|  \|  \|  \|  \|  \|  \|  \|  \|  \| \| dynamical discrimination \| 0.3 \| 5 \| - \| - \|  \| 0.1273 \| 0.5938 \|  \| 0.2364 \| 0.09375 \|  \| 0.2545 \| 0.0625 \| \| best-fit Ξ based discrimination \| 0.3741 \| 5 \| 0.1455 \| 0.5 \|  \| - \| - \|  \| 0.2182 \| 0.1563 \|  \| 0.2545 \| 0.0625 \| \| deflection based discrimination \| 0.2077 \| 5 \| 0.03636 \| 0.9375 \|  \| 0.05455 \| 0.9063 \|  \| - \| - \|  \| 0.2727 \| 0.03125 \| \| MLE \| 0.3239 \| 5 \| 0.1455 \| 0.5 \|  \| 0.1273 \| 0.5938 \|  \| 0.2182 \| 0.1563 \|  \| 0.2364 \| 0.09375 \| \| dynamically stable state discrimination \| 0.2 \| 5 \| 0.07273 \| 0.8438 \|  \| 0.09091 \| 0.7813 \|  \| 0.2182 \| 0.1563 \|  \| 0.2727 \| 0.03125 \| |
| --- | --- | --- | --- | --- | --- | --- | --- | --- | --- | --- | --- | --- | --- | --- | --- | --- | --- | --- | --- | --- | --- | --- | --- | --- | --- | --- | --- | --- | --- | --- | --- | --- | --- | --- | --- | --- | --- | --- | --- | --- | --- | --- | --- | --- | --- | --- | --- | --- | --- | --- | --- | --- | --- | --- | --- | --- | --- | --- | --- | --- | --- | --- | --- | --- | --- | --- | --- | --- | --- | --- | --- | --- | --- | --- | --- | --- | --- | --- | --- | --- | --- | --- | --- | --- | --- | --- | --- | --- | --- | --- | --- | --- | --- | --- | --- | --- | --- | --- | --- | --- | --- | --- | --- | --- | --- | --- | --- | --- | --- | --- | --- | --- | --- | --- | --- | --- | --- | --- | --- | --- | --- | --- | --- | --- | --- | --- | --- | --- | --- | --- | --- | --- | --- | --- | --- | --- | --- | --- | --- | --- | --- | --- | --- | --- | --- | --- | --- | --- | --- | --- | --- | --- | --- | --- | --- | --- | --- | --- | --- | --- | --- | --- | --- | --- | --- | --- | --- | --- | --- | --- | --- | --- | --- | --- | --- | --- | --- | --- | --- | --- | --- | --- | --- | --- | --- | --- | --- | --- | --- | --- | --- | --- | --- | --- | --- | --- | --- | --- | --- | --- | --- | --- | --- | --- | --- | --- | --- | --- | --- | --- | --- | --- | --- | --- | --- | --- | --- | --- | --- | --- | --- | --- | --- | --- | --- | --- | --- | --- | --- | --- | --- | --- | --- | --- | --- | --- | --- | --- | --- | --- | --- | --- | --- | --- | --- | --- | --- | --- | --- | --- | --- | --- | --- | --- | --- | --- | --- | --- | --- | --- | --- | --- | --- | --- | --- | --- | --- | --- | --- | --- | --- | --- | --- | --- | --- | --- | --- | --- | --- | --- | --- | --- | --- | --- | --- | --- | --- | --- | --- | --- | --- | --- | --- | --- | --- | --- | --- | --- | --- | --- | --- | --- | --- | --- | --- | --- | --- | --- | --- | --- | --- | --- | --- | --- | --- | --- | --- | --- | --- | --- | --- | --- | --- | --- | --- | --- | --- | --- | --- | --- | --- | --- | --- | --- | --- | --- | --- | --- | --- | --- | --- | --- | --- | --- | --- | --- | --- | --- | --- | --- | --- | --- | --- | --- | --- | --- | --- | --- | --- | --- | --- | --- | --- | --- | --- | --- | --- | --- | --- | --- | --- | --- | --- | --- | --- | --- | --- | --- | --- | --- | --- | --- | --- | --- | --- | --- | --- | --- | --- | --- | --- | --- | --- | --- | --- | --- | --- | --- | --- | --- | --- | --- | --- | --- | --- | --- | --- | --- | --- | --- | --- | --- | --- | --- | --- | --- | --- | --- | --- | --- | --- | --- | --- | --- | --- | --- | --- | --- | --- | --- | --- | --- | --- | --- | --- | --- | --- | --- | --- | --- | --- | --- | --- | --- | --- | --- | --- | --- | --- | --- | --- | --- | --- | --- | --- | --- | --- | --- | --- | --- | --- | --- | --- | --- | --- | --- | --- | --- | --- | --- | --- | --- | --- | --- | --- | --- | --- | --- | --- | --- | --- | --- | --- | --- | --- | --- | --- | --- | --- | --- | --- | --- | --- | --- | --- | --- | --- | --- | --- | --- | --- | --- | --- | --- | --- | --- | --- | --- | --- | --- | --- | --- | --- | --- | --- | --- | --- | --- | --- | --- | --- | --- | --- | --- | --- | --- | --- | --- | --- | --- | --- | --- | --- | --- | --- | --- | --- | --- | --- | --- | --- | --- | --- | --- | --- | --- | --- | --- | --- | --- | --- | --- | --- | --- | --- | --- | --- | --- | --- | --- | --- | --- | --- | --- | --- | --- | --- | --- | --- | --- | --- | --- | --- | --- | --- | --- | --- | --- | --- | --- | --- | --- | --- | --- | --- | --- | --- | --- | --- | --- | --- | --- | --- | --- | --- | --- | --- | --- | --- | --- | --- | --- | --- | --- | --- | --- | --- | --- | --- | --- | --- | --- | --- | --- | --- | --- | --- | --- | --- | --- | --- | --- | --- | --- | --- | --- | --- | --- | --- | --- | --- | --- | --- | --- | --- | --- | --- | --- | --- | --- | --- | --- | --- | --- | --- | --- | --- | --- | --- | --- | --- | --- | --- | --- | --- | --- | --- | --- | --- | --- | --- | --- | --- | --- | --- | --- | --- | --- | --- | --- | --- | --- | --- | --- | --- | --- | --- | --- | --- | --- | --- | --- | --- | --- | --- | --- | --- | --- | --- | --- | --- | --- | --- | --- | --- | --- | --- | --- | --- | --- | --- | --- | --- | --- | --- | --- | --- | --- | --- | --- | --- | --- | --- | --- | --- | --- | --- | --- | --- | --- | --- | --- | --- | --- | --- | --- | --- | --- | --- | --- | --- | --- | --- | --- | --- | --- | --- | --- | --- | --- | --- | --- | --- | --- | --- | --- | --- | --- | --- | --- | --- | --- | --- | --- | --- | --- | --- | --- | --- | --- | --- | --- | --- | --- | --- | --- | --- | --- | --- | --- | --- | --- | --- | --- | --- | --- | --- | --- | --- | --- | --- | --- | --- | --- | --- | --- | --- | --- | --- | --- | --- | --- | --- | --- | --- | --- | --- | --- |
| **Table S1 \| Summary of results for all algorithms and all categories**. This table supports the claims and data summarized in Figures 3, 5, S2, and S10 by summarizing comparative algorithm performance broken down by data category. Rows are grouped by data category as named in the first column along with algorithm names. The next two columns give the median correct classification rate (CCR) and the number of recordings (N). The next six columns show either the effect size (r_sdf_) or the p-value for a one-tailed Wilcoxon signed rank test of the hypothesis that CCR was greater for the algorithm named on the row than the algorithm named on the column. The last two columns compare to chance performance which was 1/8 for orientation and 1/6 for size or contrast. |

The data categories that we included in table S1 have scientific interest, but by pooling data from related categories we can gain clarity about specific variables. In particular, by pooling across recording types but controlling the stimulus variable we can separate different aspects of population dynamics. For all pooled orientation categories, we get a median correct classification rate (CCR) of CCR=0.2156 (greater than chance by Wilcoxon signed rank: r_sdf_=0.218, p=0.488⨉10^-4^). For contrast we get CCR=0.2875, (r_sdf_=0.2494, p=5.73⨉10^-11^). For size we get CCR=0.3167, (r_sdf_=0.2405, p=2.73⨉10^-12^). This shows that while orientation information is less prominent in V1 dynamics than size or contrast, there is enough to be detected. By contrast, when we try to classify orientation based on deflection, we get CCR=0.1392 (does not pass Wilcoxon signed rank, r_sdf_=0.1453, p=0.1879). This is despite the fact that we do find orientation tuning curves that are well defined on cross-trial averages and that the most and least preferred orientations do evoke reliable deflection responses (see Fig. S1). There is simply enough trial-to-trial variability among the other orientations that deflection does not inform about orientation well, but a dynamical perspective is less susceptible to this variability. Crucially for theories of neural coding we have shown that neurons do have more information than they pass downstream. Therefore, if an experimenter wants to “listen in” on upstream populations they are better off using a model-based approach like dynamical discrimination than by using summative measures like deflection.

We can also categorize the data by recording type. There we see that inhibitory current dynamics lead to CCR=0.3167, excitatory current dynamics lead to CCR=0.2833, and Vm dynamics (spikes removed) lead to CCR=0.275. Interestingly, synaptic inhibition appears to have more stimulus related data about contrast and size than excitation (r_sdf_=0.067, p=0.0279, N=33). This underscores our key result that synaptic excitation has more fine-grained information about orientation than synaptic inhibition, as only synaptic excitation passed a Wilcoxon Signed rank test for distinguishability for chance. However, the dataset is small enough to limit our ability to make distinctions when controlling for both recording type and stimulus variable. When instead of comparing to chance we directly compared ***^E,O^I*** to ***^I,O^I*** , it did not pass the Wilcoxon signed-rank test for distinguishability. Because we have only 7 examples, the variability overwhelmed the relatively large effect size (r_sdf_=0.0875) and reduced confidence in the difference (p=0.1043). It is likely that a larger dataset is required to make definitive judgements about narrow cross-category comparisons such as this using dynamical discrimination alone. Nonetheless comparisons across algorithms and chance are confidently detected. Furthermore, we can include information from other algorithms to support cross-category insights. For example when best-fit Ξ are used as a basis for discrimination we also find that for contrast and size synaptic inhibition leads to better discriminability than excitation (r_sdf_=0.058, p=4.304⨉10^-3^) and the same was found when using deflection based discrimination (r_sdf_=0.058, p=0.0125). The conclusion that inhibition generally has more stimulus information regarding size and contrast seems robust and highlights the uniqueness of the finding that only a dynamical interpretation and only synaptic excitation can reveal fine grained orientation information.

Lastly it is important to check that our spiking proxy (deflection) can function as a stimulus detector in accordance with the role that computational neuroscience theorizes for individual neurons in V1 that exhibit stimulus tuning. The value of tuning of neural firing is often theorized to be the detection of a key type of stimulus, rather than as continuously informing about the properties of stimulus^22^. So, we tested whether our methods could be used to say whether a stimulus is either the preferred stimulus or the least preferred stimulus when presented with one or the other. We used the same Ξ matrices obtained before from hold-one-out out-of-sample testing but retained only those fitted to recordings coinciding with presentations of the most and least preferred stimuli. We retrained and retested random forest classifiers on these Ξ matrices. Next, we did the same for deflection. This a discrimination test that is very natural for deflection, whereas discriminating between relatively fine variations in stimuli is something we expect deflection to be poor at and thus motivated the development of dynamical discrimination. We find that deflection exhibits high variability in cell-to-cell performance, but generally excels. The only category not greater than chance was membrane potential recordings coinciding with variations in size, which had only five cells, leading to poor statistical resolution despite a median CCR of 0.7. The data supporting claims of significance in Fig. S1 are presented in table S2. These findings confirm that our definition of deflection is a valid basis of comparison. Thus, our extension to finer levels of comparison with dynamical discrimination are useful. Dynamical discrimination had a lower upper limit on performance. Like deflection-based discrimination it had only one category fail to be distinguishable from chance: inhibitory current recordings coinciding with variations in orientation. This was also the worst performing category in our general findings. Deflection out performs dynamical discrimination at binary discrimination tasks, but this not surprising given it is a natural challenge for deflection and the Ξ matrices we use for dynamical discrimination were extensively optimized for a different task (fine distinctions rather than broad ones). Finally, the usefulness of deflection for binary classification underscores the insight that neurons receive more information than they pass on to other brain regions.

| **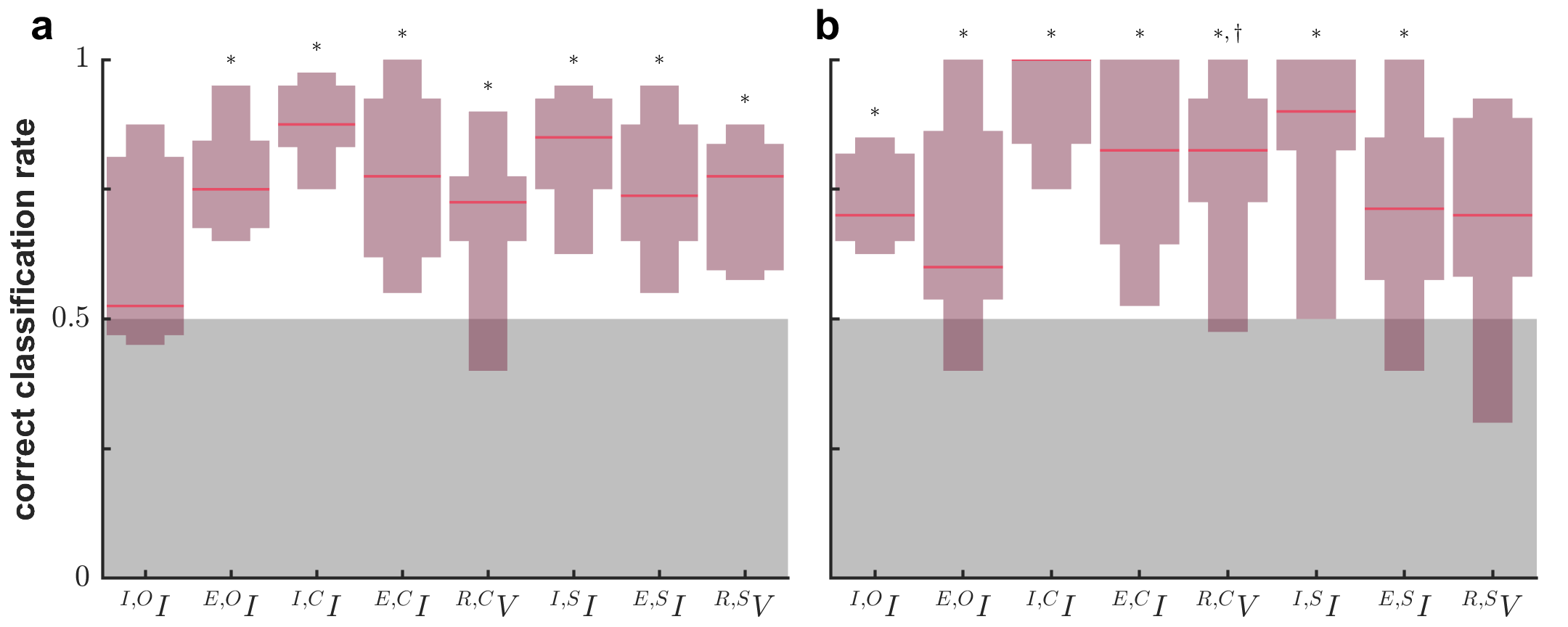** |
| --- |
| **Figure S1 \| Deflection is useful as a binary discriminator in accordance with stimulus selectivity theory. a,** Same as Fig. 3b, except showing the classification ability of using dynamical discrimination when limiting to just the data from each cell’s least and most preferred stimulus. Distinguishability from chance according to the Wilcoxon signed-rank test is indicated with *. **b,** Same as Fig. 3a except showing classification ability of using deflection when limiting to just the data from each cell’s least and most preferred stimulus. Distinguishability from chance is indicated with *, and from dynamical discrimination with †. |

| \|  \|  \| **One-tailed Wilcoxon signed rank test that CCR is greater than that of…** \| \| \| \| \| \| --- \| --- \| --- \| --- \| --- \| --- \| --- \| \|  \|  \| **the other algorithm** \| \|  \| **chance** \| \| \| **Algorithm and data groups** \| **median CCR** \| **r_sdf_** \| **p-value** \|  \| **r_sdf_** \| **p-value** \| \| **All data groups pooled** \|  \|  \|  \|  \|  \|  \| \| dynamical discrimination \| 0.7875 \| 0.0913 \| 0.9740 \|  \| 0.2500 \| 1.2E-30 \| \| deflection based discrimination \| 0.825 \| 0.1419 \| 0.0261 \|  \| 0.2432 \| 1.6E-27 \| \| **^I,O^I** \|  \|  \|  \|  \|  \|  \| \| dynamical discrimination \| 0.525 \| 0.0762 \| 0.8516 \|  \| 0.2000 \| 0.14063 \| \| deflection based discrimination \| 0.7 \| 0.1905 \| 0.1875 \|  \| 0.2667 \| 0.00781 \| \| **^E,O^I** \|  \|  \|  \|  \|  \|  \| \| dynamical discrimination \| 0.75 \| 0.1952 \| 0.1641 \|  \| 0.2667 \| 0.00781 \| \| deflection based discrimination \| 0.6 \| 0.0714 \| 0.8672 \|  \| 0.2333 \| 0.04688 \| \| **^I,C^I** \|  \|  \|  \|  \|  \|  \| \| dynamical discrimination \| 0.875 \| 0.0581 \| 0.9460 \|  \| 0.2581 \| 3.1E-05 \| \| deflection based discrimination \| 1 \| 0.1677 \| 0.0568 \|  \| 0.2581 \| 3.1E-05 \| \| **^E,C^I** \|  \|  \|  \|  \|  \|  \| \| dynamical discrimination \| 0.775 \| 0.1290 \| 0.5052 \|  \| 0.2581 \| 3.1E-05 \| \| deflection based discrimination \| 0.825 \| 0.1290 \| 0.5052 \|  \| 0.2581 \| 3.1E-05 \| \| **^R,C^V** \|  \|  \|  \|  \|  \|  \| \| dynamical discrimination \| 0.725 \| 0.0143 \| 0.9961 \|  \| 0.2524 \| 0.00293 \| \| deflection based discrimination \| 0.825 \| 0.2000 \| 0.0098 \|  \| 0.2571 \| 0.00195 \| \| **^I,S^I** \|  \|  \|  \|  \|  \|  \| \| dynamical discrimination \| 0.85 \| 0.0743 \| 0.9425 \|  \| 0.2568 \| 3.8E-06 \| \| deflection based discrimination \| 0.9 \| 0.1824 \| 0.0607 \|  \| 0.2297 \| 7.6E-06 \| \| **^E,S^I** \|  \|  \|  \|  \|  \|  \| \| dynamical discrimination \| 0.7375 \| 0.1141 \| 0.5138 \|  \| 0.2568 \| 3.8E-06 \| \| deflection based discrimination \| 0.7125 \| 0.1156 \| 0.4954 \|  \| 0.2485 \| 3.8E-05 \| \| **^R,S^V** \|  \|  \|  \|  \|  \|  \| \| dynamical discrimination \| 0.775 \| 0.1727 \| 0.3438 \|  \| 0.2727 \| 0.03125 \| \| deflection based discrimination \| 0.7 \| 0.1000 \| 0.7188 \|  \| 0.2273 \| 0.125 \| |
| --- | --- | --- | --- | --- | --- | --- | --- | --- | --- | --- | --- | --- | --- | --- | --- | --- | --- | --- | --- | --- | --- | --- | --- | --- | --- | --- | --- | --- | --- | --- | --- | --- | --- | --- | --- | --- | --- | --- | --- | --- | --- | --- | --- | --- | --- | --- | --- | --- | --- | --- | --- | --- | --- | --- | --- | --- | --- | --- | --- | --- | --- | --- | --- | --- | --- | --- | --- | --- | --- | --- | --- | --- | --- | --- | --- | --- | --- | --- | --- | --- | --- | --- | --- | --- | --- | --- | --- | --- | --- | --- | --- | --- | --- | --- | --- | --- | --- | --- | --- | --- | --- | --- | --- | --- | --- | --- | --- | --- | --- | --- | --- | --- | --- | --- | --- | --- | --- | --- | --- | --- | --- | --- | --- | --- | --- | --- | --- | --- | --- | --- | --- | --- | --- | --- | --- | --- | --- | --- | --- | --- | --- | --- | --- | --- | --- | --- | --- | --- | --- | --- | --- | --- | --- | --- | --- | --- | --- | --- | --- | --- | --- | --- | --- | --- | --- | --- | --- | --- | --- | --- | --- | --- | --- | --- | --- | --- | --- | --- | --- | --- | --- | --- | --- | --- | --- | --- | --- | --- | --- | --- | --- | --- | --- | --- | --- | --- | --- | --- | --- | --- | --- | --- | --- | --- | --- | --- | --- | --- | --- | --- |
| **Table S2 \| binary discrimination data.** This table gives the exact test values summarized in Fig. S1. It is the same as table S1 except that only two algorithms are compared. Rows are grouped by data category, indicated in the first column along with the algorithm names. The second column contains the median correct classification rate (CCR). For the next two columns the Wilcoxon signed rank effect size (r_sdf_ and p-value) shown are for a test that the algorithm identified on the row outperformed the other algorithms in the same category. For example, the All - dynamical discrimination row in the r_sdf_ column is the effect size for a comparison to All - deflection based discrimination. The last two columns show a comparison to chance (CCR=1/2). |

| 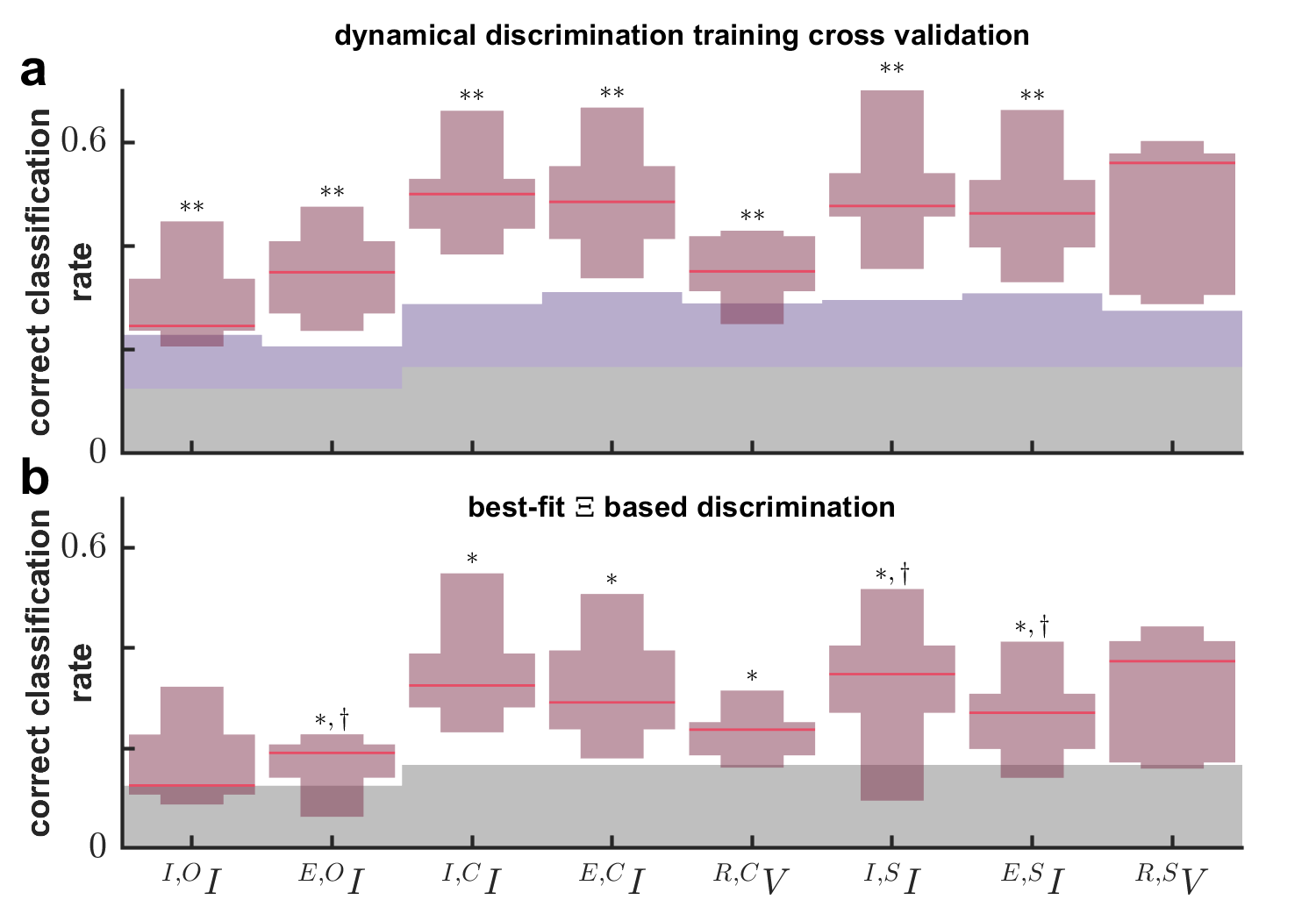 |
| --- |
| **Figure S2 \| Best-fit Ξ based discrimination performs well and has a lower tendency to overfit.** **a,** Same as Fig. 3b, except showing the last generation cross-validation performance of Ξ matrices chosen to optimize discrimination (out-of-sample generalization is panel d). Lavender shading shows overfitting effect (random surrogate median CCR) and is barely visible in Fig. 3a and Fig. S2b as they are at or below chance. All categories (even surrogates) are distinguished from chance. ** indicates distinguishability from random surrogates. **b**, Overfitting vanishes while permitting better than deflection discrimination when Ξ is chosen to optimize ODE modelling. Distinguishability from chance and deflection are indicated with * and † respectively. |

# S.2 Hyperparameter optimization reveals epoch dependence of dynamical discrimination

Dynamical discrimination based on genetic SINDy requires choosing a large number of hyperparameters. This is discussed in methods. Some of these choices are of scientific merit. For example, it may seem intuitive that the steady state of stimulus response would better represent dynamics because it avoids “transients” and would therefore permit better dynamical discrimination. However, we found that a brief period at the beginning of the stimulus response allowed the highest levels of dynamical discrimination, perhaps supporting the findings of fast attractor dynamics in vision^23^ and belying the implication of calling these periods “transients” ^5,24,25^. Additionally, it is important from the standpoint of rigor and transparency that all publications which make extensive use of machine learning should report on the variations they tried and demonstrate that they optimized for a parameter that is independent of the scientific comparisons they see. Figures S3-S7 show classification performance in various scenarios, including whether or not to use an ensemble of ^B^Ξ matrices (see methods) for classification, the regularization factor which influences how many non-zero elements there are in the ^B^Ξ examples, how many dimensions to include in the dimensionality expansion, whether to use second order differential equations, and finally what stimulus presentation epoch to use for dynamical discrimination. The data are from the cross-validation set where overfitting is also observed. However, overfitting is stable across categories, not contributing greatly to the variability (Fig. S2a). The final holdout performance is lower but large differences in performance are preserved.

Discriminability is an experimental variable familiar to neuroscience which attempts to characterize the ability to classify a stimulus given a neural response and a theory about how information is encoded in it. It has been found that discriminability is highest at the early stages of a neural response, the on-epoch^25^. Our analysis also shows that discriminability is higher in the on-epoch versus the “off-epoch”, assuming our hypothesis about stimulus information modulating parameters of the governing dynamical system. This is true for all stimulus types and signal types. Importantly we also examined the epoch dependence of deflection and found that classification based on deflection is completely ineffective for the “off-epoch”. The fact that this analysis reproduces known details of neural computation lends credibility to the method and is novel in the sense that this has never been demonstrated at the single neuron level.

We also see that additional dimensions and second order derivatives are generally not required. We believe that this is because the first three dimensions represent mixtures of variables, not single variables. This was confirmed by running FitzHugh-Nagumo^26^ single neuron simulations driven with sine-wave current injection. The original dynamics have four dimensions, but dimensionality expansion did produce two dimensions corresponding to FitzHugh-Nagumo and another two dimensions corresponding to a sine wave. Consider the possibility that there are multiple sets of subpopulations in upstream neurons and each set is independent of the other sets and each set has its own unique dynamics. Our dimensionality expansion method (time delay and SVD) is not guaranteed to separate them such that each dimension corresponds to only one set of subpopulations. Therefore, each dimension may represent a mixture of variables from independent systems. Thus, dimensionality expansion would give a maximally compact representation and a small number of dimensions would be needed, in our case three. In some cases, the higher dimension systems allow some improvement in classification, but not a large enough difference to be confident that it would carry into final holdout performance, or to justify the additional computational time required.

Lastly, we also test the dimensionality reduction algorithm tried, SVD prioritizes orthogonality between the components it estimates. We tested it against independent component analysis (ICA) which prioritizes statistical independence instead. It was found that ICA did not perform better, though it did produce visually distinct trajectories with much faster dynamics.

| **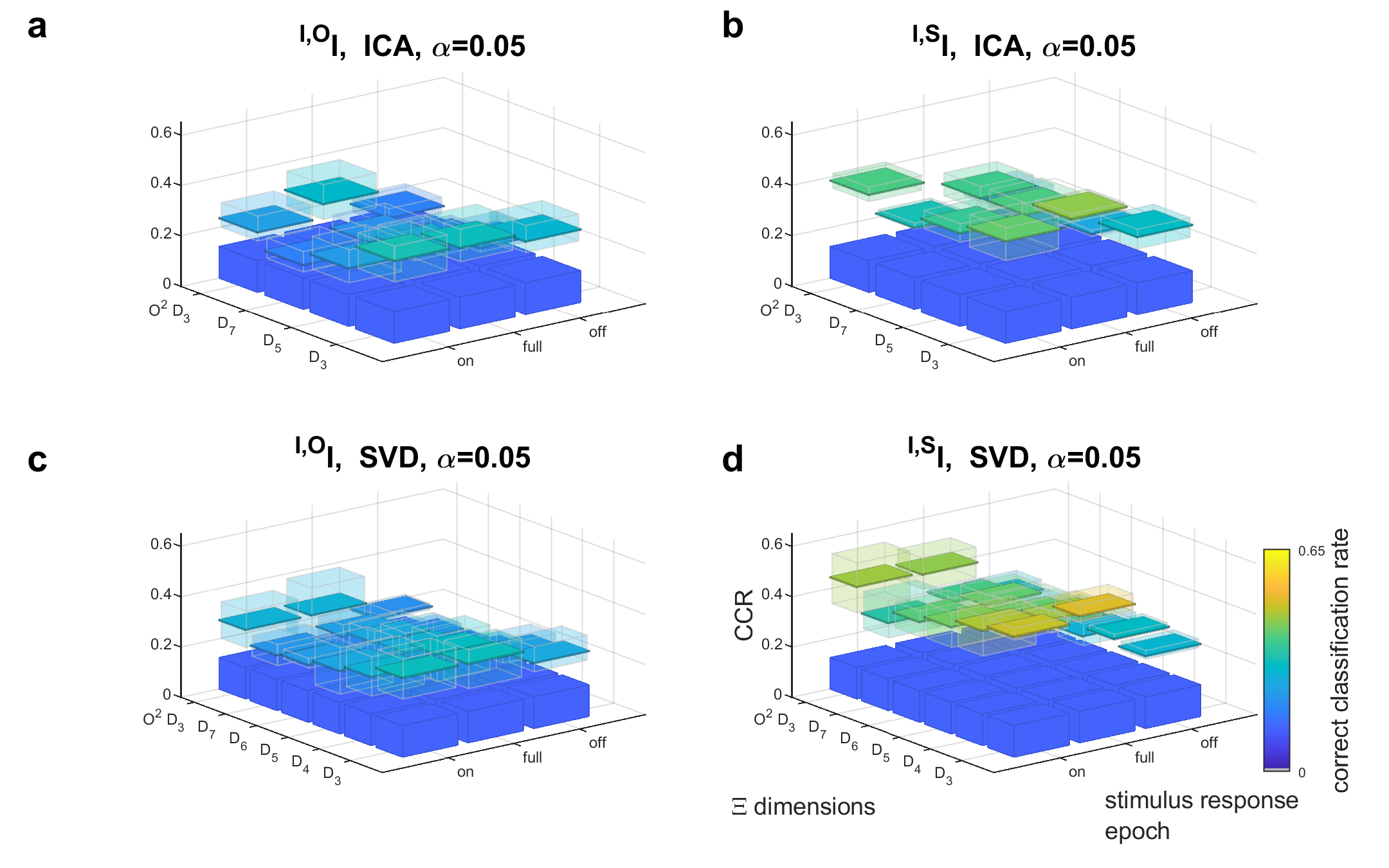** |
| --- |
| **Figure S3 \| Comparisons for hyperparameter optimization part one: weak regularization, SVD compared with ICA along with epoch and dimension options.** Four 3D bar charts showing the effect of several hyperparameters are plotted in a grid. It is evident that the off epoch is least informative and SVD outperforms ICA. **a,** Synaptic inhibition while orientation was varied was tested in combination with ICA based time-delay dimensionality expansion and a weak sparseness regularization. The vertical axis is the correct classification rate, the color also indicates the correct classification rate to aid visual comparison. Fully colored planes show median values. Translucent boxes show the variability (the min and max of the cells tested for that hyperparameter combination). The solid colored boxes show the rate of correct classification by chance. The horizontal axes are labeled, giving the hyperparameters tested for each bar position. There are three stimulus epochs and varying options for the dimensions (columns of Ξ) to include when fitting. The subscript of D denotes how many first order dimensions are kept “O^2^” denotes that the fitted ODE model included second order derivatives, hence O^2^ D_3_ corresponds to a Ξ matrix with six columns. For each combination of hyperparameters the three cells with the greatest number of trials were tested. **b,** The same as in a except that synaptic inhibition was recorded while size varied. **c,** The same as in a except that SVD was used for time-delay based dimensionality expansion. **d,** The same as in c except that synaptic inhibition was recorded while size varied. |

| **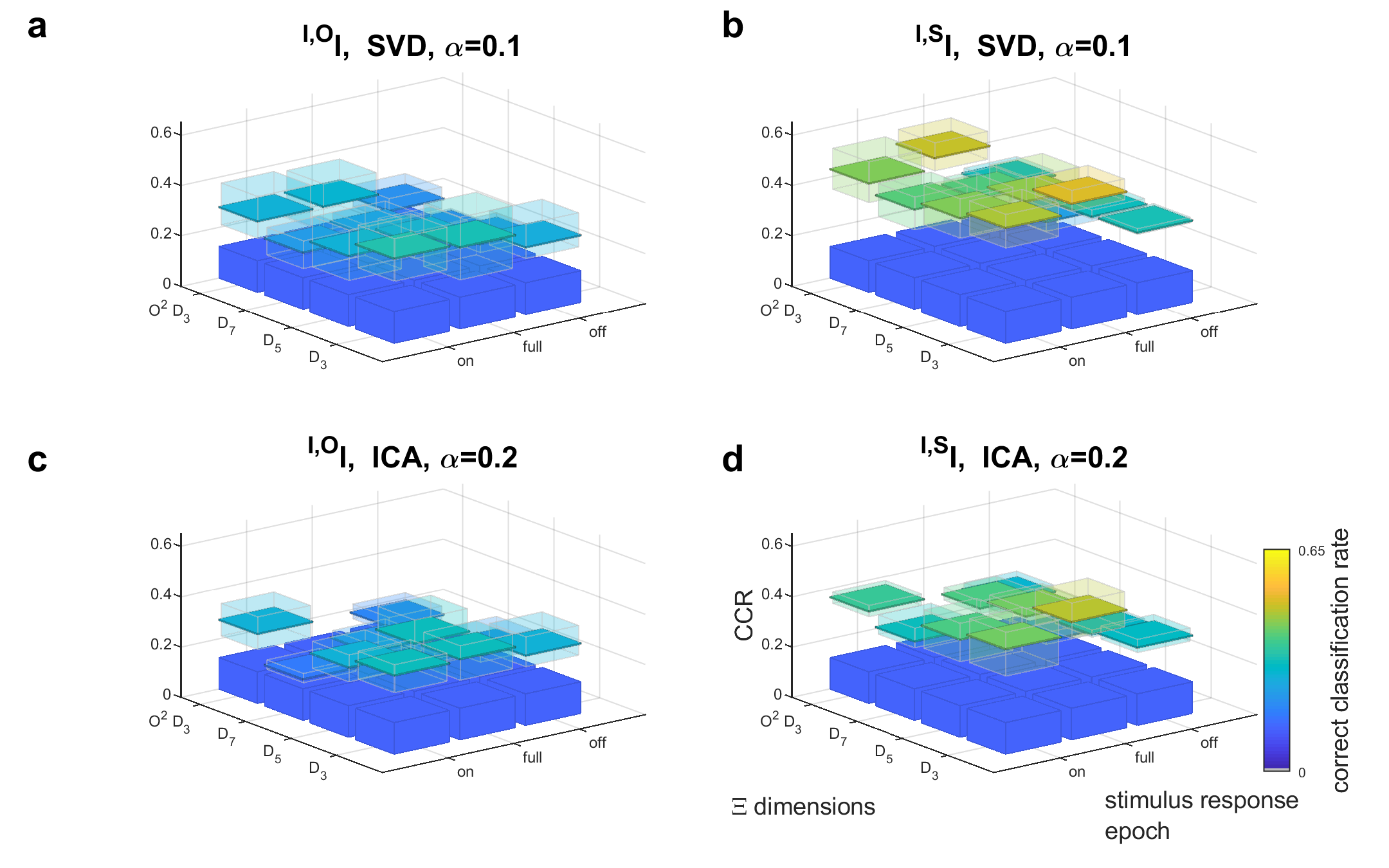** |
| --- |
| **Figure S4 \| Comparisons for hyperparameter optimization part two: varied regularization and dimensionality reduction along with epoch and dimension options.** Four 3D bar charts showing the effect of several hyperparameters are plotted in a grid. **a,** Synaptic inhibition while orientation was varied was tested in combination with SVD based time-delay dimensionality expansion and a weak sparseness regularization. The vertical axis is the correct classification rate, the color also indicates the correct classification rate to aid visual comparison. Fully colored planes show median values. Translucent boxes show the variability (the min and max of the cells tested for that hyperparameter combination). The solid colored boxes show the rate of correct classification by chance. The horizontal axes are labeled, giving the hyperparameters tested for each bar position. There are three stimulus epochs and varying options for the dimensions (columns of Ξ) to include when fitting. The subscript of D denotes how many first order dimensions are kept, while “O^2^” denotes that the fitted ODE model included second order derivatives, hence O^2^ D_3_ corresponds to a Ξ matrix with six columns. For each combination of hyperparameters the three cells with the greatest number of trials were tested. Comparing to Fig. S3c, and S5a reveals that increased sparseness regularization continues to improve performance. **b,** The same as in a except that synaptic inhibition was recorded while size varied. Comparing to Fig. S3b, and S5b reveals that increased sparseness regularization continues to improve performance. **c,** The same as in a except that ICA was used for time-delay based dimensionality expansion, and sparseness regularization is moderate. Comparison with Fig. S5a shows that ICA continues to underperform SVD even with higher sparseness regularization. **d,** The same as in c except that synaptic inhibition was recorded while size varied. Comparison with Fig. S6b shows that ICA continues to underperform SVD even with higher sparseness regularization. |

| **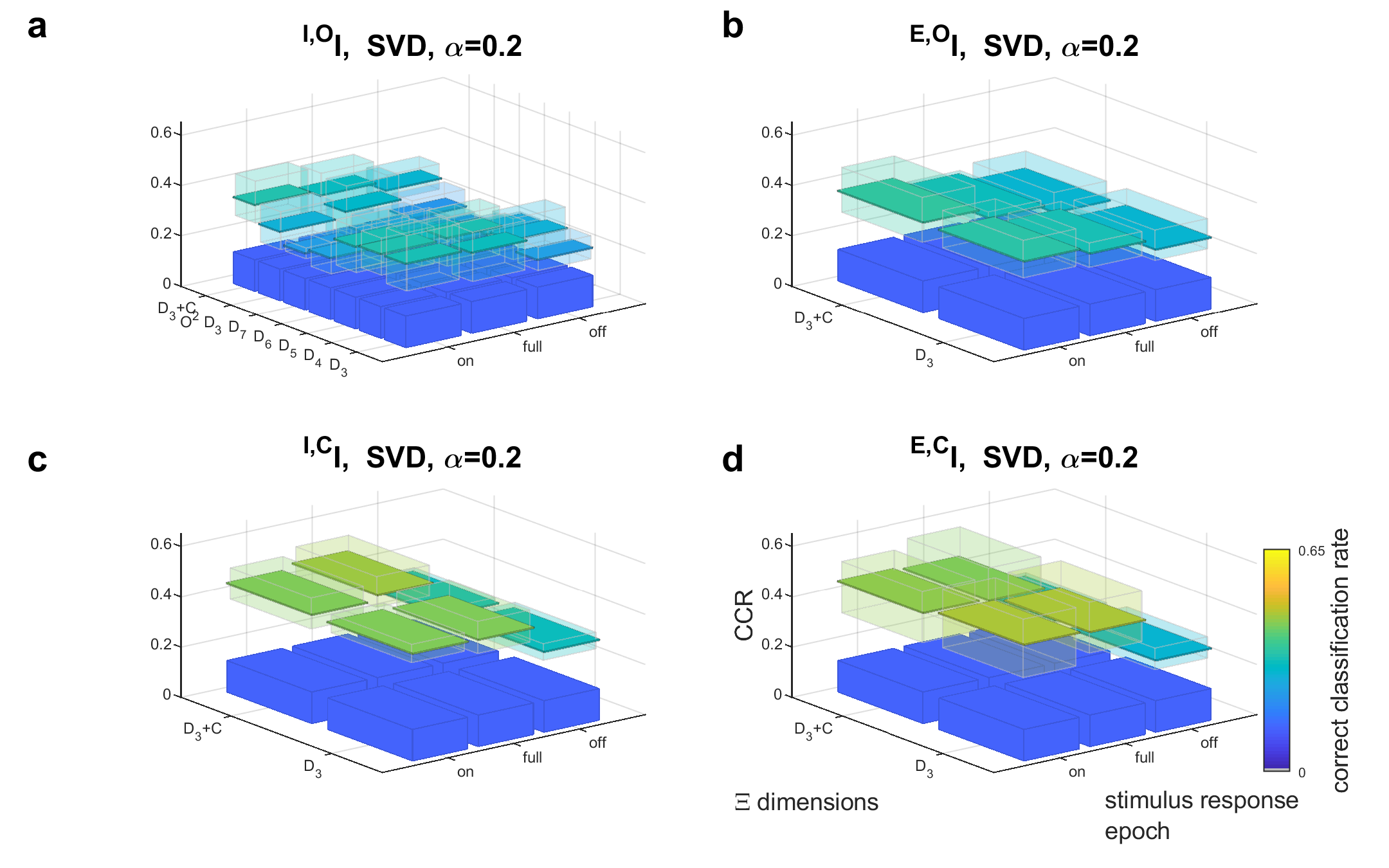** |
| --- |
| **Figure S5 \| Comparisons for hyperparameter optimization part 3: moderate regularization, four categories showing epoch and dimension dependence.** Four 3D bar charts showing the effect of several hyperparameters are plotted in a grid. **a,** Synaptic inhibition while orientation was varied was tested in combination with SVD based time-delay dimensionality expansion and a moderate sparseness regularization. The vertical axis is the correct classification rate, the color also indicates the correct classification rate to aid visual comparison. Fully colored planes show median values. Translucent boxes show the variability (the min and max of the cells tested for that hyperparameter combination). The solid colored boxes show the rate of correct classification by chance. The horizontal axes are labeled, giving the hyperparameters tested for each bar position. There are three stimulus epochs and varying options for the dimensions (columns of Ξ) to include when fitting. The subscript of D denotes how many first order dimensions are kept, while “O^2^” denotes that the fitted ODE model included second order derivatives, hence O^2^ D_3_ corresponds to a Ξ matrix with six columns, and “+C” denotes that the fitted ODE model included a quenched noise term (four column Ξ). For each combination of hyperparameters the three cells with the greatest number of trials were tested. The point D_3_ & “on” was chosen for final analysis. The colormap evinces that no other dimension and epoch choices performed significantly better given the variability. The “off” epoch performed poorly. Comparison with Fig. S7a and S7c shows that sparseness regularization has plateaued. **b,** The same as in a except that synaptic excitation was recorded while orientation varied, and only two-dimension options are tested. We can see that the inclusion of a driving noise term did not enable significantly better stimulus discriminability given the variability. **c,** The same as in b except that synaptic inhibition was recorded while contrast varied. **d,** The same as in b except that synaptic excitation was recorded while contrast varied. |

| **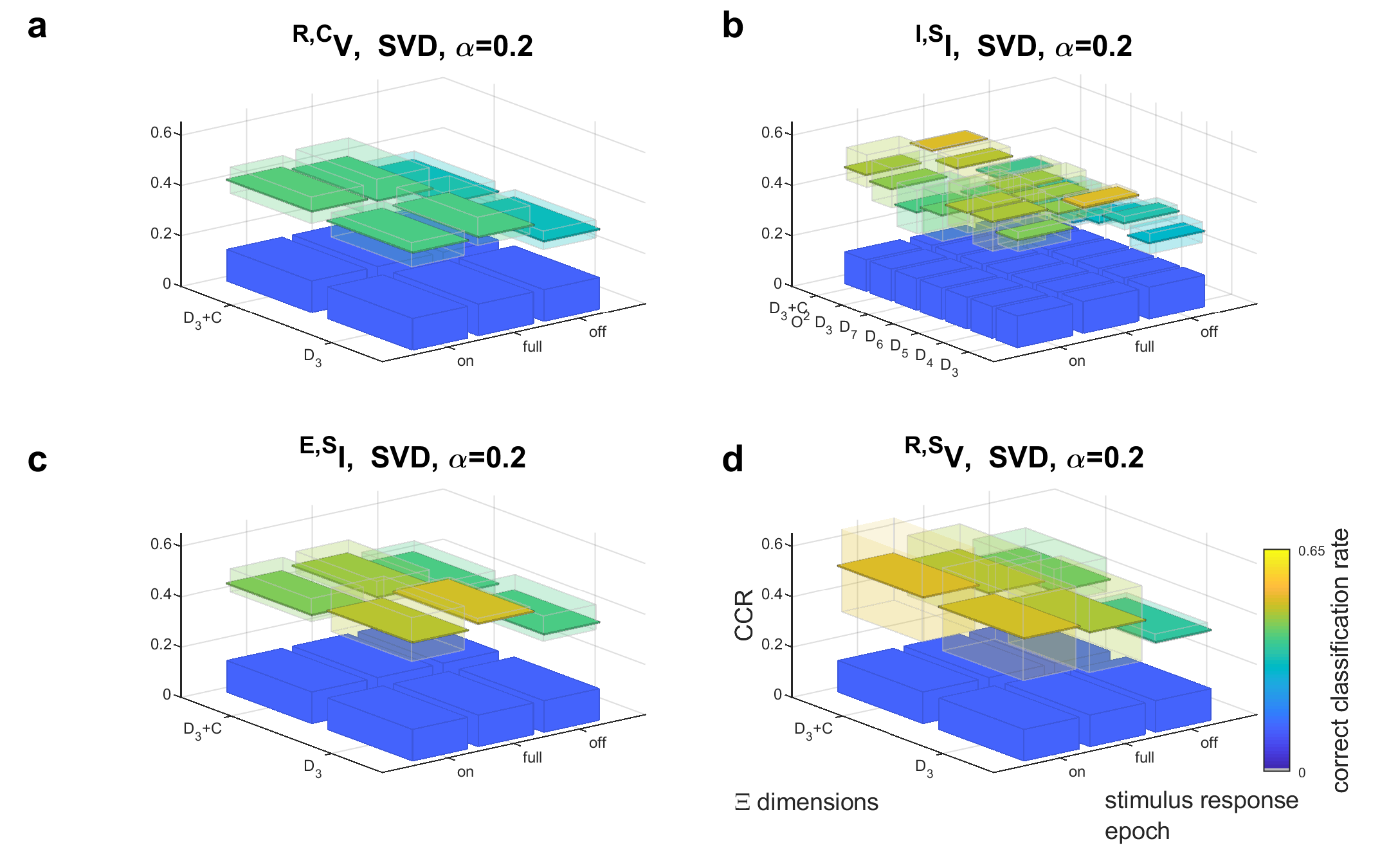** |
| --- |
| **Figure S6 \| Comparisons for hyperparameter optimization part four: moderate regularization, four categories showing epoch and dimension dependence.** Four 3D bar charts showing the effect of several hyperparameters are plotted in a grid. **a,** Membrane potential while contrast was varied was recorded and spikes were removed. The vertical axis is the correct classification rate, the color also indicates the correct classification rate to aid visual comparison. Fully colored planes show median values. Translucent boxes show the variability (the min and max of the cells tested for that hyperparameter combination). The solid colored boxes show the rate of correct classification by chance. The horizontal axes are labeled, giving the hyperparameters tested for each bar position. There are three stimulus epochs and varying options for the dimensions (columns of Ξ) to include when fitting. The subscript of D denotes how many first order dimensions are kept, while “+C” denotes that the fitted ODE model included a quenched noise term (four column Ξ). For each combination of hyperparameters the three cells with the greatest number of trials were tested. We can see that the inclusion of a noise term did not significantly improve classification given the variability. The point D_3_ & “on” was chosen for final analysis. **b,** The same as in a except that synaptic inhibition was recorded while size varied, and many more dimension options were tested. The axis mark “O^2^” denotes that the fitted ODE model included second order derivatives, hence O^2^ D_3_ corresponds to a Ξ matrix with six columns. The point D_3_ & “on” was chosen for final analysis. The colormap evinces that no other dimension choices performed better given the variability. The “off” epoch performed poorly, but the “full” epoch performed well. Comparing to the other plots including those on Fig. S5 shows that the “full” response epoch did not generally perform better given the variability. Comparison with Fig. S7b and S7d shows that sparseness regularization has plateaued. **c,** The same as in a except that synaptic excitation was recorded while size varied. **d,** The same as in a except that membrane potential while size was varied was recorded and spikes were removed. |

| **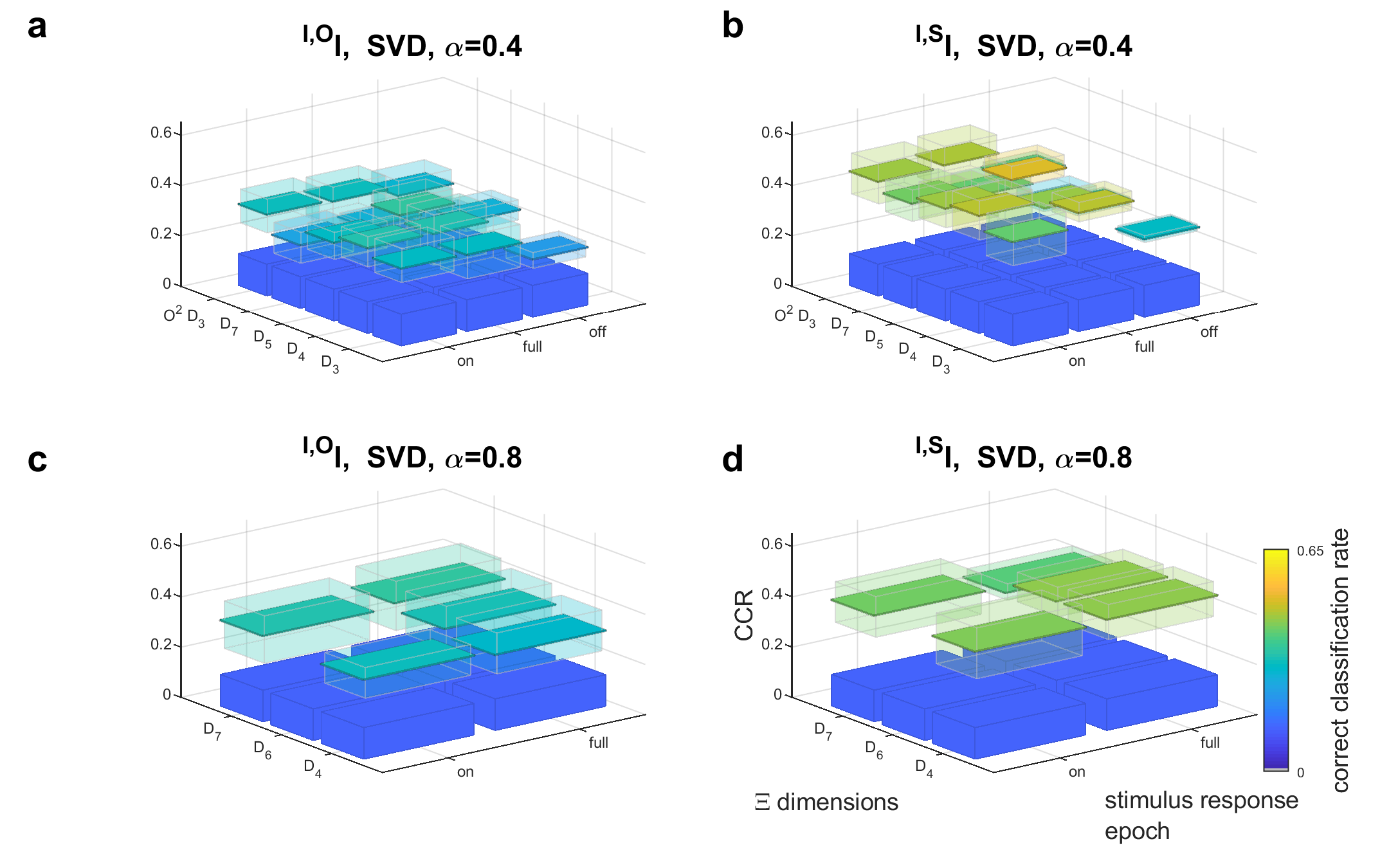** |
| --- |
| **Figure S7 \| Comparisons for hyperparameter optimization part five, varied strong regularization along with epoch and dimension options.** Four 3D bar charts showing the effect of several hyperparameters are plotted in a grid. **a,** Synaptic inhibition while orientation was varied was tested in combination with SVD based time-delay dimensionality expansion and strong sparseness regularization. The vertical axis is the correct classification rate, the color also indicates the correct classification rate to aid visual comparison. Fully colored planes show median values. Translucent boxes show the variability (the min and max of the cells tested for that hyperparameter combination). The solid colored boxes show the rate of correct classification by chance. The horizontal axes are labeled, giving the hyperparameters tested for each bar position. There are three stimulus epochs and varying options for the dimensions (columns of Ξ) to include when fitting. The subscript of D denotes how many first order dimensions are kept “O^2^” denotes that the fitted ODE model included second order derivatives, hence O^2^ D_3_ corresponds to a Ξ matrix with six columns. For each combination of hyperparameters the three cells with the greatest number of trials were tested. Through comparison to Fig. S3c, S4a, S5a, and panel c here, we see that the impact of sparseness regularization is minimal beyond about α=0.2. **b,** The same as in a except that synaptic inhibition was recorded while size varied. Through comparison to Fig. S3d, S4b, S5b, and panel d here, we see that the impact of sparseness regularization is minimal beyond about α=0.2. **c,** The same as in a except that sparseness regularization is now α=0.8. Data was not collected for D_6_ and “on”, or any “off” epochs. **d,** The same as in c except that synaptic inhibition was recorded while size varied. |

# S.3 Analysis of dynamical stability underscores an attractor dynamics interpretation

| 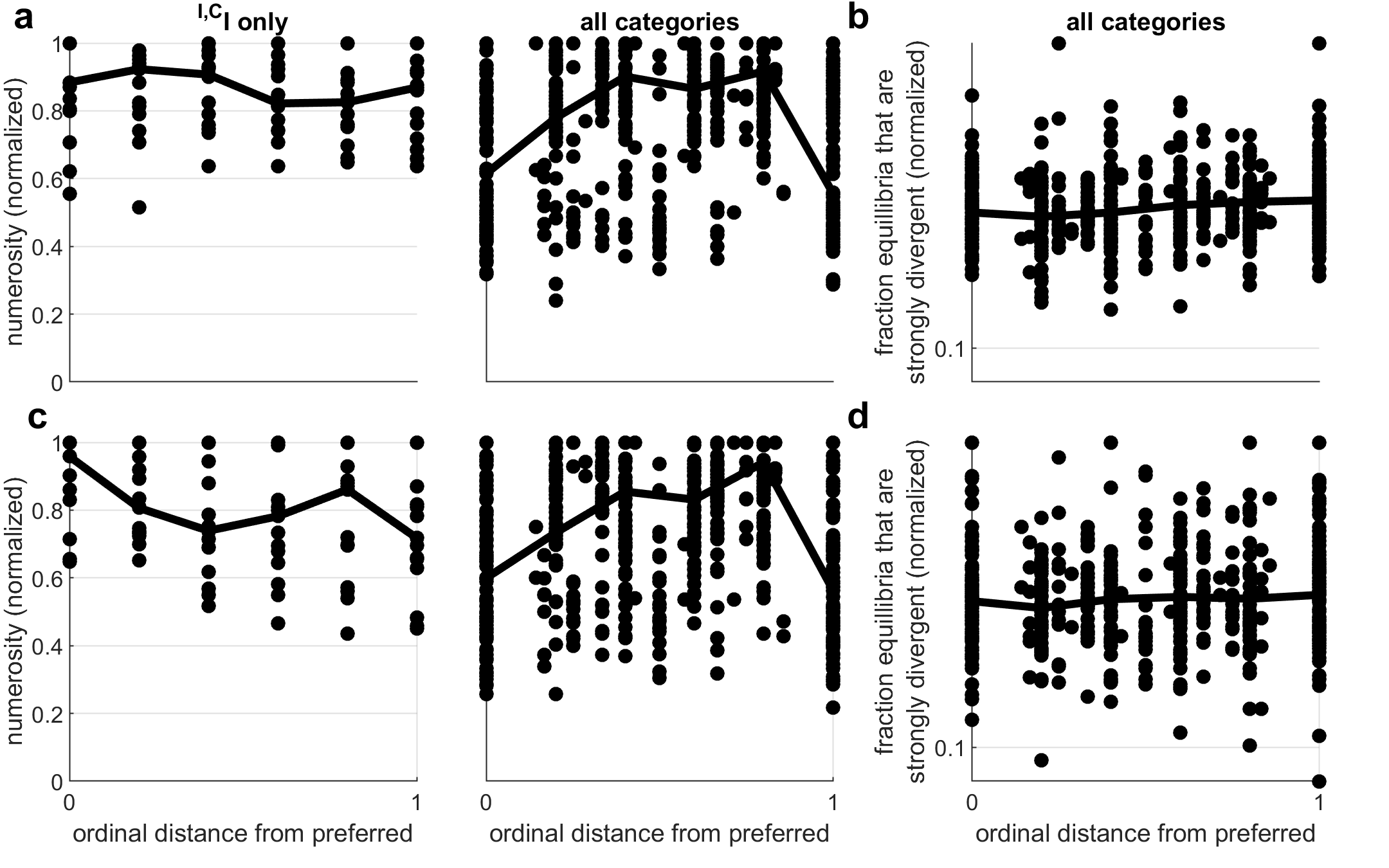 |
| --- |
| **Figure S8 \| Scatter plots summarizing differences between stimulus conditions according to various indicators of bifurcations.** **a,** The first row of plots (a and b) shows data from analyzing the fitted ODE which modeled the dynamics the best (best-fit Ξ). The horizontal axis is the ordinal distance from the most preferred stimulus. The vertical axis plots fixed point numerosity. The solid black line shows the median of 6 deciles of ordinal distance. The left column shows a single category **^I,C^I**, which is not U-shaped, and no significant trend is present. The right column shows the result of pooling all the data, a U-shaped trend is visible but upon further scrutiny it is due to combining data from cells with opposite trends, not a fundamental U-shaped trend for individual cells. **b,** Data from the Ξ matrices (ODE models) that best describe the trajectories are plotted. The vertical axis plots the fraction of fixed points (equilibria) whose L^2^ norm of negative real-valued parts was smaller than the L^2^ norm of positive real-valued parts. A trend is present but weak. **c,** Same as in a except showing results from the fitted ODE that permitted best classification (dynamical discrimination). A significant trend is present for the data from **^I,C^I**. Many cells had the opposite trend (not always significant). Consequentially pooling the data (plotted on the right) shows an inverted U. After aligning cells with opposite trends an overall trend is significant. **d,** Same as in b except showing results from the fitted ODE that permitted best classification (dynamical discrimination). No trend is detected. |

Figure S8 shows the results of analysis of Ξ matrices pursuant to stability and bifurcation analysis from nonlinear dynamics. Real valued fixed points are found by solving for them (equating columns of Ξ to zero) with MATLAB’s symsolve computer algebra system. There is no guarantee that any real-valued fixed points exist, as the system may be overdetermined or underdetermined. However, this is rare with these data. The best-fit Ξ matrices yield real-valued fixed points for 99.2% of recordings, and the Ξ matrices from dynamical discrimination yield real-valued fixed points for 87.8% of recordings. In Figure S8, (left and center columns) we see that the number of real-valued fixed points varies based on the stimulus coinciding with the data Ξ was fitted to. If the number of fixed points change when varying Ξ parameters, then a bifurcation is identified. Thus, we have additional evidence for distinct stimulus-evoked dynamics. The category of inhibitory current recordings co-occurring with varied contrast shows the most consistent cell-to-cell pattern. There is a trend toward fewer fixed points as the presented stimuli gets further from the preferred stimuli when looking at ODEs that permit the best classification performance (Spearman correlation r=-0.2372, p=0.0244, Fig. S8c), but not for ODEs that best model dynamics (r=-0.1520, p=0.1528, Fig. S8a).

We also find differences in the convergence/divergence of behavior near fixed points. When linearized near a fixed point (where derivatives are very small) the behavior of the maximum eigenvalue governs the stability of the dynamics near that fixed point. If multiple fixed points exist, we linearize around each one. If the real value of the maximum eigenvalue passes through zero when changing Ξ parameters, then a different kind of bifurcation in the dynamics is identified. We plot a related concept “net convergence”, in the right most panel of Figure S8 and show that it weakly depends on Ξ matrices. If the three eigenvalues of the Jacobian-linearization at each fixed point have both positive and negative real components and the L^2^ norm of the negative real-valued components is larger than the L^2^ norm of the positive real-valued components then the fixed point is “net-convergent”. The slight trend is that the fraction of fixed points that are net divergent tends to increase for stimuli a larger ordinal distance from the preferred stimulus. It is not necessary to control cell-to-cell variability to observe this effect. It is most detectable for best fit ODEs (Spearman Correlation, r=0.1093, p=0.0075) and not significant for ODEs that permit the best classification (r=0.0717, p=0.0800). This weak trend is double checked by performing the Wilcoxon rank-sum test to compare the median fractions of the data above and below the 25th and 75th percentiles of normalized ordinal distance to preferred stimulus respectively, (r_sdf_=0.1506, p=0.0049 for best fit ODEs and r_sdf_=0.1015, p=0.0419 for ODEs that permit the best classification).

An important consideration for attempting to identify bifurcations by using linear stability analysis is cell-to-cell variability. While one cell may have more fixed points for the least preferred stimuli than for the most preferred there is no a priori reason why a different cell cannot show the opposite trend, or even have the same number of fixed points for the most and least preferred stimuli but a different number for the intermediate stimuli. This effect is seen in our data and makes it difficult to gather population statistics. Consider the U-shaped trend in the central column of Figure S8 (S8a and S8c). In the first case the U-shape trend exists for individual cells as evinced by a high correlation between reliability and distance from the most or least preferred stimulus (which takes the plot and “folds it vertically” at an individual level). That correlation showed that there is a non-monotonic trend. There is no such correlation for N. When all data from all cells are normalized without aligning trends and plotted against the normalized ordinal distance from only the preferred stimulus there is an inverted U appearance. This inverted U-shaped trend is not a coincidence, as revealed by affecting the following change of variables on the horizontal axis $x=|x-0.5|$ and then obtaining the Spearman correlation (r=-0.2414, p=2.248⨉10^-9^ for best fit ODEs and r=-0.2171, p=8.258⨉10^-8^ for ODEs that permit the best classification). This is similar to but not the same as the ordinal distance from either the most or least preferred stimulus. Nonetheless this inverted U-shape is due to the fact that some cells have a positive trend while others show a negative trend and not because cells individually have an inverted U-shaped trend. The existence of two strong but opposite trends among different cells is shown to be the cause of the inverted U-shaped trend via the following analysis. For each cell we obtain the average number of fixed points for the ODEs fitted to each stimulus, denoted by $N_{i,j}$ where i denotes the cell index and j denotes the stimulus index. Then we subtract out the mean, multiply by the sign of the value at the preferred stimulus (which aligns the trends) and add the mean back,${N'}_{i,j}=(N_{i,p}/|N_{i,p}|)\cdot(N_{i,j}-\langle N_{i,j}\rangle_{j})+\langle N_{i,j}\rangle_{j}$, where p denotes the index of the preferred stimulus. Finally, we normalize the largest value to one ${N''}_{i,j}={N'}_{i,j}/\max_{j}({N'}_{i,j})$. Then we measure the correlation of these aligned trends and find a Spearman correlation of (r=-0.1907, p=2.658⨉10^-6^ for best fit ODEs and r=-0.2249, p=2.721⨉10^-8^ for ODEs that permit the best classification). Thus, the aligned trends explain the correlation with distance from the center of the overall inverted U, and consequently evinces a bifurcation-type effect because of the number of fixed points changes. For some cells, the preferred stimulus has fewer fixed points while for other cells it has more. Cell-to-cell variability is not the only factor, a similar factor is Ξ to Ξ variability. We independently train 45 different Ξ matrices for each cell and let the Ξ matrices “vote” on a correct classification. Hence, each Ξ matrix is a different model of dynamics, akin to a different “perspective”. Hence different Ξ matrices also show different trends. For this reason, we report on only the Ξ matrix which had the most optimal objective function value at the last generation of the genetic algorithm.

| 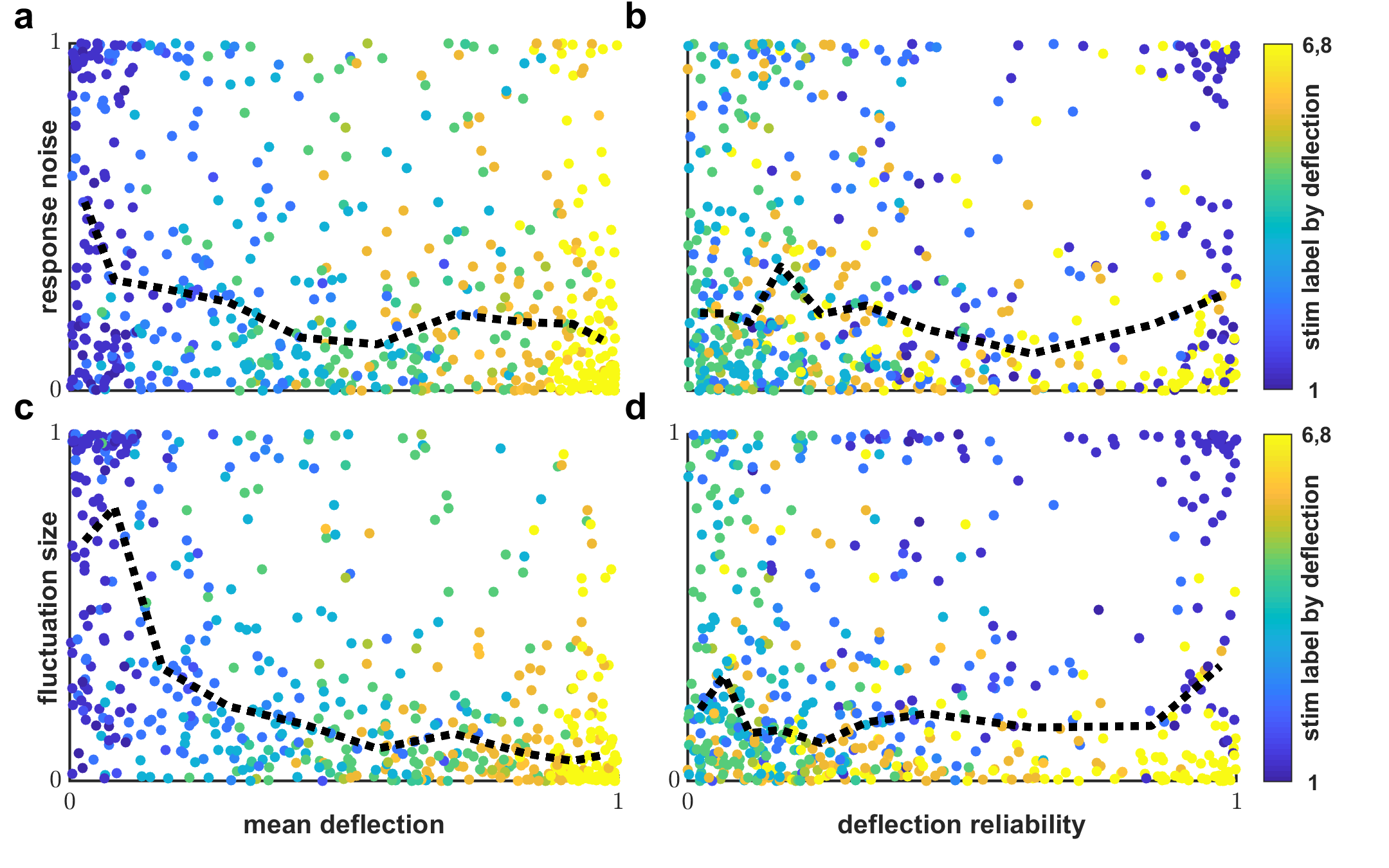 |
| --- |
| **Figure S9 \| Scatter plots showing how measures of noise and fluctuation size relate to deflection and reliability.** **a,** A normalized measure of noise, the mean residual error after subtracting mean stimulus-dependent response, is plotted against normalized mean deflection. Colors indicate stimulus labels according to deflection rank (color bar right of panels b and d). The black dashed line indicates central tendency: the median noise in 10 deciles of mean deflection. Unlike mean reliability, which showed a strong U-shaped trend (high reliability for extremes of deflection), noise decays with increasing deflection. **b,** Normalized noise is plotted against reliability. Colors are the same as in a, the dashed line are the median noise for 10 deciles of reliability. If reductions in noise alone caused greater reliability, we would expect to see a trend, and do not. **c,** A normalized measure of fluctuation size, the coefficient of variation for time-series points within the recording snippet, is plotted against normalized mean deflection. The pattern is very similar to that found in noise (panel a). Neither noise nor fluctuation size recapitulate the U-shape but it would be possible that they worked together if they showed opposite trends (e.g. low deflection high-reliability is due to small fluctuation while high-deflection high-reliability is due to low noise). However, both fluctuation size and noise are highest for low-deflection evoking stimuli therefore they do not work in combination. **d,** Normalized fluctuation size is plotted against normalized reliability. No correlation is found. |

In the main text we juxtaposed our limited findings about linear stability analysis of ODE fixed points with our finding that extremes of deflection evoked the most reliable deflections on average. This has a possible explanation in dynamics, whereby stimuli that evoke intermediate deflections do so because the dynamics they evoke is inherently less repeatable (e.g. sensitive dependence on initial conditions) or because they sometimes evoke the dynamics associated with the lowest deflection values and at other times they evoke the dynamics associated with the highest deflection values. Greater reliability would be due to attractors following similar trajectories. The trajectories themselves could cover any range of values and start at any point in their paths. Thus, one attractor could produce trajectories that display larger apparent fluctuations than another attractor, yet a path integration measure (e.g. deflection) on two of these trajectories would produce similar values (i.e. high reliability). Alternatively, two trajectories from the same attracting set could have high relative error because they start at different points (e.g. phase-shifted sinusoids), yet again in certain situations integration of the trajectory would yield similar values. A counter hypothesis is that the reliability is simply due to quenched variability in a random process that has similar governing dynamics in any situation. In this paradigm greater reliability would arise because either fluctuations are small or noise (relative error) is small. Hence, we measured fluctuation size and relative error (see methods). If either alone explains the reliability patterns we saw, then they would anti correlate strongly with reliability. Neither did (see Fig. S9b, S9d), our measure of fluctuation size (coefficient of variation), gave a Spearman correlation of r=-0.013, p=0.726, and our measure of noise gave r=-0.038, p=0.317. Noise and fluctuation size could work together to produce the reliability pattern if one measure was very low for small deflections and the other was very small for large deflections. This was not seen either, both strongly anticorrelated with deflection, our measure of fluctuation size (coefficient of variation), gave a Spearman correlation of r=-0.5, p=8.179⨉10^-45^, and our measure of noise gave r=-215, p=1.252⨉10^-8^. Because both measures strongly anti-correlated with deflection, instead of having a U-shaped trend or opposite trends, and because neither anticorrelated with reliability than an understanding founded on random-process origins would predict low reliability for small deflections and this was notably contradicted.

# S.4 Maximum Likelihood Estimation of stimulus reveals dimensionality expansion is not sufficient

One way to test whether dimensionality expansion alone (without dynamical systems) is enough without the dynamical systems perspective is to simply use the probability of a trajectory being limited to a region of state space. By plotting the carefully selected example trajectories seen in Fig. 2c we can assess whether all the information gleaned from dynamical discrimination is evinced more simply as the confinement of trajectories to regions of phase space, such as one conic surface nested inside another, or oscillating around centers that are displaced from one another. It is natural to describe these shapes with cylindrical coordinates. Although these patterns are visible, like deflection, they are highly variable and must be carefully assessed.

To capture the effects of dimensionality expansion without an ODE-based classifier we use a general method for trajectory classification, maximum likelihood estimation. This method acknowledges that no trajectory explores all of the available state space and that if any classification is possible then it must be that some regions of state space are more likely to be explored than others. So, it uses 75% of the recordings from one cell to learn the probability that a given point in state space will be occupied for each stimulus. For the remaining 25% of recordings each is assigned a probability that it co-occurred with each stimulus. The stimulus with the highest probability of co-occurrence is selected to be the prediction. As with dynamical discrimination, hold-out cross validation is repeated 510 times to get the average performance reported as final and presented in Fig. S10. Because the trajectories took the form of orbits confined to conic or cylindrical regions, we ignore the angular dimension and just using the radial and axial coordinates to create the probability density functions. This defines regions of state space that are annuli with rectangular radial cross sections.

The performance of this approach was roughly comparable to deflection based classification with overall classification rates exceeding chance (r_sdf_=0.225, p=2.04⨉10^-15^) and categories ***^I,C^I*** (r_sdf_=0.258, p=3.05⨉10^-5^), ***^E,C^I***(r_sdf_=0.207, p=0.0206), ***^R,C^V*** (r_sdf_=0.2476, p=0.0049), ***^I,S^I*** (r_sdf_=0.2538, p=1.14⨉10^-5^), and ***^E,S^I*** (r_sdf_=0.1922, p=0.0333) distinguishable from random chance (see Fig. S10c). This tells us that dimensionality expansion alone can reveal only a limited amount of additional information, and we get no additional information about orientation. This is likely due to the fact that regions of high density in the probability density maps overlap as seen in Fig. S10b, thus dimensionality expanded trajectories often have distinctive features that may be stimulus related they are not confined to easily separated regions of state space.

| **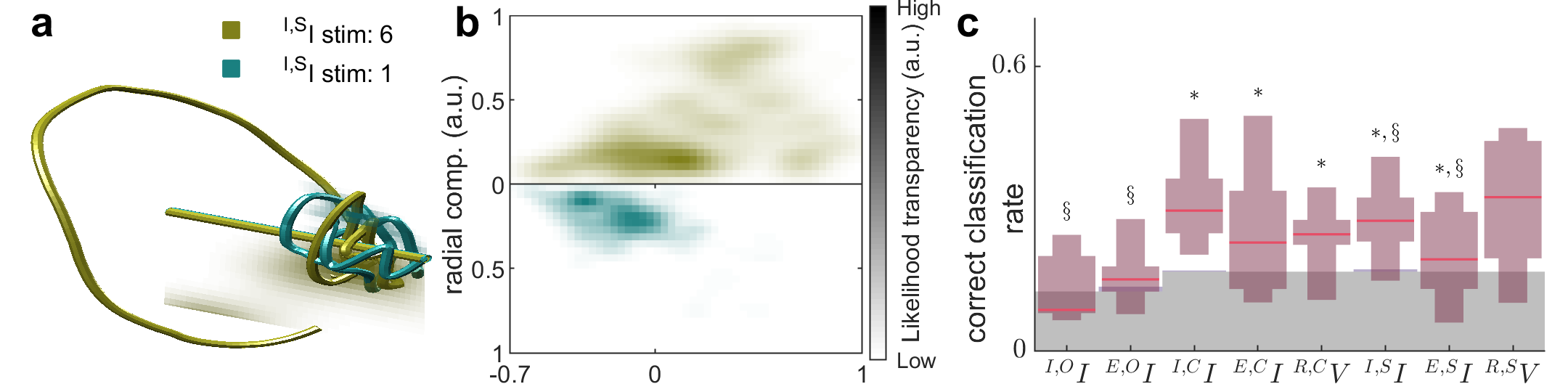** |
| --- |
| **Figure S10 \| Trajectories vary by stimulus and occupy different but non-separable regions of state space** **a,** A close view of two example trajectories. Aqua is an example of the least preferred stimulus and gold denotes the most preferred stimulus for the same cell. Synaptic inhibition was recorded while size varied. The reduced axial-radial cross section is shown as a shaded plane amidst the trajectories. This plane is most opaque in regions where the color-matched trajectory (aqua top, gold bottom) had a high likelihood of intersecting any given axial-radial cross section, this is known as the single trial axial-radial probability density map *M_t_(z,r;i)* (z is axial, r is radial, i denotes the trial index). **b,** The stimulus-dependent axial-radial probability density maps for the same two stimuli shown in b. Aqua (bottom) is *M_s_(z,r;1)*, gold (top) is *M_s_(z,r;6)*. This is created using all the trials coinciding with the selected stimuli. The distinctly conic region is apparent, as is the stimulus dependent nesting effect and the stimulus dependent axially translation for regions of maximal density. Selecting any single trial probability density map and selecting any stimulus-dependent probability map allows one to compute the likelihood that the single trial coincided with the selected stimulus. The method of Maximum Likelihood Estimation (MLE) amounts to trying all stimuli and finding the one with the highest likelihood. **c,** Same as in Fig. 3a except it is showing the classification results for MLE trajectory classification. Greater than chance performance is indicated with *, and § indicates significantly worse performance than dynamical discrimination (Fig. 3b). MLE is not better than deflection, indicating that the distinctive features seen in Fig. 2c do not make trajectories separable. |

# S.5 Closer look at dimensionality expansion of transmembrane current recordings and dependence on changes to orientation at the excitatory reverse potential

The transformation between single recording and their delay embedded counterparts is difficult to intuit. The nature of trajectories as oscillations confined to cylindrical or conic region is not guaranteed. In principle trajectories from delay embedding time series can take any form, including random walks (which would appear as filled spheres) and run-away divergence (if the time series also diverged). As seen in Fig. S11 the easiest feature of time-series that gets somewhat preserved in delay embedding is the magnitude of fluctuations. However, because we use singular value decomposition on a Hankel matrix of delays some smoothing is performed. This means that although the magnitude of fluctuations in one time series may be a factor of ten larger than another, the embedding is not necessarily ten time more expansive. As an example, compare Fig. S11c to Fig. S11e).

| 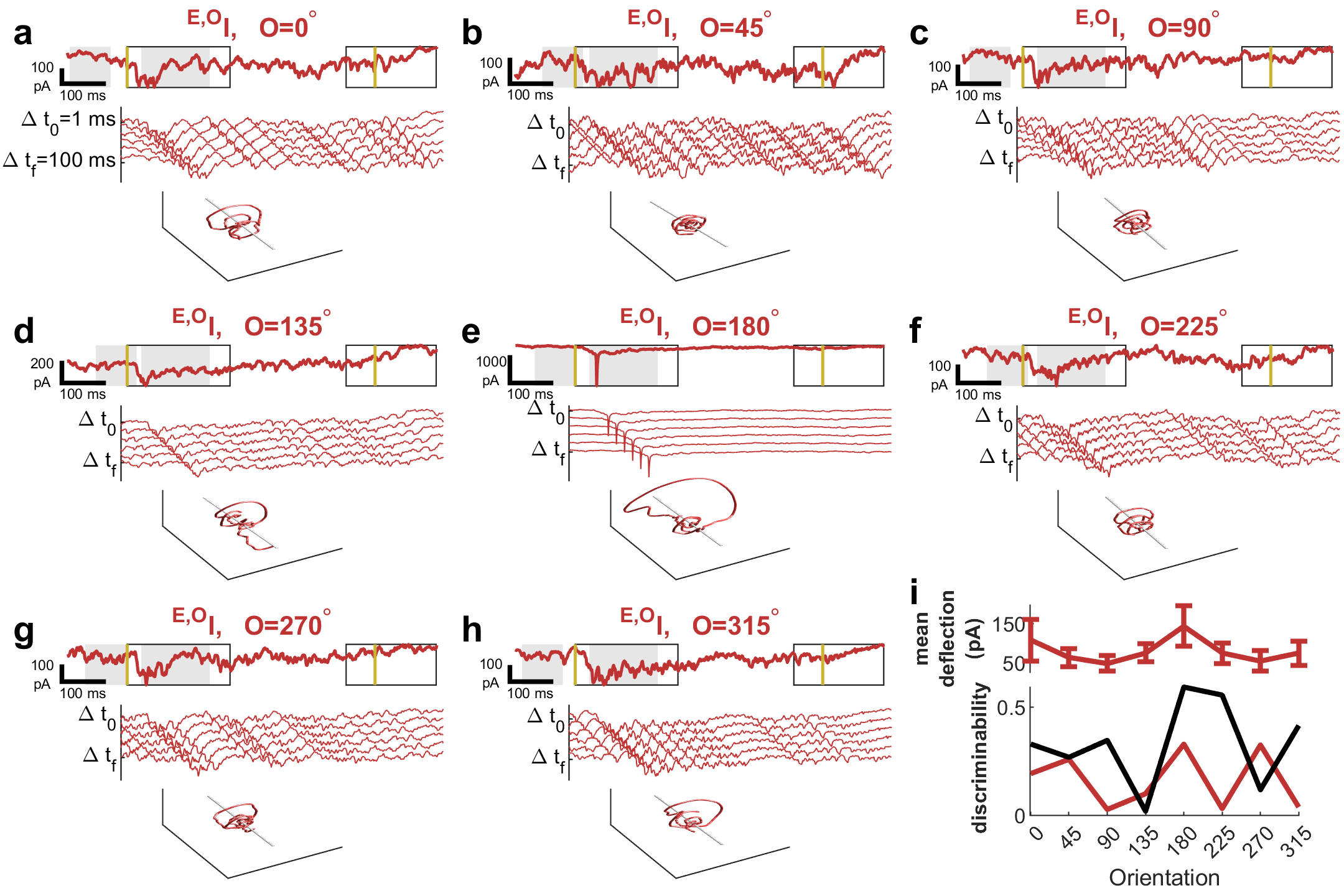 |
| --- |
| **Figure S11 \| Examples of trajectories, along with a tuning curve and its relation to discriminability. a-h,** Examples of original time series and delay embeddings. All depict recordings of transmembrane current at the excitatory reverse potential while drifting gratings of varying orientation were presented. The orientation of the drifting grating is written at the top of each panel. The top row of every panel features a neural time-series. With each time series the first gray shaded rectangle indicates the epoch where baseline was defined, and the second shaded rectangle shows where deflection was calculated from. The vertical gold bars mark the stimulus on and off times. The boxes (rectangular outlines) indicate the epochs defined as the early, “on” epoch and late, “off” epoch. The “full” epoch runs from the start of the first box to the end of the second. The second row of each panel plots 5 rows of the Hankel matrix used to calculate the delay embedding. Each row is a delayed version of the original time-series. The final and third row of each panel depicts the first three dimensions of a singular value decomposition performed on the Hankel matrix. This is a delay embedded trajectory. Each trajectory is plotted at the same scale. A gray cylinder marks the axis of rotation for that trajectory. Note panels c and e show the same trajectories from Fig. 2c (but include the full response). **i,** A comparison of the mean deflection response to the discriminability (F_1_ score) of each orientation. The top row shows deflection response with error bars indicating standard deviation. Below that the discriminability of each orientation according to deflection-based discrimination is plotted (red) alongside the same for dynamical discrimination (black). In general discriminability by two methods are not correlated. |

# S.6 SINDy captures dynamics well if dimensionality is retained

The Lorenz system used to drive our neural models has three dimensions. The neural models themselves have more dimensions. Early in the development of dynamical discrimination we investigated whether our delay embedding separated these dimensions or mixed them. We use two-dimensional ODE model neurons (the Fitz-Hugh Nagumo model) and drove it with sinusoidal current. A sinusoid is two-dimensional ODE. This makes a nominally four-dimensional system. We varied the strength of sinusoidal input. We never observed a situation where there were clear sinusoidal dynamics in two delay embedded dimensions and Fitz-Hugh Nagumo dynamics in a different two (by visual inspection of trajectories and fitted Ξ matrices). The fourth dimension of our delay embeddings had very small magnitude fluctuations. We interpreted this to mean that delay embedding mixes, rather than separates dimensions. The Whitney and Takens delay embedding theorems conclude that autonomous systems of dimension D can be captured in no more than 2D+1 dimensions, there is always the possibility that fewer dimensions will capture a significant fraction of the topology of the dynamics. In section S2 we discussed the results of testing up to seven dimensions for dynamical discrimination. We did not see an improvement in performance commensurate with the increase in computational complexity. However, if the goal is to find a best-fit ODE model performance is improved according to our initial in-silico experiments. In Fig. S12 we demonstrate that if we use the original three-dimensional Lorenz dynamics and apply our genetic algorithm augmentation of SINDy in its best-fit Ξ configuration then we can do an excellent job at producing ODE models that reproduce the original Lorenz dynamics. However, if we inject Lorenz dynamics into model neurons the ability to find accurate ODE models greatly reduces with increasingly complex models. When our genetic algorithm augmentation of SINDy in its dynamical discrimination configuration the ODE models are not intended to me accurate, but nonetheless share a few qualities with the original trajectory.

| 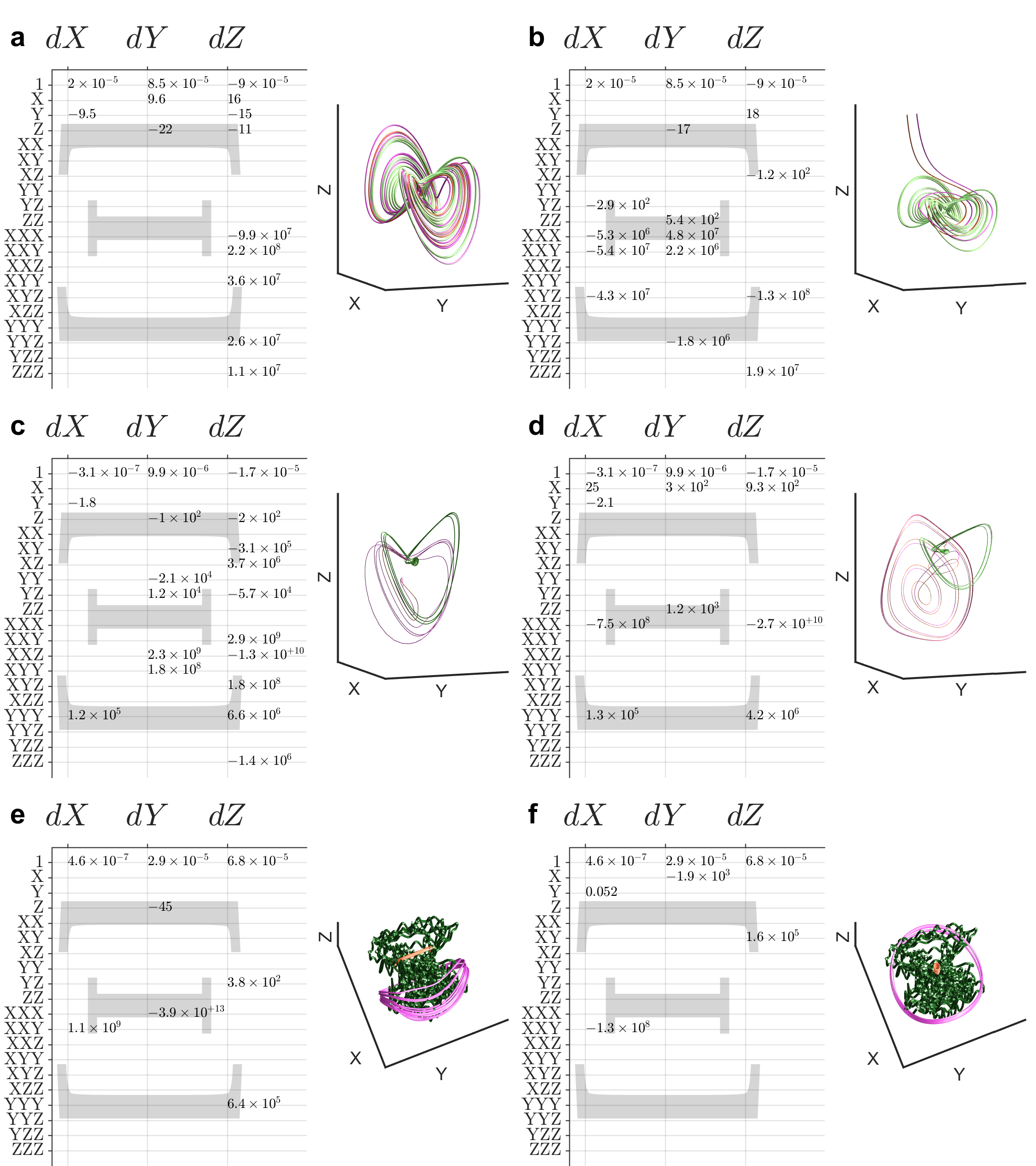 |
| --- |
| **Figure S12 \| ODE models fitted to Lorenz dynamics and neuron models driven by Lorenz dynamics capture the trajectories with varying degrees of success depending on the objective function used to learn model structure.** **a,** A Ξ matrix from a genetic algorithm optimizing trajectory modeling (best-fit Ξ). The Ξ was trained on Lorenz dynamics where the parameter ρ was varied between the integers 20 to 40. Prior to delay embedding the X dimension of Lorenz dynamics was unmodified except for rescaling. Left: Ξ coefficients learned from the trajectory on the right. Right: A delay embedded trajectory (green) and reconstructions (magenta/yellow). **b,** This Ξ is optimized for regression on the true ρ parameter (similar to the dynamical discrimination objective function). Same plotting style as a. **c and d,** The same as in panels a and b except that, prior to delay embedding, the X dimension of Lorenz dynamics was used as injected current to drive a single compartment neuron with Hodgkin Huxley dynamics. The resulting membrane potential had spikes removed and was delay embedded and is shown in medium-green. **e and f,** The same as in panels a and b except that, prior to delay embedding, the X dimension of Lorenz dynamics was used to drive the synapse firing rate for the synapses of a morphologically complex multi-compartment neuron. The resulting membrane potential had spikes removed and was delay embedded and is shown in dark-green. |

# S.7 Survey of tuning curves and dynamical discriminability

As discussed in the main text, the shape of the tuning curve assumed to underlie the deflection responses we calculate has a large impact on the ability to discriminate small changes in stimuli. Variability also plays a large role. In order to demonstrate what these tuning curves look like and to relate them to stimulus discriminability (F_1_ score) by dynamical discrimination we plotted the tuning curves for all cells included in the analysis. There, (Figures S13-S18) we can see that the most and least preferred stimuli tend to be the most discriminable, but that discriminability does not closely mirror trends in variability or cross-trial average deflection.

| 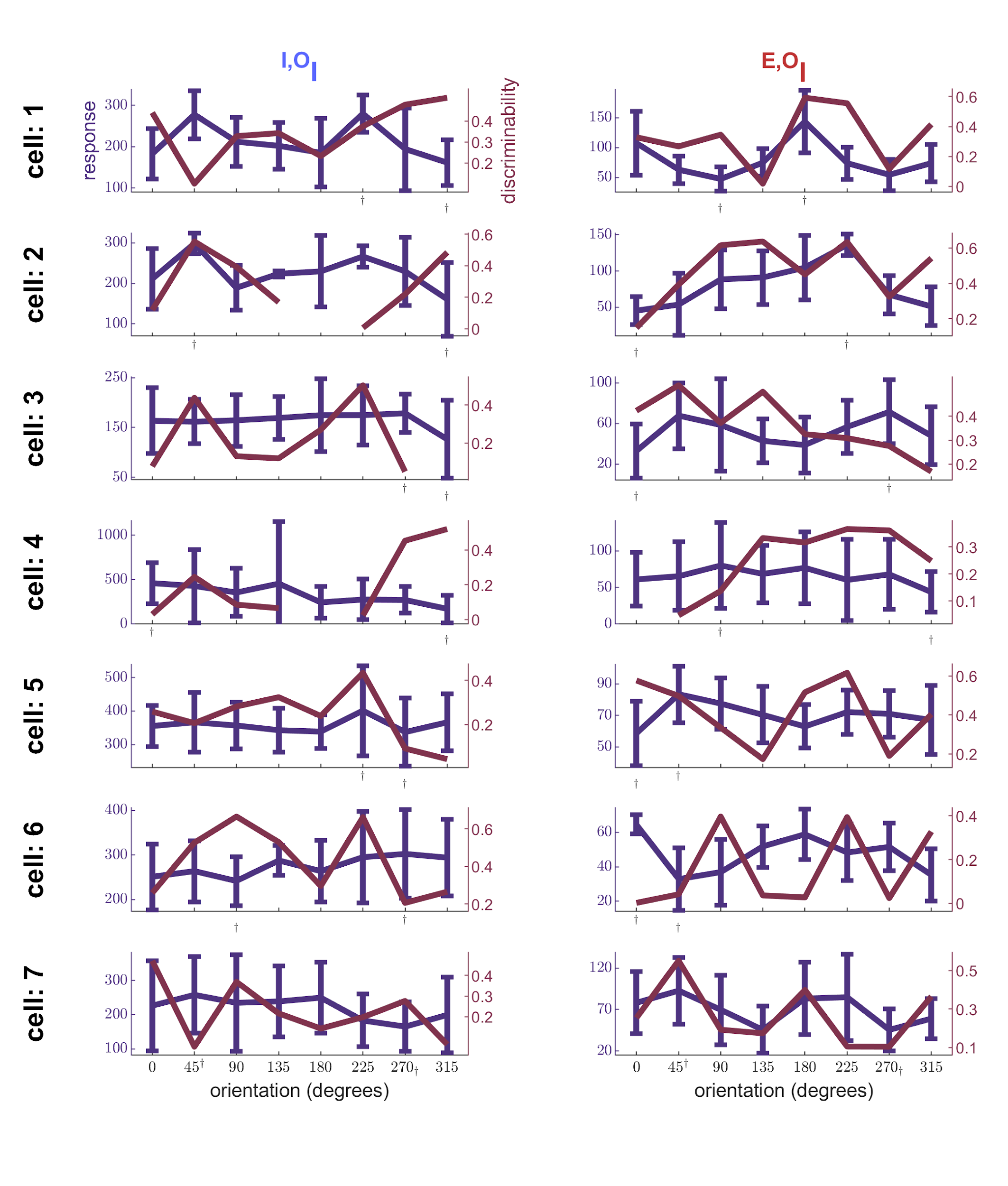 |
| --- |
| **Figure S13 \| Tuning curves and discriminability of each stimulus according to dynamical discrimination is plotted for each cell from the ^I,O^I and ^E,O^I data groups.** Each plot shows data from one cell. The cell index number is noted on the far left of each row of plots. These are the same cells in the same order as **^I,O^I** and **^E,O^I** (left and right columns respectively). In each plot Average deflection (pA) (purple, error bars indicate standard deviation) and discriminability (dark red, F_1_ score of dynamical discrimination) as a function of the drifting grating orientation denoted on the abscissa of the last plot in each column. The least and most preferred stimuli are indicated with _†_ and ^†^ respectively. |

| 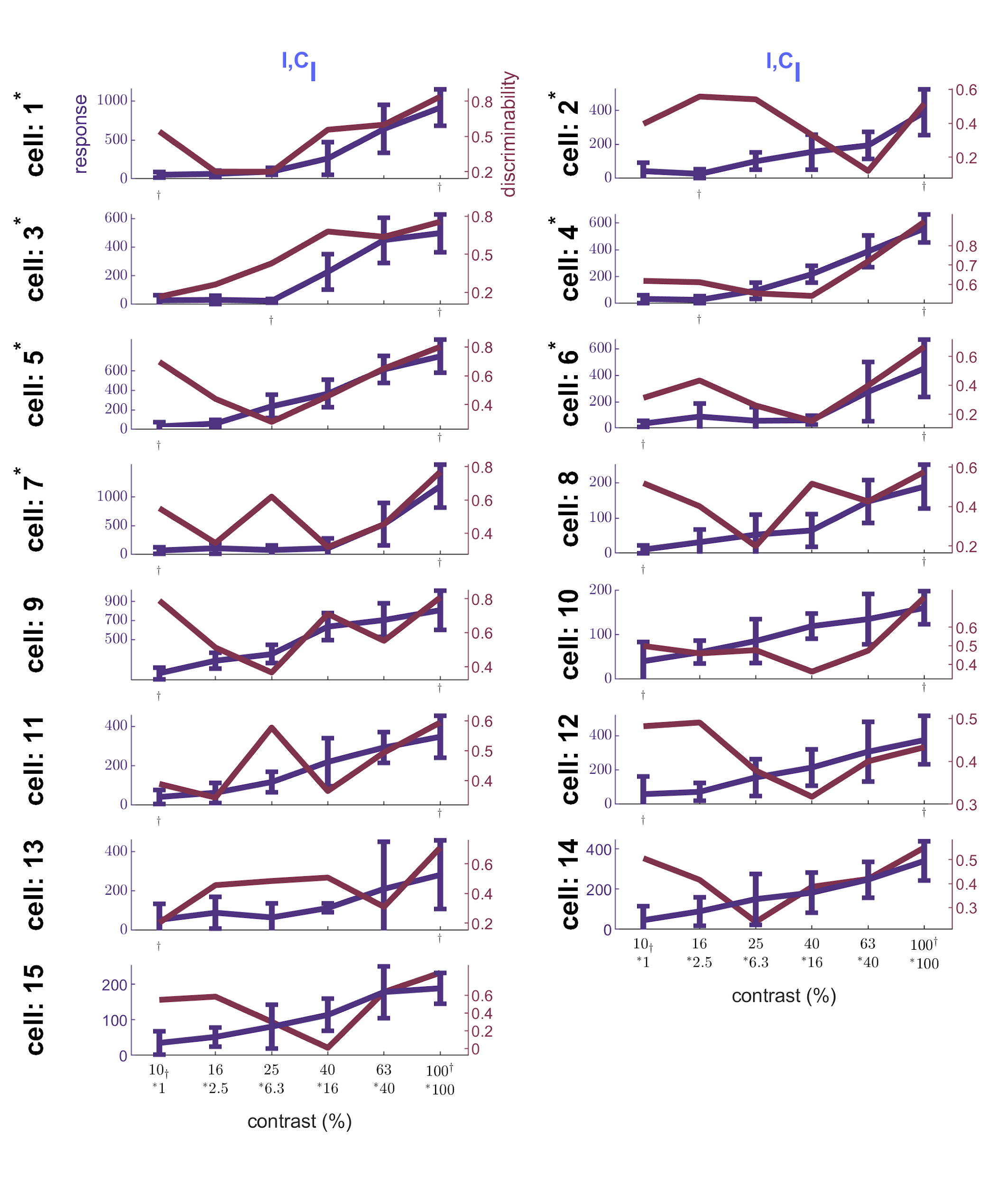 |
| --- |
| **Figure S14 \| Tuning curves and discriminability of each stimulus according to dynamical discrimination is plotted for each cell from the ^I,C^I data groups.** Each plot shows data from one cell. The cell index number is noted on the far left of individual plots. These are the same cells in the same order as **^E,C^I** (Fig. S14). In each plot Average deflection (pA) (purple, error bars indicate standard deviation) and discriminability (dark red, F_1_ score of dynamical discrimination) as a function of the drifting grating contrast. 7/15 cells used a different set of contrasts than the remaining 8/15. Both sets of contrast are denoted on the abscissa of the last plot in each column. If the cell index is marked with an ^*^ refer to the contrast level also marked with an ^*^. The least and most preferred stimuli are indicated with _†_ and ^†^ respectively. |

| 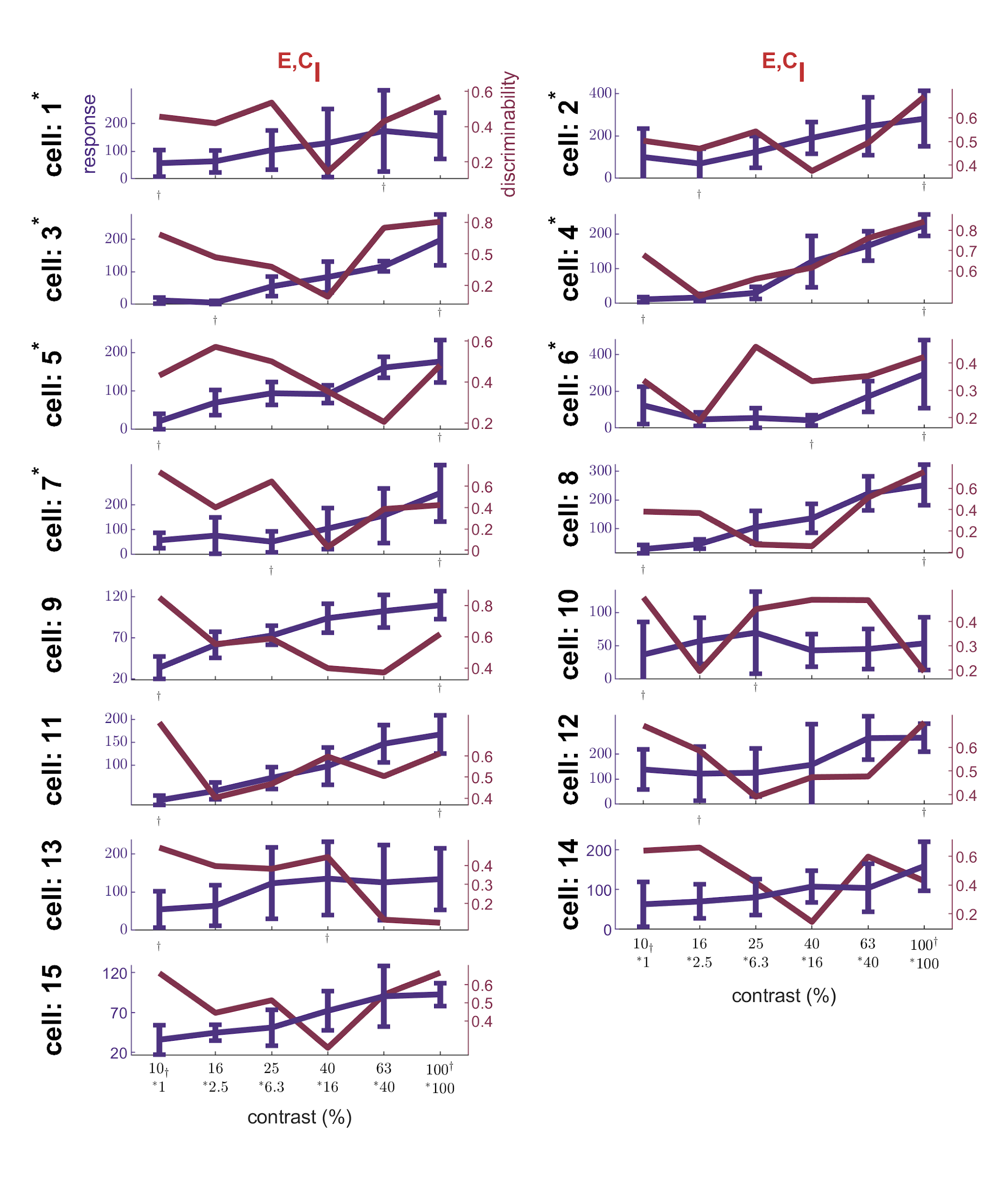 |
| --- |
| **Figure S15 \| Tuning curves and discriminability of each stimulus according to dynamical discrimination is plotted for each cell from the ^E,C^I data groups.** Each plot shows data from one cell. The cell index number is noted on the far left of individual plots. These are the same cells in the same order as **^I,C^I** (Fig. S14). In each plot Average deflection (pA) (purple, error bars indicate standard deviation) and discriminability (dark red, F_1_ score of dynamical discrimination) as a function of the drifting grating contrast. 7/15 cells used a different set of contrasts than the remaining 8/15. Both sets of contrast are denoted on the abscissa of the last plot in each column. If the cell index is marked with an ^*^ refer to the contrast level also marked with an ^*^. The least and most preferred stimuli are indicated with _†_ and ^†^ respectively. |

| 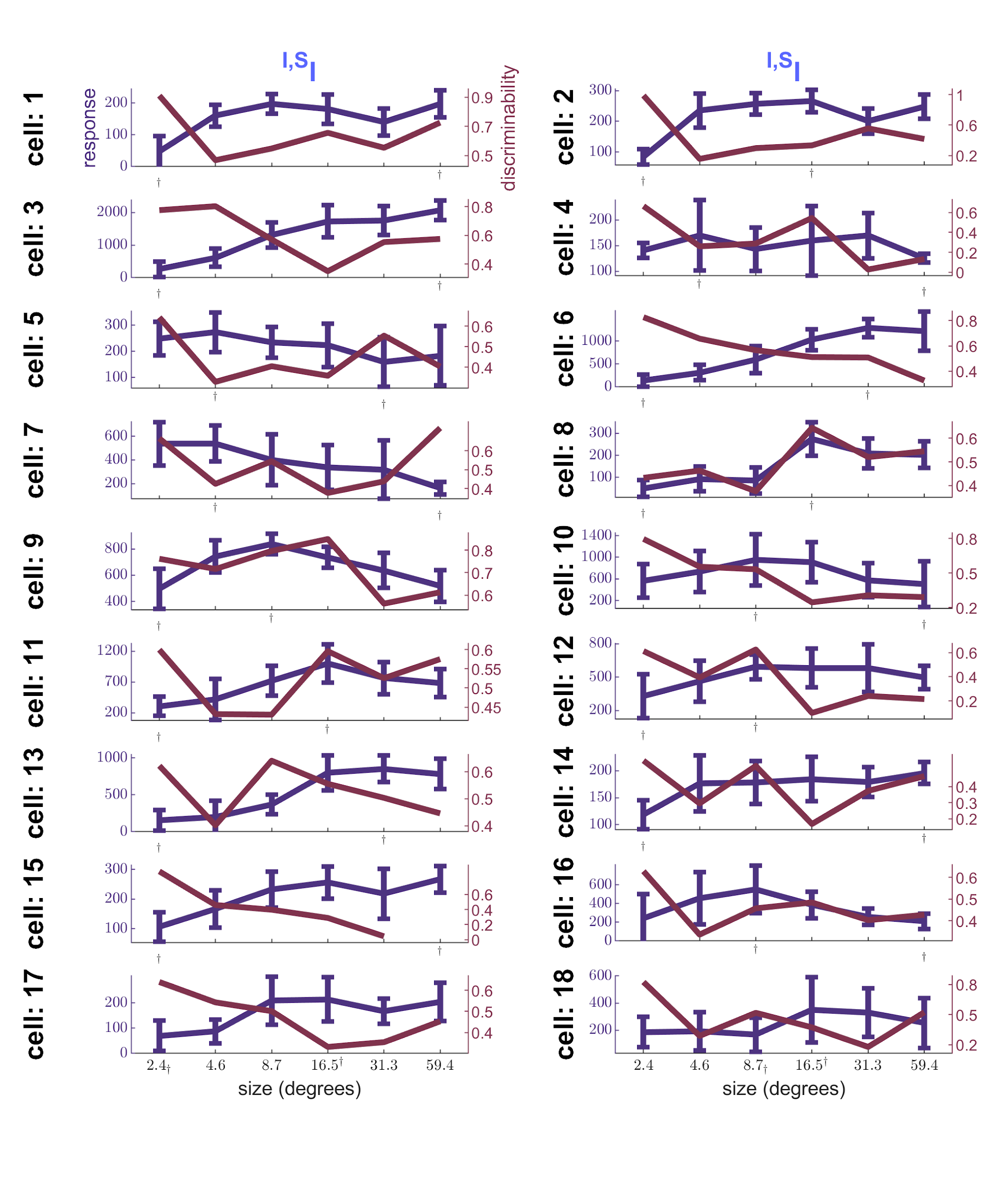 |
| --- |
| **Figure S16 \| Tuning curves and discriminability of each stimulus according to dynamical discrimination is plotted for each cell from the ^I,S^I data groups.** Each plot shows data from one cell. The cell index number is noted on the far left of individual plots. These are the same cells in the same order as **^E,S^I** (Fig. S17). In each plot Average deflection (pA) (purple, error bars indicate standard deviation) and discriminability (dark red, F_1_ score of dynamical discrimination) as a function of the drifting grating size denoted on the abscissa of the last plot in each column. The least and most preferred stimuli are indicated with _†_ and ^†^ respectively. |

| 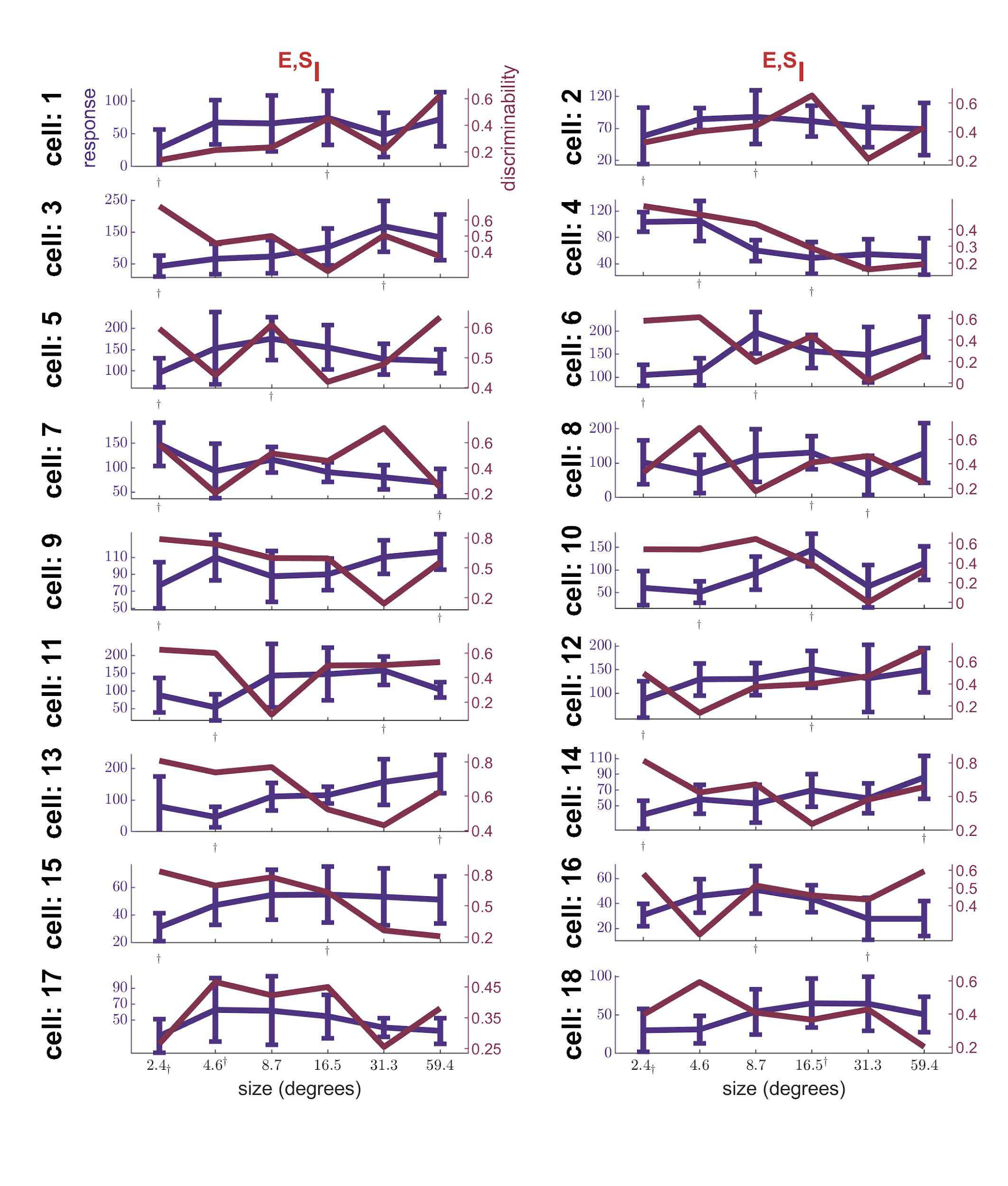 |
| --- |
| **Figure S17 \| Tuning curves and discriminability of each stimulus according to dynamical discrimination is plotted for each cell from the ^E,S^I data groups.** Each plot shows data from one cell. The cell index number is noted on the far left of individual plots. These are the same cells in the same order as **^I,S^I** (Fig. S16). In each plot Average deflection (pA) (purple, error bars indicate standard deviation) and discriminability (dark red, F_1_ score of dynamical discrimination) as a function of the drifting grating size denoted on the abscissa of the last plot in each column. The least and most preferred stimuli are indicated with _†_ and ^†^ respectively. |

| 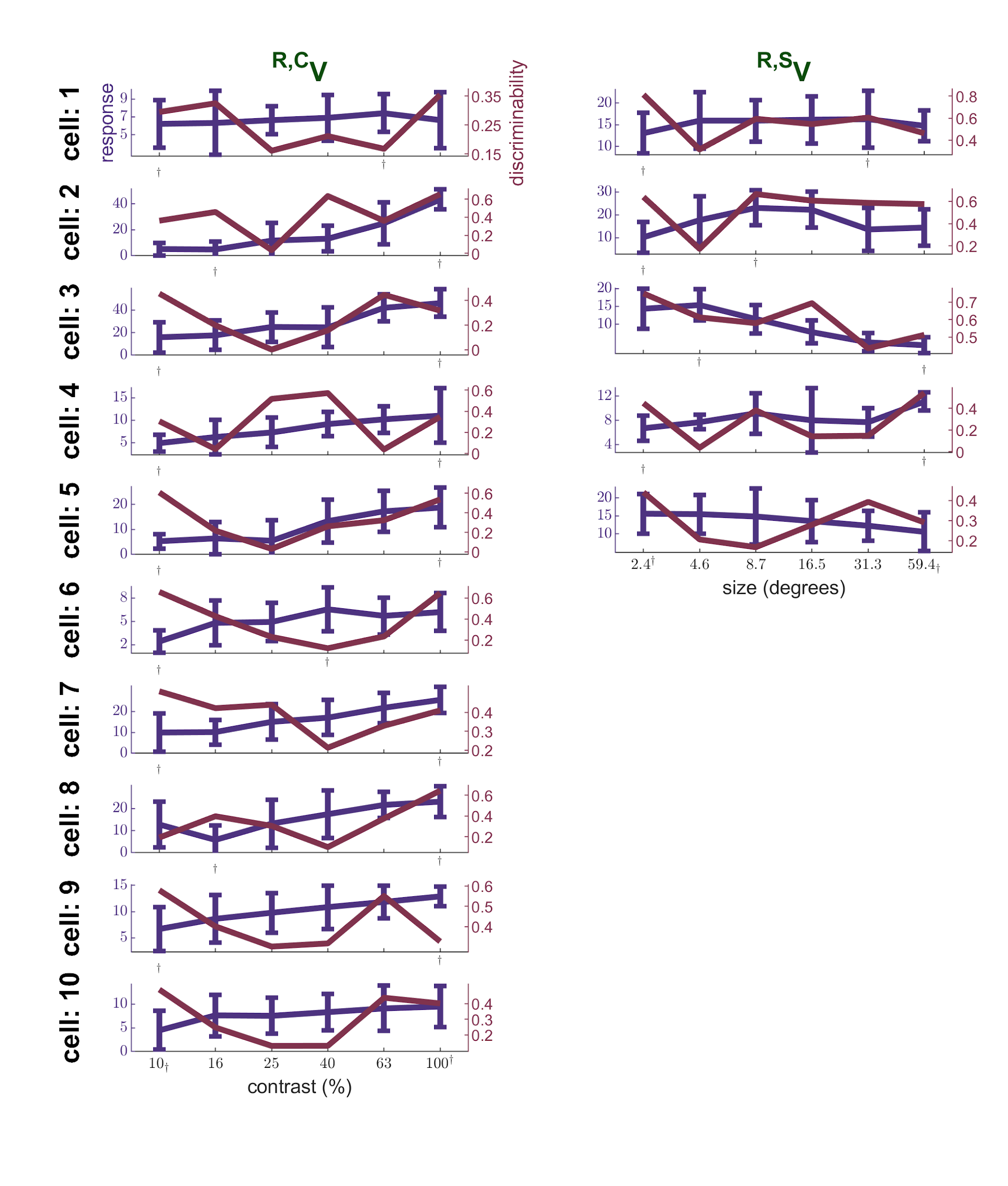 |
| --- |
| **Figure S18 \| Tuning curves and discriminability of each stimulus according to dynamical discrimination is plotted for each cell from the ^R,C^V and ^R,S^V data groups.** Each plot shows data from one cell. The cell index number is noted on the far left of each row of plots and does not indicate inclusion across categories. No cell from **^R,C^V** is included in **^R,S^V**. In each plot Average deflection (mV) (purple, error bars indicate standard deviation) and discriminability (dark red, F_1_ score of dynamical discrimination) as a function of the drifting grating property denoted on the abscissa of the last plot in each column (contrast, on left column, and size on the right column). The least and most preferred stimuli are indicated with _†_ and ^†^ respectively. |

# References to supplemental

1. Maharaj, E. A., D’Urso, P. & Caiado, J. *Time Series Clustering and Classification*. (Chapman and Hall/CRC, 2019). doi:10.1201/9780429058264.

2. Lee, J.-G., Han, J., Li, X. & Gonzalez, H. TraClass: trajectory classification using hierarchical region-based and trajectory-based clustering. *Proceedings VLDB Endowment* **1**, 1081–1094 (2008).

3. Hough, P. V. C. Machine analysis of bubble chamber pictures. in *Conf. Proc.* vol. 590914 554–558 (inspirehep.net, 1959).

4. Saha, D. *et al.* A spatiotemporal coding mechanism for background-invariant odor recognition. *Nat. Neurosci.* **16**, 1830–1839 (2013).

5. Mazor, O. & Laurent, G. Transient dynamics versus fixed points in odor representations by locust antennal lobe projection neurons. *Neuron* **48**, 661–673 (2005).

6. Laurent, G. Olfactory network dynamics and the coding of multidimensional signals. *Nat. Rev. Neurosci.* **3**, 884–895 (2002).

7. Pandarinath, C. *et al.* Inferring single-trial neural population dynamics using sequential auto-encoders. *Nat. Methods* **15**, 805–815 (2018).

8. Gallego, J. A., Perich, M. G., Miller, L. E. & Solla, S. A. Neural Manifolds for the Control of Movement. *Neuron* **94**, 978–984 (2017).

9. Churchland, M. M. *et al.* Neural population dynamics during reaching. *Nature* **487**, 51–56 (2012).

10. Kawakami, K. Supervised sequence labelling with recurrent neural networks. *Ph. D. dissertation, PhD thesis. Ph. D* (2008).

11. Kaiser, E., Kutz, J. N. & Brunton, S. L. Sparse identification of nonlinear dynamics for model predictive control in the low-data limit. *Proc. Math. Phys. Eng. Sci.* **474**, 20180335 (2018).

12. Sauer, T., Yorke, J. A. & Casdagli, M. Embedology. *J. Stat. Phys.* **65**, 579–616 (1991).

13. Strogatz, S. H. *Nonlinear dynamics and chaos: with applications to physics, biology, chemistry, and engineering*. (CRC Press, 2018).

14. Breiman, L. Random Forests. *Mach. Learn.* **45**, 5–32 (2001).

15. Wolfe, J., Houweling, A. R. & Brecht, M. Sparse and powerful cortical spikes. *Curr. Opin. Neurobiol.* **20**, 306–312 (2010).

16. Butts, D. A. & Goldman, M. S. Tuning curves, neuronal variability, and sensory coding. *PLoS Biol.* **4**, e92 (2006).

17. Carandini, M. & Ferster, D. Membrane potential and firing rate in cat primary visual cortex. *J. Neurosci.* **20**, 470–484 (2000).

18. Ferster, D. Orientation selectivity of synaptic potentials in neurons of cat primary visual cortex. *J. Neurosci.* **6**, 1284–1301 (1986).

19. Adesnik, H. Synaptic Mechanisms of Feature Coding in the Visual Cortex of Awake Mice. *Neuron* **95**, 1147–1159.e4 (2017).

20. Wilcoxon, F. Individual Comparisons by Ranking Methods. in *Breakthroughs in Statistics: Methodology and Distribution* (eds. Kotz, S. & Johnson, N. L.) 196–202 (Springer New York, 1992). doi:10.1007/978-1-4612-4380-9_16.

21. Kerby, D. S. The Simple Difference Formula: An Approach to Teaching Nonparametric Correlation. *Comprehensive Psychology* **3**, 11.IT.3.1 (2014).

22. Keller, G. B. & Mrsic-Flogel, T. D. Predictive Processing: A Canonical Cortical Computation. *Neuron* **100**, 424–435 (2018).

23. Miconi, T., McKinstry, J. L. & Edelman, G. M. Spontaneous emergence of fast attractor dynamics in a model of developing primary visual cortex. *Nat. Commun.* **7**, 13208 (2016).

24. Bondanelli, G. & Ostojic, S. Coding with transient trajectories in recurrent neural networks. *arXiv [q-bio.NC]* (2018).

25. Müller, J. R., Metha, A. B., Krauskopf, J. & Lennie, P. Information conveyed by onset transients in responses of striate cortical neurons. *J. Neurosci.* **21**, 6978–6990 (2001).

26. Izhikevich, E. M. & FitzHugh, R. Fitzhugh-nagumo model. *Scholarpedia J.* **1**, 1349 (2006).
